# Binding Between Cyclohexanohemicucurbit[*n*]urils and Polar Organic Guests

**DOI:** 10.3389/fchem.2021.701028

**Published:** 2021-06-28

**Authors:** Lukas Ustrnul, Tatsiana Burankova, Mario Öeren, Kristina Juhhimenko, Jenni Ilmarinen, Kristjan Siilak, Kamini A. Mishra, Riina Aav

**Affiliations:** ^1^ Department of Chemistry and Biotechnology, School of Science, Tallinn University of Technology, Tallinn, Estonia; ^2^ Process Analytics, Hamilton Bonaduz AG, Bonaduz, Switzerland; ^3^ Optibrium Limited, Cambridge, United Kingdom

**Keywords:** supramolecular chemistry, binding evaluation, hemicucurbit[n]uril, hydrogen bond, complex stoichiometry, NMR, partial charge, organic acid

## Abstract

Inherently chiral, barrel-shaped, macrocyclic hosts such as cyclohexanohemicucurbit[*n*]urils (cycHC[*n*]) bind zinc porphyrins and trifluoroacetic acid externally in halogenated solvents. In the current study, we tested a set of eighteen organic guests with various functional groups and polarity, namely, thiophenols, phenols, and carboxylic and sulfonic acids, to identify a preference toward hydrogen bond–donating molecules for homologous cycHC[6] and cycHC[8]. Guests were characterized by Hirshfeld partial charges on acidic hydrogens and their binding by ^1^H and ^19^F NMR titrations. Evaluation of association constants revealed the complexity of the system and indirectly proved an external binding with stoichiometry over 2:1 for both homologs. It was found that overall binding strength is influenced by the stoichiometry of the formed complexes, the partial atomic charge on the hydrogen atom of the hydrogen bond donor, and the bulkiness of the guest. Additionally, a study on the formation of complexes with halogen anions (Cl^−^ and Br^−^) in methanol and chloroform, analyzed by ^1^H NMR, did not confirm complexation. The current study widens the scope of potential applications for host molecules by demonstrating the formation of hydrogen-bonded complexes with multisite hydrogen bond acceptors such as cycHC[6] and cycHC[8].

## Introduction

Cucurbiturils and hemicucurbiturils are a large family of urea-based macrocycles (Lagona et al., 2005; Assaf and Nau, 2014; Andersen et al., 2018) that went through a rapid expansion during the last three decades—from the research of reaction conditions for selective synthesis of cucurbiturils of a specific size (Kim et al., 2000; Day et al., 2001) to a variety of uses in supramolecular catalysis, material chemistry, drug delivery, chemical sensors, or ion transport, to name a few (Walker et al., 2011; Barrow et al., 2015; Ji et al., 2019; Davis et al., 2020; Tang et al., 2020). Hemicucurbiturils have monomeric units connected by one row of methylene bridges and are mainly known for their ability to bind anions strongly in various solvents, which can be utilized for anion recognition and transport (Kaabel and Aav, 2017; Andersen et al., 2018; Lizal and Sindelar, 2018; Reany et al., 2018). In Aav’s group, the first enantiomerically pure, inherently chiral member of the family—specifically cyclohexanohemicucurbit[6]uril (cycHC[6]) (Aav et al., 2013)—was prepared. Later, its larger homologs, cycHC[8] (Prigorchenko et al., 2015) and cycHC[12] (Mishra et al., 2020), were also made.

**FIGURE 1:**
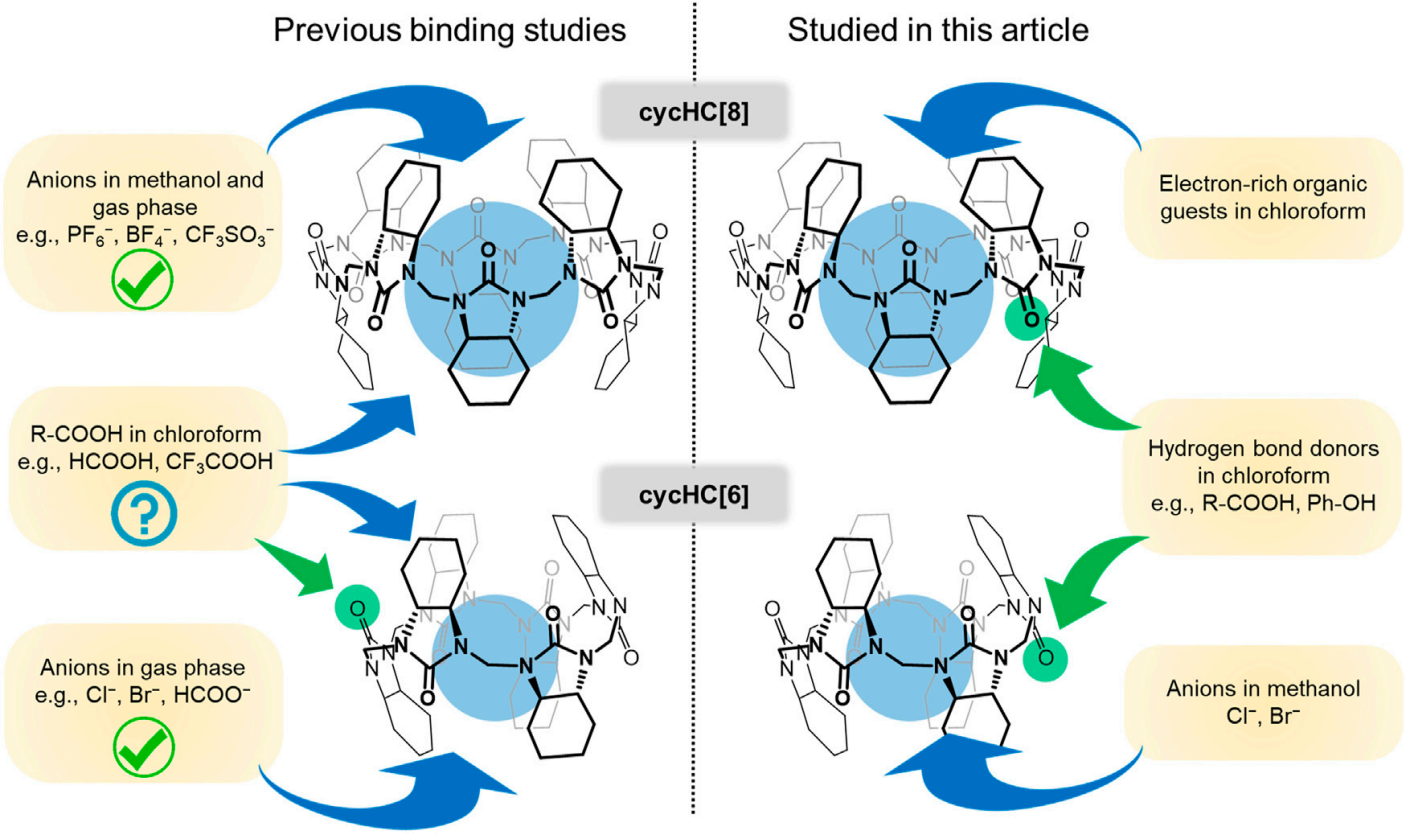
Depiction of previously described binding of various guests to cycHC[*n*] **(left)** and supramolecular interactions studied in this article **(right)**.

First studies of cycHC[6] evaluated the binding of various carboxylic acids in chloroform using the 1:1 binding model based on DOSY NMR and ^13^C NMR data (Aav et al., 2013). In this first study of cycHC[6]s and its binding, dependence on the size and shape of the guest was observed; therefore, the formation of inclusion complexes was proposed (Figure 1). The same approach was used to study complexes of cycHC[8], and the obtained association constants were analogous to results obtained for cycHC[6], which has a cavity volume that is roughly three times smaller (Prigorchenko et al., 2015). Such a result did not support the theory of inclusion complex formation as it should show a significant difference between cycHC[6] and cycHC[8] for the binding of the same guest. Hence, it was concluded that the complex formation depended on the acidity of the guest. Moreover, a computation and ion-mobility–mass spectrometry (IM-MS) study for cycHC[6] concluded that the macrocycle prefers to interact with the non-dissociated acids through their electron-poor hydrogen without forming an inclusion complex (Öeren et al., 2014). It should be noted that the study did confirm the formation of inclusion complexes with Cl^–^, Br^–^, and HCOO^–^, and these theoretical studies were validated with gas-phase IM-MS.

CycHC[*n*] macrocycles were obtained from a template (anion)-driven synthesis in polar media; therefore, the binding of inorganic anions in methanol was described in detail for cycHC[8] (Kaabel et al., 2017). A discovered preference for encapsulation of large symmetrical anions (e.g., SbF_6_^−^, PF_6_^−^ > BF_4_^−^, CF_3_SO_3_^−^, and AcO^−^) was later beneficial in the development of the solid-state synthesis of cycHC[*n*]s (Kaabel et al., 2019).

In 2019, it was demonstrated that cycHC[*n*] exhibits binding to zinc(II) porphyrins in chlorinated solvents *via* coordination with carbonyl oxygen on their outer surface (Ustrnul et al., 2019). At the same time, a study of *inverted*-cycHC[6] confirmed that strong organic acids are also bound from outside (Figure 1) with stoichiometry assumed to be 2:1 (acid:cycHC[6]) (Prigorchenko et al., 2019). These studies have shown the dependence of association constants on the geometry and size of the host. Notably, the significantly larger binding strength of guests with cycHC[6] (*K*_*1*_ = 10^2^–10^3^ M^−1^; *K*_*2*_ = 10^2^ M^−1^) than the binding strength of the same guests with monomeric urea (*N,N'*-dimethylcyclohexadiylurea) (*K*_*1*_ = 10^1^ M^−1^) was observed, which highlights the superiority of these multifunctional macrocycles compared to their monofunctional counterparts. The results on the binding of acids have motivated us to look deeper into and revise previous conclusions about binding and broaden the scope of guest molecules tested in chlorinated solvents.

Herein, we report several experiments increasing the knowledge about cycHC[*n*]-binding properties and bringing clarity on the binding of carboxylic acids in chlorinated solvents. A set of guests bearing electron-rich functional groups and their interaction with chiral cycHC[8] were tested. The binding of polar hydrogen bond (HB)-donating guests was studied mainly with cycHC[6], and we discuss difficulties related to stepwise association constant evaluation for systems with multiple binding sites. Additionally, we have examined the incorporation of small anions into the cycHC[6] cavity in methanol and chloroform solutions.

## Materials and Methods

### Materials, Instrumentation, and Sample Preparation

All reagents and solvents were purchased from commercial suppliers. Macrocyclic host compounds were used only as (*R*,*R*)-cycHC[*n*] enantiomers and were synthesized in our laboratory from (*R*,*R*)-cyclohexanourea according to procedures described in the literature (Aav et al., 2013; Prigorchenko et al., 2015; Kaabel et al., 2019).

^1^H NMR (400 MHz) and ^19^F NMR (376.5 MHz) spectra were recorded on a Bruker Avance III spectrometer, using a Bruker BBO probe equipped with a z-gradient coil. Chemical shifts were referenced to the residual proton solvent peak (*δ*(^1^H) = 3.34 ppm in CD_3_OD-*d*_*4*_ and *δ*(^1^H) = 7.26 ppm in CDCl_3_) or to TMS (0.00 ppm) as an internal standard. All chemical shifts are reported in ppm units. The data were analyzed using the program MNova (Mestrelab).

All the solutions were prepared using Hamilton® Gastight syringes; these syringes were also used for all additions during titrations. In the case of precise measurements of higher volumes (over 1 ml), the mass and density of the solvent were used instead of volumetric glassware. Samples were weighed on a microbalance with an accuracy of 6 μg (Radwag® MYA 11.4Y, Poland).

### Anion Binding With CycHC[6]

The binding of anions to cycHC[6] was tested in CD_3_OD-*d*_*4*_ (0.8 mM cycHC[6]) and CDCl_3_ (1.2 mM cycHC[6]) by the addition of salt excess to the macrocycle solution. Tetrabutylammonium (TBA) chloride and bromide have been added as a solid compound. The specific excess of salt was determined from the integration of NMR signals against the known concentration of macrocycle. ^1^H NMR was measured shortly before and after salt addition and then after 18 h. The dissolution of weakly soluble cycHC[6] in methanol was achieved by heating the sample repeatedly and employing sonification.

### Screening of Potential Guests, Titrations, and Job’s Plot in CDCl_3_

In the first screening of guests, chemical shift changes of cycHC[8] (ca 2.5 mM) proton signals induced by the addition of 0.5, 5, and 40 equivalents (equiv) of guests (**1–9**) were investigated by ^1^H NMR in CDCl_3_. Guests were added in solutions of known concentration (typically 300–400 mM).

All NMR titrations were performed at a constant concentration of guest (2 mM for qualitative comparison titrations, guests **6, 9–18**) in a sample throughout the whole experiment, which was achieved by the dissolution of titrant (cycHC[*n*]) in the solution of the guest. ^1^H or ^19^F NMR was used according to the guest’s structure; ^19^F NMR was preferred as it usually revealed more significant changes in the chemical shift.

The continuous variation method (Job’s plot) was conducted for trifluoroacetic acid (guest **16**) with cycHC[8] (10 mM) and for **16** with cycHC[6] (20 mM).

### Binding Strength Evaluation and Partial Charge Calculation

Stepwise association constants were evaluated and simulated using online tools at supramolecular.org (Thordarson, 2011; Hibbert and Thordarson, 2016) and our 3:1 binding model, which was introduced in our previous publication (Ustrnul et al., 2019). The script for the 3:1 binding model uses the NumPy (1.10.2) and SciPy (0.18.1) libraries of Python 3. The script was adapted to the studied system of HB donors and macrocycles using the following constraints: 1) *K*_*1obs*_ > *K*_*2obs*_ > *K*_*3obs*_ and 2) *δ*_HG1_, *δ*_HG2_, *δ*_HG3_ ≥ *δ*_EXPmax_− 5 (*δ*_EXPmax_ − *δ*_EXPmin_); these constraints prevent the chemical shift *δ* of the complexed guest (HG_x_) from diverging extremely from experimentally observed values.

The atomic charges presented in the study are calculated using the modified Hirshfeld charge analysis. The wave function for the population analysis was obtained using the density functional theory (DFT) (Lu and Chen, 2012; Aprà et al., 2020).

## Results and Discussion

### Test of Anion Binding Inside CycHC[6] in Methanol Solutions

Precisely described anion binding inside the cycHC[8] cavity in methanol solutions (Kaabel et al., 2017) and previously calculated and experimentally confirmed (in gas phase) complexes of Cl^−^, Br^−^, and HCOO^−^ with cycHC[6] (Öeren et al., 2014) would imply a possible interaction between a 6-membered macrocycle and small anions in methanol (Figure 1). The cycHC[6] is poorly soluble in protonic media; nevertheless, stable solutions with concentrations suitable for ^1^H NMR measurements (ca 1 mM) can be reached by heating the sample combined with sonification. Hence, we prepared samples of cycHC[6] in MeOD-*d*_*4*_ (0.8 mM) and added an excess of Cl^−^ and Br^−^ salts. ^1^H NMR spectra were measured shortly before and after the addition of salts and then 18 h later to rule out a possible slow kinetics of binding (Supplementary Figure S1). Surprisingly, there was no change in the cycHC[6] spectra. Later, we conducted the same experiment in chloroform with the chloride anion, and there was also no visible interaction in this solvent (Supplementary Figure S2). Therefore, we can conclude that in chloroform and even in methanol solutions, the binding of anions (Cl^−^and Br^−^) inside the cycHC[6] cavity is too weak to be observed at the conditions used due to the competition of anion solvation.

### Screening of Various Guests for CycHC[8]

**FIGURE 2:**
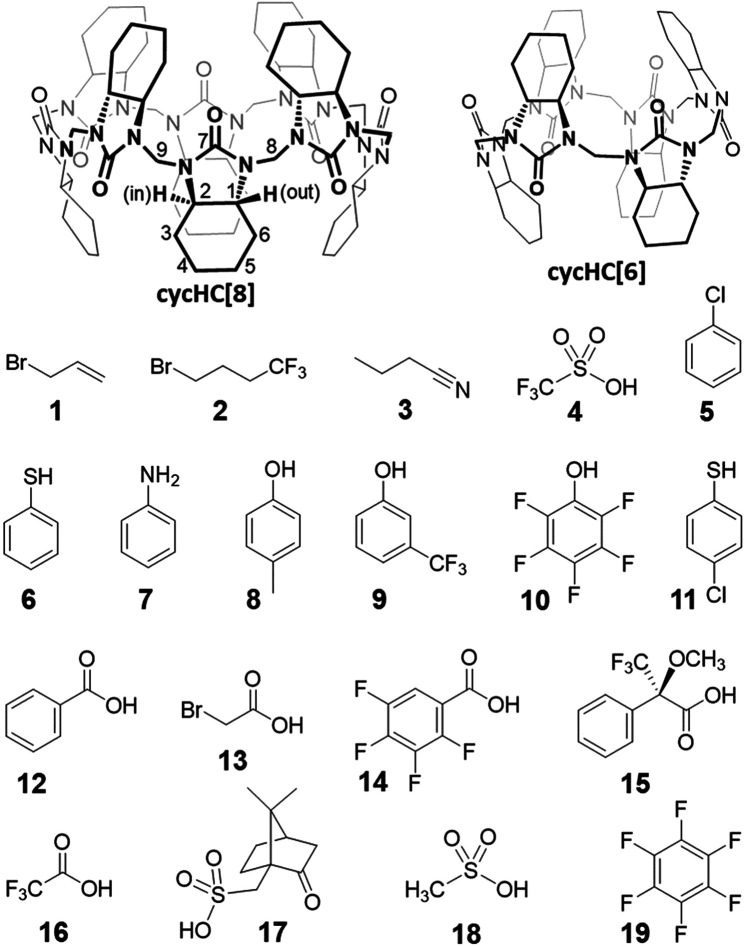
Structures of macrocyclic hosts and small guest molecules used in the study.

Our knowledge of the binding properties of cycHC[*n*]s in aprotic solvents was limited mainly to carboxylic acid derivatives; therefore, several small compounds **1–9** (Figure 2) bearing various functional groups were used to test the interaction with cycHC[8] in chloroform to learn more about the possibility of external and inclusion complex formation. The binding site can be detected by changes in either the spectra of protons positioned inside the cavity or external ones (see the numeration in Figure 2). It was previously shown in methanol that changes of cycHC[8] chemical shifts (*δ*) in ^1^H NMR are most pronounced for the protons H2*ax* and H6*ax* directing inside the cavity and indicating the position of the complexed anion (Kaabel et al., 2017). Similarly, for a complex between cycHC[6] and externally bound trifluoroacetic acid in chloroform, the only slightly shifted signal is H1*ax* directing outside the cavity (Prigorchenko et al., 2019). Interestingly, the binding of porphyrins in dichloromethane has induced a noticeable upfield shift of all macrocycle signals (cycHC[6] or cycHC[8]) as a consequence of the proximity of the large porphyrin’s aromatic system (Ustrnul et al., 2019).

Based on the previous results, we followed ^1^H NMR signals of cycHC[8] (2.5 mM) in the presence of 0, 0.5, 5, and 40 equiv of guests **1–9** in CDCl_3_. Aliphatic guests **1–3** bearing electron-rich groups have not induced any shift of cycHC[8] signals, clearly showing they are not forming any type of complexes (Supplementary Figure S3). The presence of trifluoromethanesulfonic superacid **4** (Howells and Mc Cown, 1977) was expected to provide a hydrogen bond (HB) from its strongly polarized O-H group or cause protonation of the macrocycle. Also, it is known that some superacids are capable of inducing the formation of HCl and chloronium cations from chlorinated solvents (Stoyanov and Stoyanova, 2018). However, the chemical shifts of the macrocycle were not influenced, and we can only speculate that acid four preferred to interact with itself and be passive toward the macrocycle in this solvent.

**FIGURE 3:**
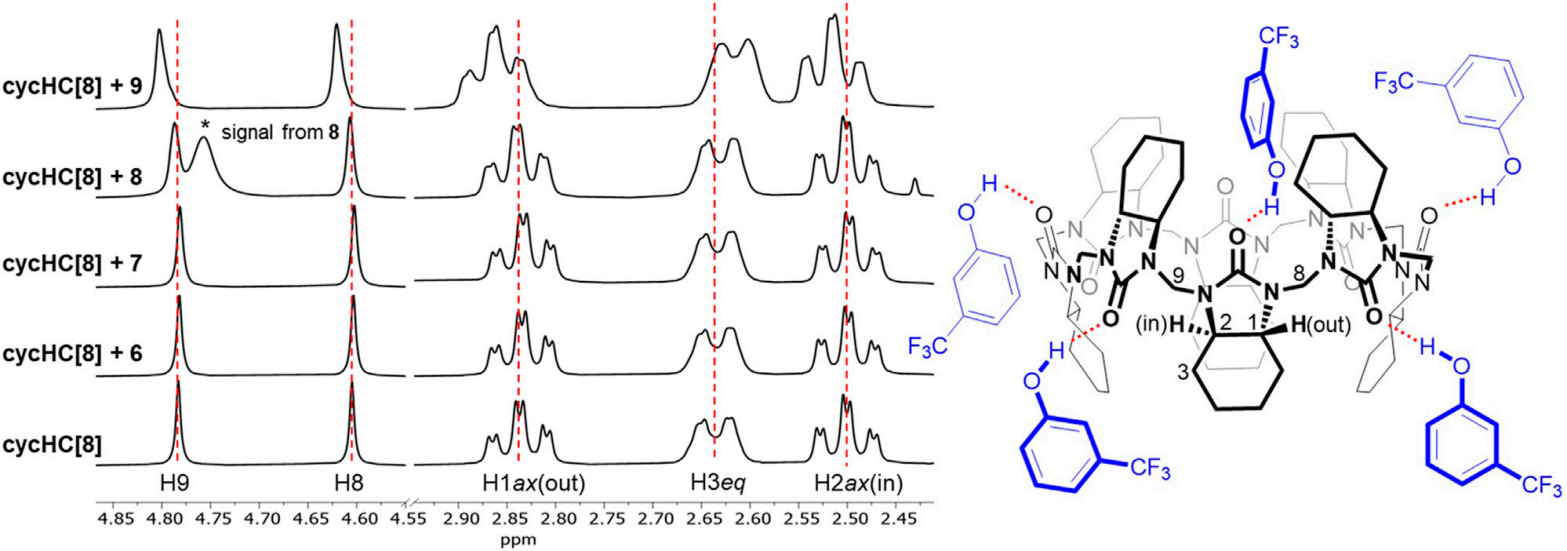
^1^H NMR spectra of free cycHC[8] (2 mM) and in the presence of 40 equiv of guests **6–9** **(left)** and visualization of probable interactions (red, dashed) between **9** and cycHC[8] **(right)**. Guest orientations and binding stoichiometry are illustrative.

Benzene derivatives **5–9** are small enough to possibly fit inside the cycHC[8] cavity (Prigorchenko et al., 2015), so we presumed an inclusion complex for the electron-rich chlorobenzene **5**; nevertheless, no signal shift occurred. The addition of 40 equiv of thiophenol **6** and aniline **7** provided a barely noticeable change in the macrocycle’s proton spectra (Figure 3); hence, we could not deduce the binding site, and we concluded that the compounds were bound very weakly or did not bound at all. Finally, phenol derivatives 4-methylphenol **8** and 3-(trifluoromethyl)phenol **9** induced a change in cycHC[8] chemical shifts (Figure 3). The less polarized **8** caused almost negligible changes, and the more polarized electron-withdrawing group (-CF_3_)-bearing **9** generated a clear shift of multiple signals. A similar change in the chemical shift of cycHC[8]’s inner H2*ax* and outer H1*ax* and methylene bridges H8 and H9 is inconsistent with inclusion complex formation. Alternatively, it can be related to the creation of an external hydrogen bond between the phenol O–H and the carbonyl groups of the macrocycle or to the protonation of the macrocycle formation of an ion pair. The more pronounced change of chemical shifts of signals of guest **9** (more-polarized) compared with guest **8** (less-polarized) support both options. Evidence supporting the hydrogen bond formation was noticed while comparing the chemical shifts of cycHC[8] in the presence of phenol **8** and thiophenol **6**, as, in general, phenols are less acidic than thiophenols (pKa = 18 and pKa = 10, respectively, in DMSO) (Bordwell, 1988). If the protonation of the macrocycle were the main cause of the signal shift, then the thiophenol **6** should induce a larger chemical shift due to the more pronounced protonation. However, this was not the case (Figure 3). Direct evidence for HB formation was obtained by the addition of cycHC[8] to the solution of **10**, which induced a typical strong downfield shift (Hibbert et al., 1990) of the acidic proton in ^1^H NMR of **10** (see Supplementary Figure S4).

In conclusion, guests **1–8** have no or a very weak interaction with cycHC[8], and only the polarized, HB-donating guest **9** induced evident changes in the ^1^H NMR spectra of the macrocycle in chloroform. We assume that a test of binding to cycHC[6] would provide similar results as there was no sign of inclusion complex formation with cycHC[8].

### NMR Titration Studies

Based on the previous screening, hydrogen bonds are the dominant interaction of cycHC[*n*] in chlorinated solvents—the macrocycle’s carbonyl groups act as an acceptor and the guest as an HB donor. It could be deduced that cycHC[6] and cycHC[8] have six and eight available binding sites, respectively. Therefore, a complete description of such a complex system would require a determination of six or eight stepwise association constants. Considering that the necessary amount of reliable experimental data and the subsequent data evaluation would be disproportionately demanding for such a study, we decided to compare the affinity of guests **6** and **9–18** toward cycHC[*n*] by a qualitative approach as described further. Nevertheless, we first needed to scrutinize the general binding properties of the suggested host–guest systems and their cooperativity (Ercolani, 2003; Hunter and Anderson, 2009; Thordarson, 2011).

The macrocycle is relatively rigid at a normal temperature, so we can assume there is no positive or negative cooperativity in stepwise binding caused by its conformational changes (Figure 3). Next, the formation of an HB will cause a local decrease of electron density on the bound carbonyl group, which can induce a small decrease in electron density on the neighboring carbonyl groups, leading to their smaller affinity toward a guest and, therefore, negative cooperativity. Further, we need to consider the role of the guests. All of them bear only one apparent group capable of providing an HB. Hence, we can assume the mechanism of binding to the macrocycle has to be similar. Additional guest-specific effects that influence the binding strength could be 1) guest self-aggregation through the hydrogen bond (e.g., dimerization of carboxylic acids) (Fujii et al., 1988; Colominas et al., 1998) as a competitive interaction to complexation with cycHC[*n*] and 2) steric hindrance between a bulky guest and cycHC[*n*] (see Figure 3) or between guests bound at adjacent carbonyl groups of cycHC[*n*] (significant in saturated complexes). Based on the given reasoning, we can expect no cooperativity or negative cooperativity in the external binding of cycHC[*n*] with single HB donors in a nonpolar solvent. Therefore, every stepwise association constant *K*_*i*_ has to be stronger than the following stepwise *K*_*i+1*_ (for more details, see supporting information on page S9).

**FIGURE 4:**
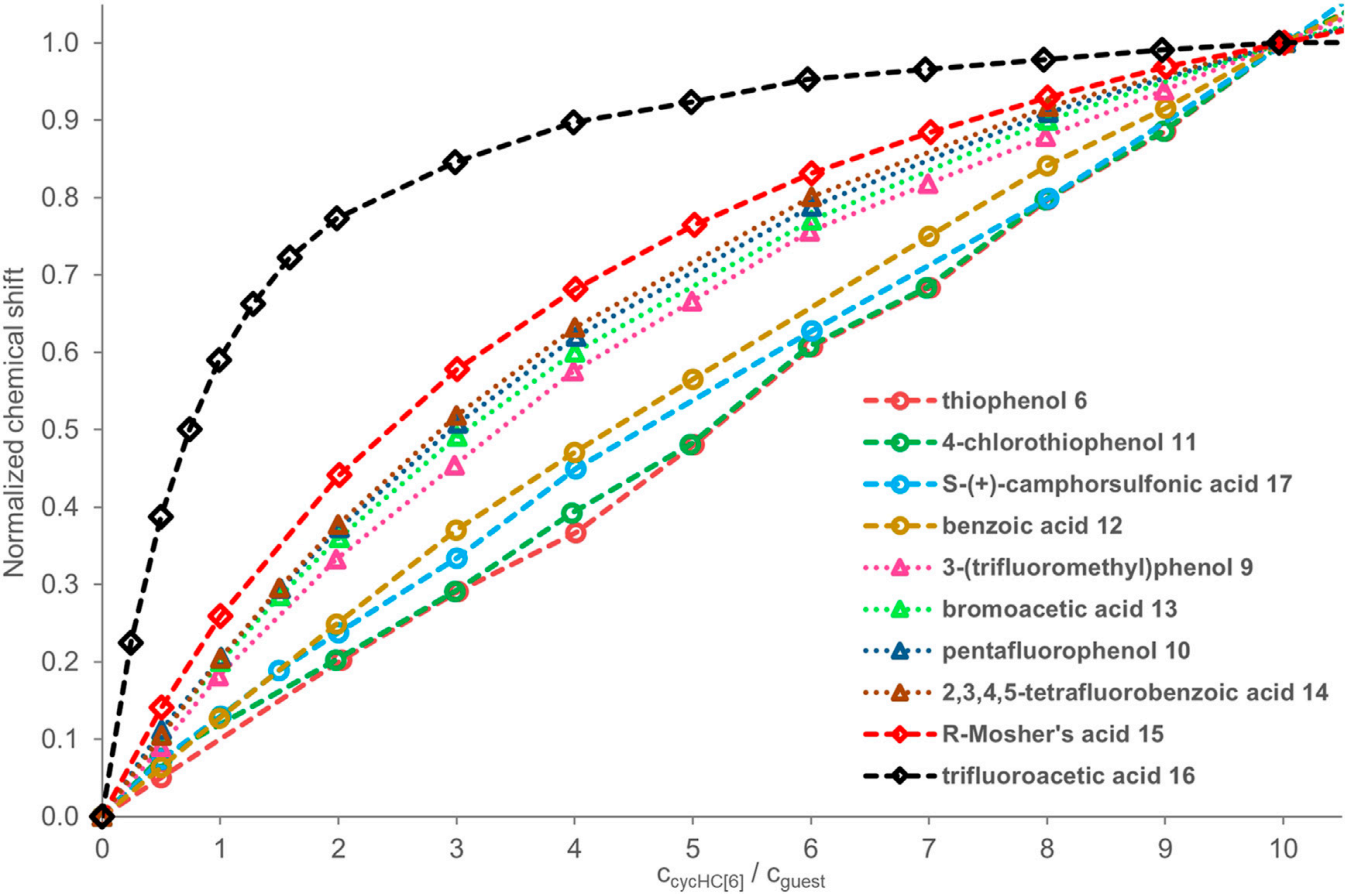
^1^H and ^19^F NMR titration data for guests **6** and **9–17** (2 mM) in the presence of a growing concentration of cycHC[6]; values of guest’s chemical shift *δ* normalized at 10 equiv of cycHC[6]. The legend of the graph follows the order of binding from the weakest **(top)** to strongest **(bottom)**. Experimental data points are assigned with geometrical shapes; the dotted lines are shown to guide the eye. The invalid points were excluded for some guests, specifically for guests **6, 11** (at 1 equiv), and **12** (at 6 equiv). As an internal reference for the fluorine signal position in ^19^F NMR, we added hexafluorobenzene **19**; however, it appeared that the presence of reference inside the samples was not necessary, as we did not observe fluctuations in reference *δ* between individual spectra.

All NMR titrations (^1^H and ^19^F) for a qualitative comparison of binding strength were carried out in the same constant concentration of a guest (2 mM) with additions of specific equivalents of cycHC[6] (Figure 4). The reasons for such experimental setup were as follows: 1) in the previous screening, the ^1^H signals of cycHC[*n*] were relatively insensitive upon interaction with a guest, so we wanted to track the guest’s signal instead; 2) polar and acidic guests could exhibit changes in NMR spectra related to a change of their concentration in chloroform (e.g., dimerization of carboxylic acids); therefore, it is desired to keep their concentration invariable throughout the titration; 3) titration will lead to the excess of cycHC[6], and the formation of a saturated 1:1 complex; and 4) the smaller cycHC[*n*] derivative was chosen for those experiments to prevent any theoretical influence of incorporation of guests into the macrocycle cavity. In this setup, the observed changes in the chemical shift of guests can be associated exclusively with cycHC[6]. Stepwise association constants for the binding of second, third, and further guests will influence the shape of the titration curve only at the very beginning as it is the only condition where an excess of guest over the cycHC[6] is present. When the mechanism of complexation is the same for all the guests, then the more pronounced titration curve of a particular guest (reaching a plateau at lower equivalents of cycHC[6]**)** must correspond with its stronger *K*_*1*_ compared to a guest with a flatter titration curve (reaching a plateau in higher equivalents of cycHC[6]) (Figure 4). Moreover, the strength of *K*_*1*_ should be representative in this case and sufficient to compare the overall binding affinity of different guests as we cannot determine cumulative association constant *β* (*β* = *K*_*1*_ · *K*_*2*_ · ... · *K*_*m*_, where *m* corresponds to the stoichiometry of complex), due to the limitations of acquiring reliable data for higher stoichiometry (see supporting information, page S10).

On that account and all mentioned above, we can compare the strength of binding of selected guests with each other by simply comparing the shapes of titration curves. To do that, we normalized the experimental values from NMR as the extent of chemical shift *δ* change varies for different guests and depends on their sensitivity to the change of their close electronic environment upon binding. Data were normalized at 10 equiv of cycHC[6] (see details in SI) as such an excess of macrocycle should be sufficient to get over 85% saturation of 1:1 complex even for moderate association constants (simulated for *K*_*1*_ = 1000 M^−1^, *K*_*2*_ = 500 M^−1^ with a 2:1 NMR binding model in the online tool Bindsim from supramolecular.org) (Thordarson, 2011; Hibbert and Thordarson, 2016).

**FIGURE 5:**
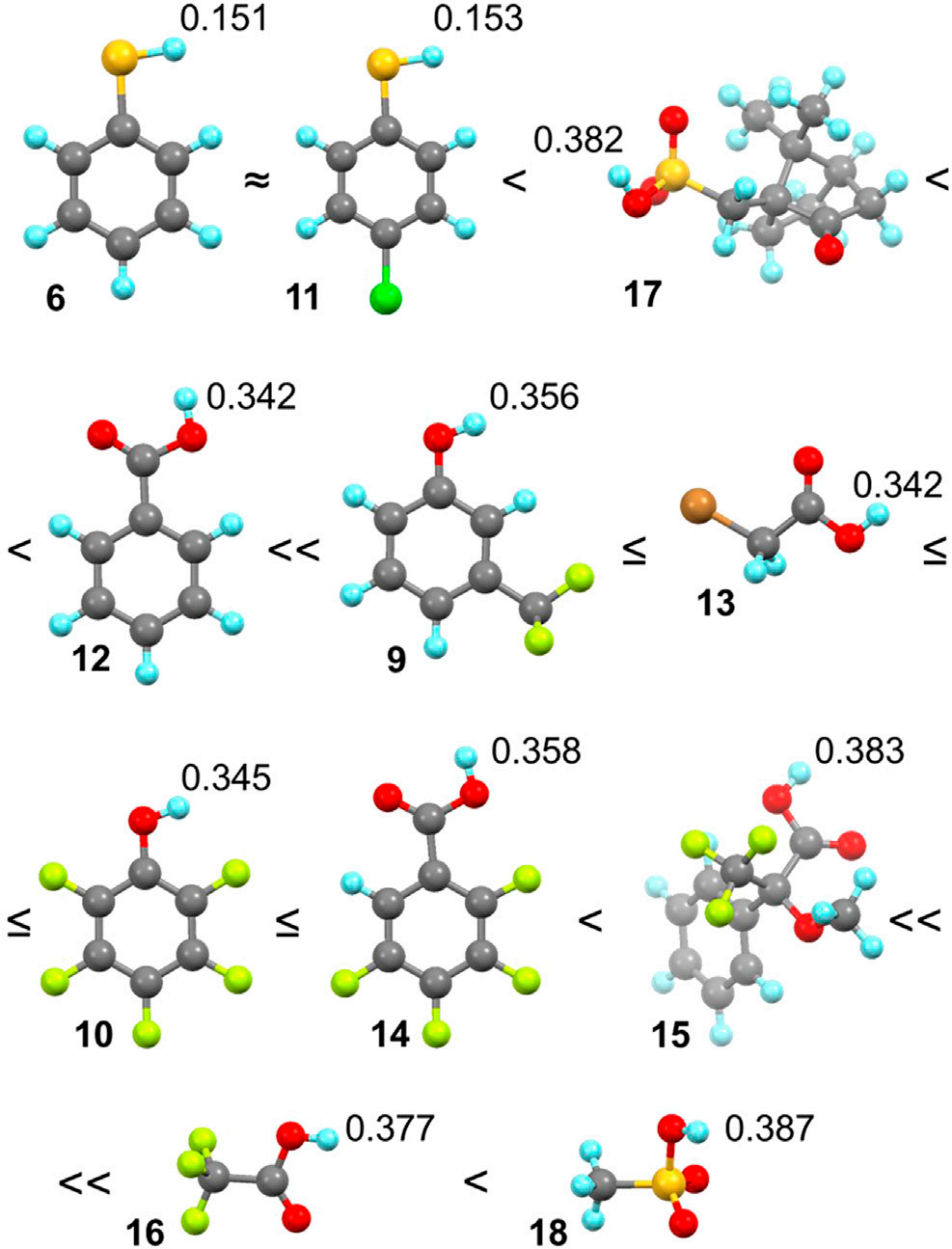
Order of binding according to the shape of titration curves (assigned by ≈, ≤, <) and values of partial positive charge on the hydrogen atom participating in the hydrogen bond formation of guests **6** and **9–18** with cycHC[6]. Color coding: H: bright blue, C: dark gray, O: red, S: yellow, F: bright green, Cl: green, and Br: orange.

We aimed to observe differences between various HB-donating functional groups and the influence of electron-withdrawing groups in the structure of guests as they should enhance the strength of the HB. The selected guests **6** and **9–18** fulfilled those aims well. We expected that the binding of HB donors with cycHC[6] should correlate with Abraham’s hydrogen bond acidity (Abraham, 1993; Abraham et al., 2006), which is a measure of the compound’s ability to perform as an HB donor or acceptor. However, the values are not available for all studied guests, and we could only generalize that in our set of guests, the thiophenols are bad HB donors, and phenols with carboxylic acids should be roughly equal. However, we were interested in comparing the whole set of measured guests. Therefore, we calculated Hirshfeld partial atomic charges for protons contributing to HB interaction with cycHC[6] (Figure 5) using the density functional theory (DFT). The higher value means a higher partial positive charge, therefore, a better ability to act as an HB donor. One should bear in mind that gas-phase calculations explain the relative polarity difference between the studied guests and cannot fully reflect the situation in the solution.

In the qualitative comparison of binding (Figure 4), the titrations of thiophenols **6** and **11** provided barely any signal shift, which resulted in a straight line instead of a curved titration isotherm. This observation is not surprising as sulfur has a significantly lower electronegativity, and our calculations showed a very small partial charge on a hydrogen atom compared to the other guests. Surprising results were obtained for camphorsulfonic acid **17**; its acidic proton had a larger atomic charge than that of carboxylic acids, but the titration curve of **17** was very flat, suggesting binding barely stronger than that with thiophenols **6** and **11**. Reasons for such a weak interaction can be 1) steric hindrance between the macrocycle and the bulky structure of **17**, and 2) formation of intra- or intermolecular HB between the sulfonic group and the carbonyl group of **17**.

Guest **12** provided a less pronounced titration curve than equally charged **13** and **10**, which could be related to a steric hindrance and eventually to the different behavior of different functional groups in the solution. Stronger than **12** was a group of guests with similar titration curves showing the order of binding corresponding well with atomic charges (**9** ≤ **13** ≤ **10** ≤ **14**) with small irregularity. The binding of the phenol derivative **9** is slightly weaker than that of **10**, which is in the opposite order of their partial charge on the proton. However, this could also be reasoned by the steric hindrance of the trifluoromethyl group. Like **12**, **15** has a weaker affinity in comparison with equally charged but less sterically hindered **16** and **18**. Trifluoroacetic acid **16** is a small molecule bearing an electron-withdrawing CF_3_ group nearest to the HB donor, causing its high atomic charge and the strongest interaction with cycHC[6].

Methanesulfonic acid **18** exhibited results that did not correspond between parallel titrations due to difficulties in the sample preparation caused by the low solubility of **18** in chloroform. Accurate experimental concentrations of **18** in the titrations were obtained from comparing the NMR signal intensities of **18** and a compound of known concentration, which was either host cycHC[6] or, on one occasion, a standard 1,2,4,5-tetrachloro-3-nitrobenzene. Unfortunately, we could not include guest **18** in Figure 4 because its concentrations in titrations were not comparable with other guests (titration data for all guests, including concentrations other than 2 mM, are present in Supplementary Tables S1–S22). At the same time, we were able to estimate that the binding of **18** with cycHC[6] is the strongest out of all the tested guests because at the concentration ca of 0.4 mM, the titration curve was as pronounced as the curve of 2 mM trifluoroacetic acid **16** (see Supplementary Figure S8).

### Enantioselective Interaction With Chiral Guests

**FIGURE 6:**
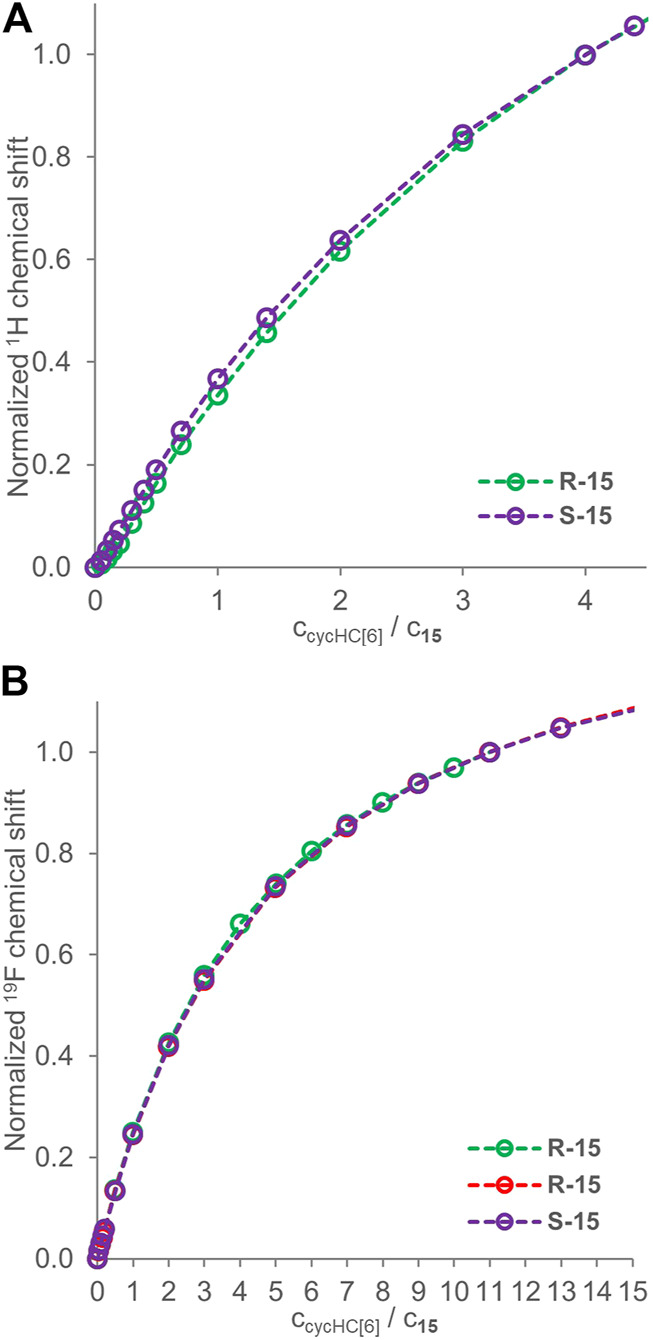
Titration data for enantiomers of guest **15** (2 mM) in the presence of a growing concentration of cycHC[6]. Graph **(A)** shows ^1^H NMR normalized at 4 equiv, and **(B)** shows ^19^F NMR normalized at 11 equiv. Experimental data points are assigned with circles; the dotted lines are shown to guide the eye.

Previous studies showed chiral recognition of *α*-methoxyphenylacetic acid enantiomers by cycHC[6], illustrated by changes in ^13^C NMR (Aav et al., 2013). Those studies also showed enantioselective binding by cycHC[6] and cycHC[8] based on the DOSY NMR and fitting with a 1:1 model of binding (Prigorchenko et al., 2015). Further, a low selectivity of binding for Mosher’s acid (guest **15**) enantiomers by cycHC[8] was reported using the same method (DOSY NMR). However, the ability of cycHC[6] to differentiate between enantiomers of **15** was not tested, so we decided to apply our qualitative approach to this system. First, we conducted the ^1^H NMR titration for both enantiomers of **15** (2 mM) (see Supplementary Table S11, S12). The change in chemical shift was very small, and normalized titration curves (at 4 equiv) were almost overlapping (Figure 6A). Then we exploited ^19^F NMR in two titrations of *R*-**15** and one titration of *S*-**15** (2 mM) and normalized the titration curves at 11 equiv (see Supplementary Tables S8–S10) as the latest common data point (Figure 6B). Surprisingly, the titration curves overlap perfectly, so it seems cycHC[6] cannot bind Mosher’s acid **15** enantioselectively, or the selectivity is so low that it cannot be differentiated by the method used. Indeed, only a very low selectivity was previously reported for cycHC[8] (Prigorchenko et al., 2015).

### Evaluation of Apparent Association Constants

The evaluated system should have negative or no cooperativity. Therefore, *K*_*a*_ values for higher stoichiometry complexes are decreasing, and the concentration of corresponding complexes becomes negligible in solution. Additionally, there is generally no meaning in fitting data with the model using higher stoichiometry if such fitting does not significantly improve the overall quality of fit (Thordarson, 2011; Hibbert and Thordarson, 2016). Therefore, we decided to apply an adapted version of our 3:1 binding model, developed to evaluate the porphyrin binding (Ustrnul et al., 2019), to quantify the binding between the HB donors and cycHC[*n*].

The fitting with the 3:1 binding model for titration data used at a qualitative comparison of binding strength has revealed that most of the studied guests had too low binding for acceptable determination of the first three apparent stepwise *K*_*obs*_; therefore, 2:1 and 1:1 binding models had to be used. Only some of the titration data for guests **16** and **18** provided a reasonable fit for *K*_*1obs*_–*K*_*3obs*_ (Supplementary Table S23). Overall, the binding between the guests and cycHC[6] cannot be compared quantitatively as the data cannot be evaluated with the same binding model (see supporting information, page S32–S33). Nevertheless, we focused on the strongest binding acids **16** and **18** to confirm the stoichiometry of the complexes higher than 2:1 and evaluated *K*_*obs*_ with a 3:1 (Python script) and a 2:1 (Bindfit) binding model.

**FIGURE 7:**
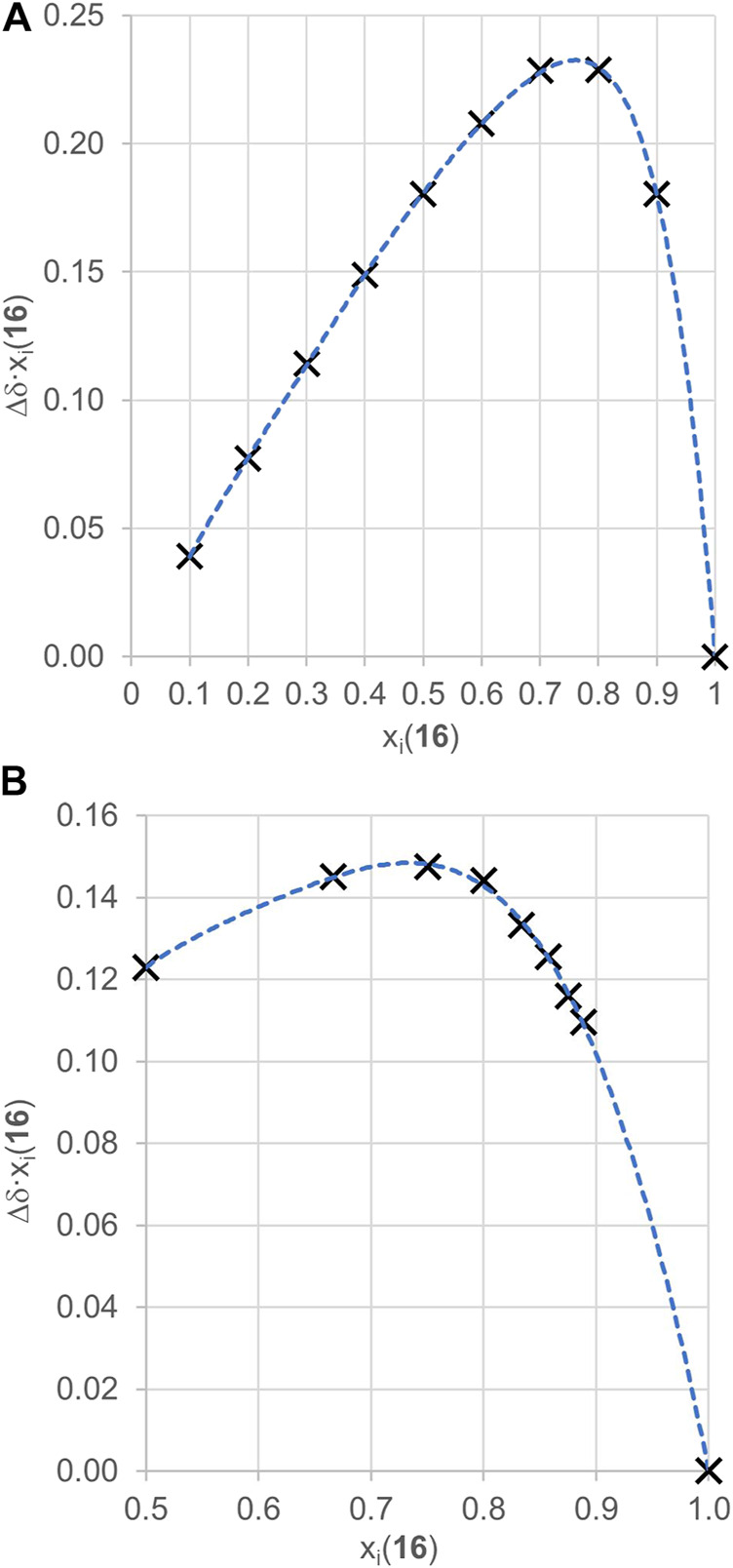
^19^F NMR Job plot experiment results for a complex of **(A)** cycHC[8] with guest **16** (c_total_ = 10 mM) and **(B)** cycHC[6] with guest **16** (c_total_ = 20 mM). Black crosses are experimental points, and the blue dotted lines are shown to guide the eye.

To justify using the 3:1 binding model, we have conducted a continuous variation method known as a Job plot experiment between guest **16** and cycHC[6] and also cycHC[8] by ^19^F NMR. Although it was previously proved that the Job plot method is not suitable for determination of precise stoichiometry, it can still provide useful and demonstrative information for suitable systems (Hibbert and Thordarson, 2016; Ulatowski et al., 2016). First, we conducted the experiment with cycHC[8] (Figure 7A) using a common arrangement, varying mole fractions of host and guest from 0 to 1 at a high overall concentration (10 mM). Results showed a maximum at points 0.7 and 0.8 mole fraction of guest **16** (full data in Supplementary Table S25); therefore, we can assume that the maximum of the curve lay around 0.75, which is a position corresponding to the formation of a complex with a stoichiometry at least 3:1 (**16**:cycHC[8]). Based on those results, we modified the experimental arrangement for measurement with cycHC[6] (Figure 7B) and increased the concentration (20 mM). Also, we focused on mole fractions in excess of guest **16**. The mole fraction values were chosen to correspond with specific host–guest ratios (x_i_ = 0.5 for 1:1, x_i_ = 0.66 for 2:1, x_i_ = 0.75 for 3:1, x_i_ = 0.8 for 4:1,...). Obtained data revealed a flat top between 0.66 and 0.8 with a maximum at 0.75 (full data in Supplementary Table S26), which reflects at least 3:1 binding (**16**:cycHC[6]) stoichiometry, the same as cycHC[8]. Hence, using the 3:1 binding model to evaluate apparent association constants between **16** and cycHC[*n*] should provide values of *K*_*obs*_ that better correspond to reality than models describing a lower stoichiometry binding.

**FIGURE 8:**
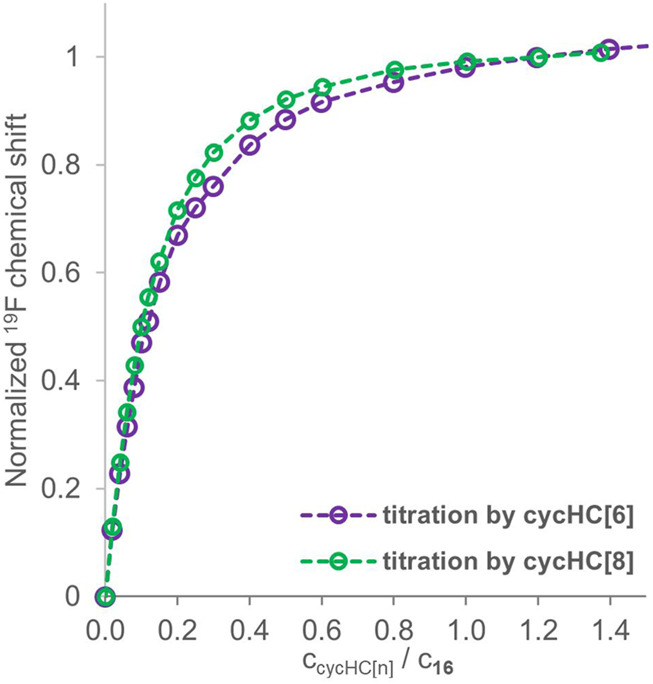
^19^F NMR titration data for guest **16** (18 mM) in the presence of a growing concentration of cycHC[6] (purple) and cycHC[8] (green) normalized at 1.2 equiv. Experimental data points are assigned with circles; the dotted lines are shown to guide the eye.

We have conducted additional ^19^F NMR titrations to collect sufficient data to evaluate association constants between trifluoroacetic acid **16** and cycHC[*n*] macrocycles. Specifically, we have measured two titrations with cycHC[8] at the same concentration of guest **16** (2 mM) as in the titrations for a qualitative comparison. Then, we performed two titrations at a higher concentration of **16** (18 mM), one with cycHC[6] and one with cycHC[8] (full titration data in Supplementary Tables S13–S17), because concentration range broadening of titrations should improve the outcome quality of the following fitting procedure more than simply repeating experiments at the same concentration (Thordarson, 2011; Hibbert and Thordarson, 2016). In the end, we had data for 2 mM and 18 mM solutions of guest **16** with studied cycHC[*n*]s that allowed us to compare cycHC[6] and cycHC[8] using our qualitative approach. Titration data for 18 mM guest **16** were normalized at 1.2 equiv and provided almost identical titration curves for both cycHC[*n*] (Figure 8). However, a slightly more pronounced curve for titrations with cycHC[8] suggests a stronger binding, probably as a result of a larger amount of available binding sites (carbonyl groups) on cycHC[8] than cycHC[6]. Data collected for 2 mM guest **16** and normalized at 5 equiv confirmed the trend observed at the higher concentration (see Supplementary Figure S5). Nevertheless, such comparison is only demonstrative as the binding mechanism is not known in detail and can differ due to the different size or properties of cycHC[6] and cycHC[8].

**FIGURE 9:**
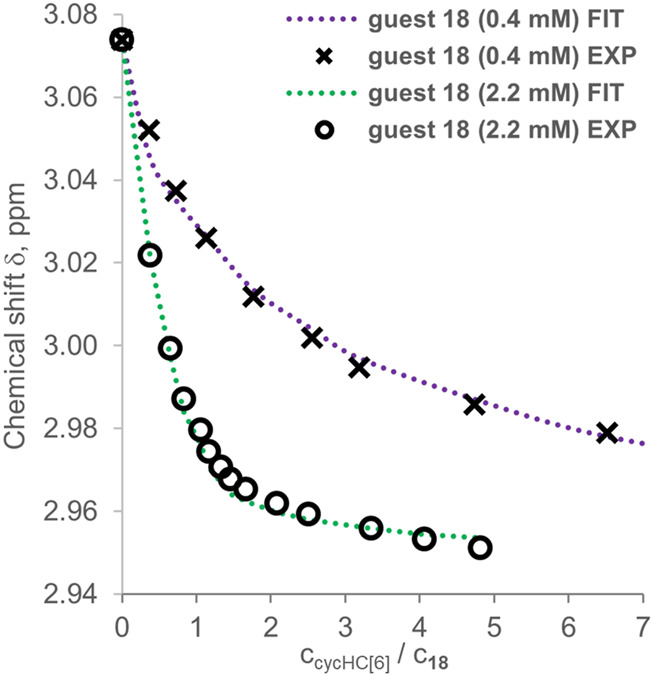
Experimental points and binding isotherms (dotted lines) obtained from simultaneous fitting for titrations of guest **18** titrated by cycHC[6], which were performed at different concentrations.

We attempted to determine *K*_*obs*_ by a 3:1 (Python script) and a 2:1 (Bindfit) binding model from all the titration data collected for both guests **16** and **18** with cycHC[*n*] (Supplementary Table S23). The 3:1 binding model allowed us to evaluate the data from titrations at different concentrations simultaneously; however, it was successfully realized only for two datasets for **18** titrated by cycHC[6] (Table 1, entry 1). The experimental data and obtained fit (Figure 9, the normalized version in Supplementary Figure S9) show a more pronounced titration curve at a higher concentration of **18** due to the increased abundance of the 1:1 complex and higher stoichiometry complexes. By combining data from various concentrations, the simultaneous fit should provide more precise *K*_*obs*_; however, that cannot apply to systems with *K*_*obs*_ significantly dependent on experimental concentration. Therefore, we can assume that simultaneous fit in most cases failed because of the dimerization of acids in chloroform. Hence, we applied the 3:1 model on every titration dataset separately and obtained a reasonable fit between experimental and calculated values only for one experiment for every combination of acid and cycHC[*n*] (Table 1), giving us no opportunity to evaluate the reliability of the results. Fitting for the guest **18** with cycHC[8] (Table 1, entry 2) showed *K*_*1obs*_ ≈ *K*_*2obs*_ values corresponding to a positive cooperativity, which we ruled out earlier. Guest **16** with cycHC[6] exhibited an error of *K*_*3obs*_ same as the value itself (Table 1, entry 3 and Supplementary Figure S10). Finally, **16** with cycHC[8] showed reasonable values of all three *K*_*obs*_ (Table 1, entry 4).

**TABLE 1:** Observed association constants (*K*_*obs*_) obtained from fitting with a 3:1 binding model.

	Guest	Macrocycle	Guest concentration, mM	*K*_*1obs*_, M^−1^	*K*_*2obs*_, M^−1^	*K*_*3obs*_, M^−1^
1.^a^	**18**	**cycHC[6]**	0.4	1,700 ± 250	500 ± 250	400 ± 200
	**18**	**cycHC[6]**	2.2			
2.	**18**	**cycHC[8]**	0.8	1,211 ± 150	1,175 ± 100	470 ± 120
3.^b^	**16**	**cycHC[6]**	2.0	600 ± 100	350 ± 50	30 ± 30
4.	**16**	**cycHC[8]**	1.9	1,180 ± 20	326 ± 17	195 ± 20

a Experimental data from two independent titrations were fitted simultaneously.

b Despite the large deviation of K_3obs_, the overall fit is reliable; see the distribution of residuals in Supplementary Figure S10.

Interestingly, we were not able to determine *K*_*obs*_ for any titration of 18 mM **16**. All these results indicate that the 3:1 binding model cannot provide reliable *K*_*obs*_ without a better understanding of the binding mechanism as it would lead to additional or improved constraints. Moreover, the binding at higher concentrations of acid could still be difficult to evaluate due to an increasing influence of complexes with stoichiometry over 3:1.

**FIGURE 10:**
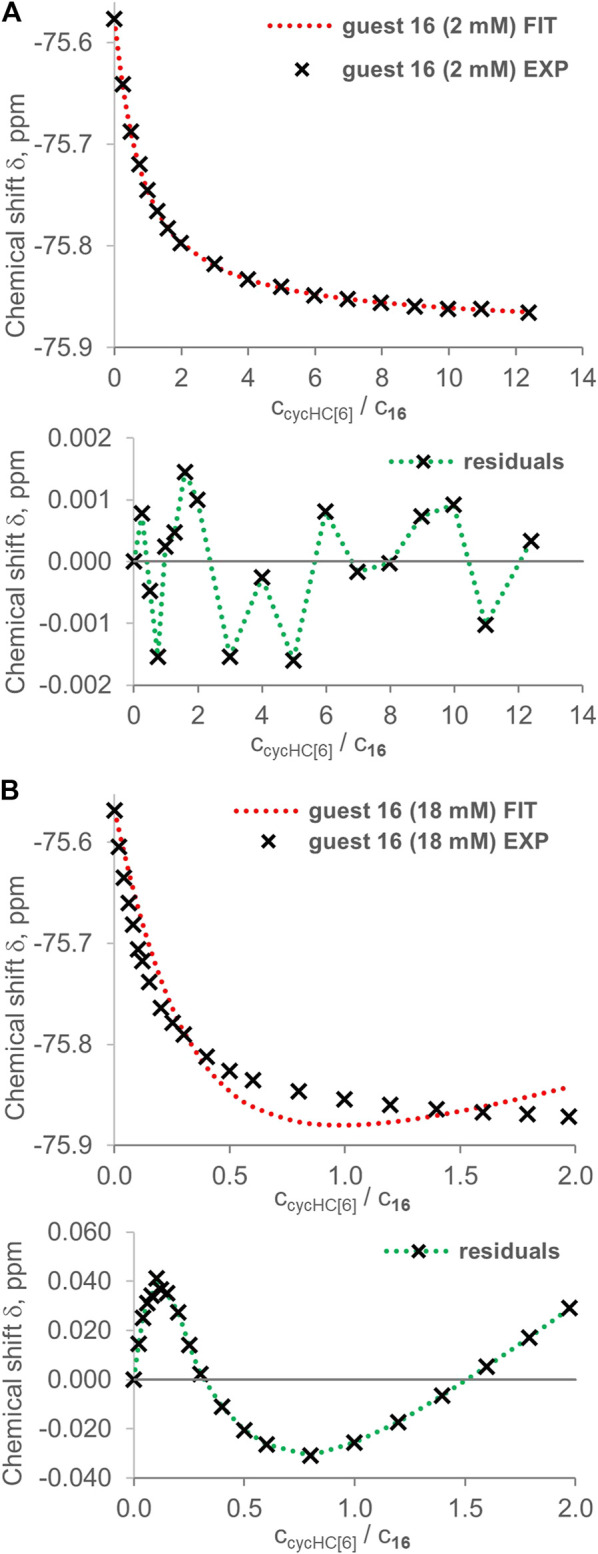
Examples of fitting by the online tool Bindfit (supramolecular.org) using a 2:1 binding model for guest **16** titrated by cycHC[6] at different concentrations. Upper graphs show experimental points (black crosses) and fitted binding isotherm (red dashed line); lower graphs show residuals (distance between the experimental value and fitted isotherm). In the case of **(A)** 2 mM **16** **,** we obtained a good fit and *K*_*1obs*_ = 928 ± 17 M^−1^ and *K*_*2obs*_ = 501 ± 38 M^−1^. However, for **(B)** 18 mM **16,** we obtained a poor fit reflecting the inappropriate binding model (*K*_*1obs*_ = 0.04 ± 0.01 M^−1^, *K*_*2obs*_ = 228,544 ± 21,727 M^−1^).

Evaluation of titration data by the 2:1 binding model provided additional evidence for the higher stoichiometry of binding. In the case of 2 mM guest **16** titrated by cycHC[6], a very good fit was obtained, as can be recognized from the random distribution of residuals (Figure 10A). The 2:1 binding model seems to suit the host–guest system very well, and we could falsely deduce that, indeed, one cycHC[6] binds exactly two molecules of **16**. However, the determined *K*_*1obs*_ = 928 ± 17 M^−1^ and *K*_*2obs*_ = 501 ± 38 M^−1^ indicate refuted positive cooperativity (*K*_*2obs*_/*K*_*1obs*_ > 5/12 for 6:1 systems or *K*_*2obs*_/*K*_*1obs*_ > 1/4 for 2:1 systems; for more details, see supporting information, page S9). In addition, both *K*_*obs*_ are significantly different from the previously published *K*_*1obs*_ = 280 ± 10 M^−1^, *K*_*2obs*_ = 630 ± 20 M^−1^ obtained for similar guest concentration (Prigorchenko et al., 2019).

If any of these apparent *K*_*1obs*_ and *K*_*2obs*_ were real association constants, then an evaluation of titration for 18 mM guest **16** should provide similar values of binding constants or possibly lower values due to enhanced competition with the guest's dimerization. Nevertheless, the fitting of 18 mM **16** titrated by cycHC[6] provided large sinusoidal residuals, and we can speculate that the large *K*_*2obs*_ and a very steep change of experimental values of the chemical shift at low equivalents of cycHC[6] are evidence for the formation of higher stoichiometry complexes (Figure 10B). Comparable results were obtained from the fitting of data for the same acid (**16**) with cycHC[8] (Supplementary Table S23). Two titrations at 2 mM concentration gave, on average, *K*_*1obs*_ = 389 ± 59 M^−1^ and *K*_*2obs*_ = 601 ± 85 M^−1^. An 18 mM titration exhibited large sinusoidal residuals (see Supplementary Figures S11–S13). In the case of acid **18**, concentrations were very similar in all titrations, but the results of fitting were inconsistent. Overall, a comparison of all titration experiments for guests **16** and **18** shows the trend of larger apparent positive cooperativity (*K*_*1obs*_ < *K*_*2obs*_) for experiments at elevated concentrations of guest that can be easily explained by a higher relative amount of complexes with stoichiometry over 2:1 at the beginning of titration in such conditions. It also indicates that a 2:1 binding model is inappropriate as it does not correspond to real complex stoichiometry.

## Conclusion

Previous studies of cycHC[*n*]-binding properties in nonpolar solvents (chloroform) have focused almost exclusively on interactions with carboxylic acids and their derivatives. The DOSY NMR was used to investigate the complex formation at the molar equivalency of the host and guest. It was not suitable for uncovering the existence of complexes of higher stoichiometry. Later, classical NMR titrations were conducted for cycHC[6] derivatives and trifluoroacetic acid. However, data evaluation with a 2:1 binding model that underestimated the stoichiometry of the guest has provided the false impression of positive cooperativity.

This article has broadened the scope of knowledge on cycHC[*n*]-binding properties in chloroform by testing interactions between a set of guests **1–9** bearing various functional groups and cycHC[8]. Only the guests capable of providing a hydrogen bond exhibited signs of complex formation through the carbonyl groups of the macrocycle. Despite a size difference, cycHC[6] is chemically analogous to cycHC[8] and, due to their similar binding properties, the external binding of guests was confirmed.

We carried out titrations for cycHC[6] and polar organic hydrogen bond donors from a group of thiophenols, phenols, carboxylic acids, and sulfonic acids (guests **6** and **9–18**). We observed a broad range of binding strengths from almost no binding (thiophenols) to moderate (acids **16** and **18**), which did not allow the quantification of association constants with the same binding model for all the collected data and their subsequent comparison. Therefore, we have made a qualitative comparison based on titration curves. The final order of binding strength from weakest to strongest was **6** ≈ **11** < **17** < **12** << **9** ≤ **13** ≤ **10** ≤ **14** < **15** << **16** and correlates with the increasing hydrogen bond donor's partial atomic charge. Methanesulfonic acid **18** exhibited strong binding, but it could not be compared to other guests directly due to limited miscibility in chloroform.

We collected additional data to quantify apparent association constants for the strongest binding acids **16** and **18** with our 3:1 and the Bindfit 2:1 binding model. Surprisingly, the 3:1 binding model did not provide a fit for all of the data; however, for acid **18** with cycHC[6], we have obtained *K*_*1obs*_ = 1700 ± 250 M^−1^, *K*_*2obs*_ = 500 ± 250 M^−1^, and *K*_*3obs*_ = 400 ± 200 M^−1^ from simultaneous evaluation of two titrations while the 2:1 model gave inconsistent results for all titrations of **18**. None of the models provided an acceptable fit for measurements at high concentration (18 mM) for acid **16**; this could be a consequence of the formation of higher stoichiometry complexes. Nevertheless, we observed a very good fit from 3:1 and 2:1 binding models for titrations of 2 mM of **16**. In such cases, the simpler model should be preferred; therefore, we can report *K*_*1obs*_ = 928 ± 17 M^−1^ and *K*_*2obs*_ = 501 ± 38 M^−1^ with cycHC[6] and *K*_*1obs*_ = 389 ± 59 M^−1^ and *K*_*2obs*_ = 601 ± 85 M^−1^ with cycHC[8]. However, macrocycles have enough binding sites to accommodate more than two or three guests, so the determined stepwise association constants are only apparent. We can assume that they include information on higher stoichiometry *K*_*obs*_ because of our experimental arrangement. We consider the determined *K*_*obs*_ to be valid and useful for the same (ca 2 mM) or lower concentrations in chloroform, which we used in corresponding titration experiments.

Unlike other single-bridged cucurbituril family members, the current study confirmed that the binding of chloride and bromide with cycHC[6] does not occur in methanol and chloroform solutions.

This article has provided new insights into the cycHC[*n*] binding properties that will help recognize promising guests from several biologically active compounds. The binding of HB-donating guests is a dominant interaction in chlorinated solvents, and its strength can be related to the shape, size, and partial charge on the hydrogen atom (of the HB-donating functional group) of the guests. The evaluation of stepwise association constants is challenging because cycHC[*n*] can bind three or more guests at once. Hence, we have demonstrated an alternative approach of qualitative comparison of binding affinity for a set of guests. The same method can be used for any host–guest system where the same binding mechanism can be assumed for all tested guests.

## Data Availability

The original contributions presented in the study are included in the article/Supplementary Material, and further inquiries can be directed to the corresponding authors.
