# Supplementary material for: Binding Between Cyclohexanohemicucurbit[n]urils and Polar Organic Guests
Source: Front Chem. 2021 Jun 28;9:701028. doi: 10.3389/fchem.2021.701028 (PMC8273652; doi:10.3389/fchem.2021.701028)
Supplement: Supplementary file 1 [file DataSheet1.pdf]

Supplementary Material

Table of content

1 General information about materials, methods and instrumentation 2
1.1 Materials, reagents and solvents 2
1.2 Sample preparation 2
1.3 NMR measurements 2
1.4 Anion binding with cycHC[6] 2
1.5 Screening of potential guests in chloroform 2
1.6 Test of hydrogen bond formation 2
1.7 Titration experiments in chloroform 3
1.8 DFT calculations of partial atomic charges 3
1.9 Job's plot experiments 3
1.10 Binding strength evaluation 3
2 Structures of hosts and guest used in the study 4
3 Anion binding with cycHC[6] 5
4 Screening of potential guests (1-9) in chloroform 7
5 NMR titrations, Job plots and the data evaluation 9
5.1 Cooperativity evaluation criterions for cycHC[*n*] 9
5.2 Normalization of titration curves 10
5.3 NMR titration data for association constants evaluation 10
5.4 NMR titration data 11
5.5 Evaluation of association constants with 1:1 and 2:1 binding model in Bindfit 32
5.6 Evaluation of association constants with 2:1 and 3:1 binding model 33
5.7 NMR data for continuous variation method (Job plot) 41
6 Results of DFT calculations of partial atomic charges 42
7 References 43

1 General information about materials, methods and instrumentation

1.1 Materials, reagents and solvents

All reagents and solvents were purchased from commercial suppliers. Macrocyclic host compounds were used only as (*R*,*R*)-cycHC[*n*] enantiomers and were synthesized in our laboratory from (*R*,*R*)-cyclohexanourea according to procedures described in literature (Aav et al., 2013; Prigorchenko et al., 2015; Kaabel et al., 2019).

1.2 Sample preparation

All the solutions were prepared using Hamilton® Gastight syringes, those syringes was also used for all the additions during titrations. For the precise measurement of higher volumes (over 1 ml) was used the mass of solvent and its density instead of volumetric glassware. Samples were weighed on a microbalance with an accuracy of 6 μg (Radwag® MYA 11.4Y, Poland).

1.3 NMR measurements

^{1}H NMR (400 MHz) and ^{19}F NMR (376.5 MHz) spectra were recorded on Bruker Avance III spectrometer, using a Bruker BBO probe equipped with a z-gradient coil. Chemical shifts were referenced to residual proton solvent peak ($\delta(^{1}H)$ =3.34 ppm in CD_3OD-d_4 and $\delta(^{1}H)$ =7.26 ppm in $CDCl_3$) or to TMS (0.00 ppm) as internal standard. All chemical shifts are reported in ppm units. The data was analyzed using the program MNova (Mestrelab).

As an internal reference for the fluorine signal position in ^{9}F NMR of **9**, we added hexafluorobenzene **19**; however, it appeared that presence of reference inside the samples was not necessary as we did not observed fluctuations in reference δ between individual spectra.

1.4 Anion binding with cycHC[6]

Binding of anions to **cycHC[6]** was tested in CD_3OD-d_4 (0.8 mM **cycHC[6]**) and $CDCl_3$ (1.2 mM **cycHC[6]**) by addition of salt excess to the macrocycle solution. Tetrabutylammonium (TBA) chloride and bromide have been added as a solid compound. Specific excess of salt was determined from integration of NMR signals against known concentration of macrocycle. ^{1}H NMR was measured shortly before and after a salt addition and then after 18 h. Dissolution of weakly soluble **cycHC[6]** in methanol was achieved by employing repeatedly heating and sonification.

1.5 Screening of potential guests in chloroform

Chemical shifts changes of **cycHC[8]** (ca 2.5 mM) proton signals induced by addition of 0.5, 5 and 40 equiv of guests (**1**-**9**) were investigated by ^{1}H NMR in $CDCl_3$. Guests were added as a solutions of known concentration (typically 300-400 mM).

1.6 Test of hydrogen bond formation

^{1}H NMR of pentafluorophenol (50 mM) in $CDCl_3$ was measured before and after the addition of solid **cycHC[8]** (20 mg), which provided solution containing roughly 0.5 eq of macrocycle.

1.7 Titration experiments in chloroform

All NMR titrations were performed at constant concentration of guest (2 mM for qualitative comparison tirations) in a sample throughout the whole experiment, that was achieved by dissolution of titrant (cycHC[n]) in the solution of guest. ^{1}H or ^{19}F NMR was used according to the guest's structure with a preference to use ^{19}F NMR as it usually provided larger changes in chemical shift.

1.8 DFT calculations of partial atomic charges

The structures of the guests were built using the Avogadro molecular editor (Hanwell et al., 2012) and pre-optimised with the MMFF94 force field (Halgren, 1996). Density functional theory (DFT) with the functional B3LYP-D (D3BJ dispersion model) (Lee et al., 1988; Becke, 1992; Stephens et al., 1994; Ehrlich et al., 2011; Grimme et al., 2011; Vosko et al., 2011) in combination with the 6-311G** basis set (Frisch et al., 1984) was used to re-optimise the structures and the frequency calculations were used to confirm that the found structures were at a minimum. All DFT calculations were performed with NWChem 6.8 program package. (Aprà et al., 2020) The wavefunction from the DFT calculations was analysed by Multiwfn (Lu and Chen, 2012b) and the partial charges were found using the Hirshfield charges model (Hirshfeld, 1977; Lu and Chen, 2012a). Mercury 4.2.0 (Macrae et al., 2006, 2008, 2020) was used to visualize the structures of molecules for Figure 5 in the main text.

1.9 Job's plot experiments

Continuous variation method was conducted at total concentration 10 mM for trifluoroacetic acid (guest **16**) and **cycHC[8]** and at total concentration 20 mM for **16** and **cycHC[6]**. It means that we prepared solutions of a same concentration for both compounds used at particular experiment and then prepared set of NMR samples varying the ratios between the two compounds, therefore the total concentration in every sample is same.

1.10 Binding strength evaluation

Stepwise association constants were evaluated (Bindfit) and simulated (Bindsim) using online tools at supramolecular.org (Thordarson, 2011; Hibbert and Thordarson, 2016) and by our 3:1 binding model, which we introduced in previous publication (Ustrnul et al., 2019). In Bindfit we used 2:1 NMR binding model with "full flavor", which do not assume any specific relation: (i) between chemical shifts of HG and HG_2; (ii) between stepwise association constants (K_1, K_2). The 3:1 binding model was adapted to studied system of HB donors and macrocycle using following constrains: (i) $K_{1obs} > K_{2obs} > K_{3obs}$ and (ii) and δ_{HG1}, δ_{HG2}, $\delta_{HG3} \geq \delta_{EXPmax}$ -5 ($\delta_{EXPmax} - \delta_{EXPmin}$); to prevent the chemical shift δ of complexed guest (HG_x) from diverging extremely from experimentally observed values.

The titration data were fitted using **NumPy** (1.10.2) and **SciPy** (0.18.1) libraries of Python 3. The script allows for simultaneous fitting of several datasets, which significantly improves stability of the fit results. This is essential in the case of 1:3 and 1:4 binding calculations. The **leastsq** function (implementation of the Levenberg-Marquadt algorithm) was used to determine the parameter set including the association constants K_1, K_2, and K_3 that minimizes $\chi^2 = \sum[f(x_i) - y_i]^2$, where $f(x_i)$ is the theoretical value of the traced quantity (chemical shift) for a given pair of the host and guest concentration $x_i = (c_{host0,i}, c_{guest0,i})$; y_i is the corresponding experimental value. The concentrations of the free host and guest molecules as well as their complexes are calculated

numerically (function **scipy.fsolve**), using the definition of the binding constants and the mass balance equations.

2 Structures of hosts and guest used in the study

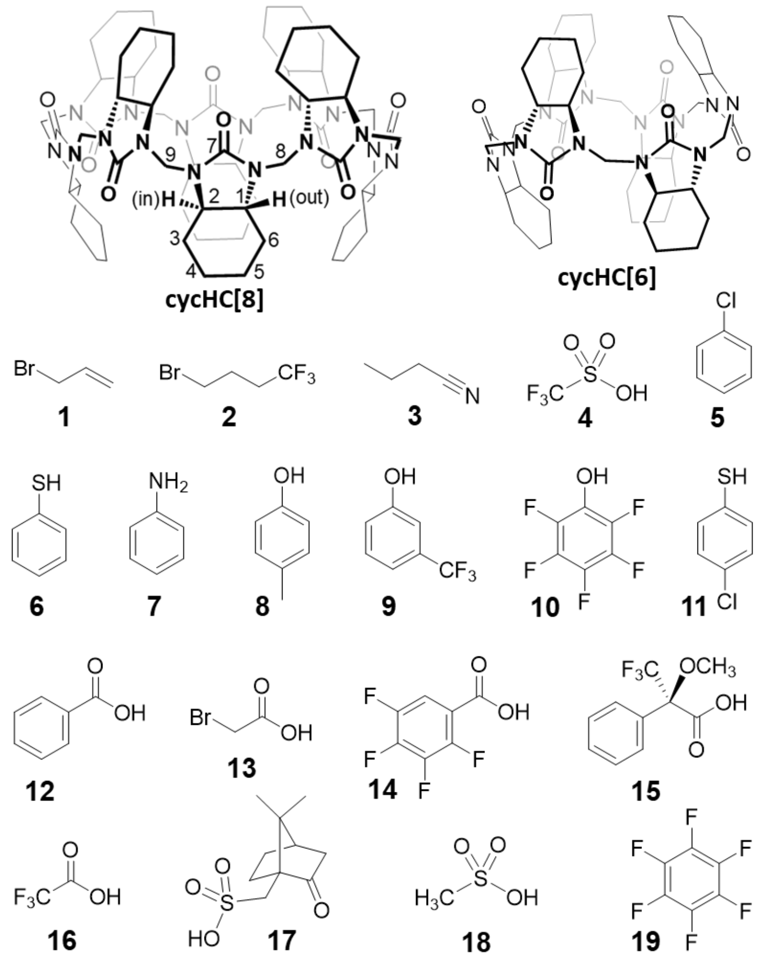

Scheme 1. Structures of macrocyclic hosts and small guest molecules used in the study.

3 Anion binding with cycHC[6]

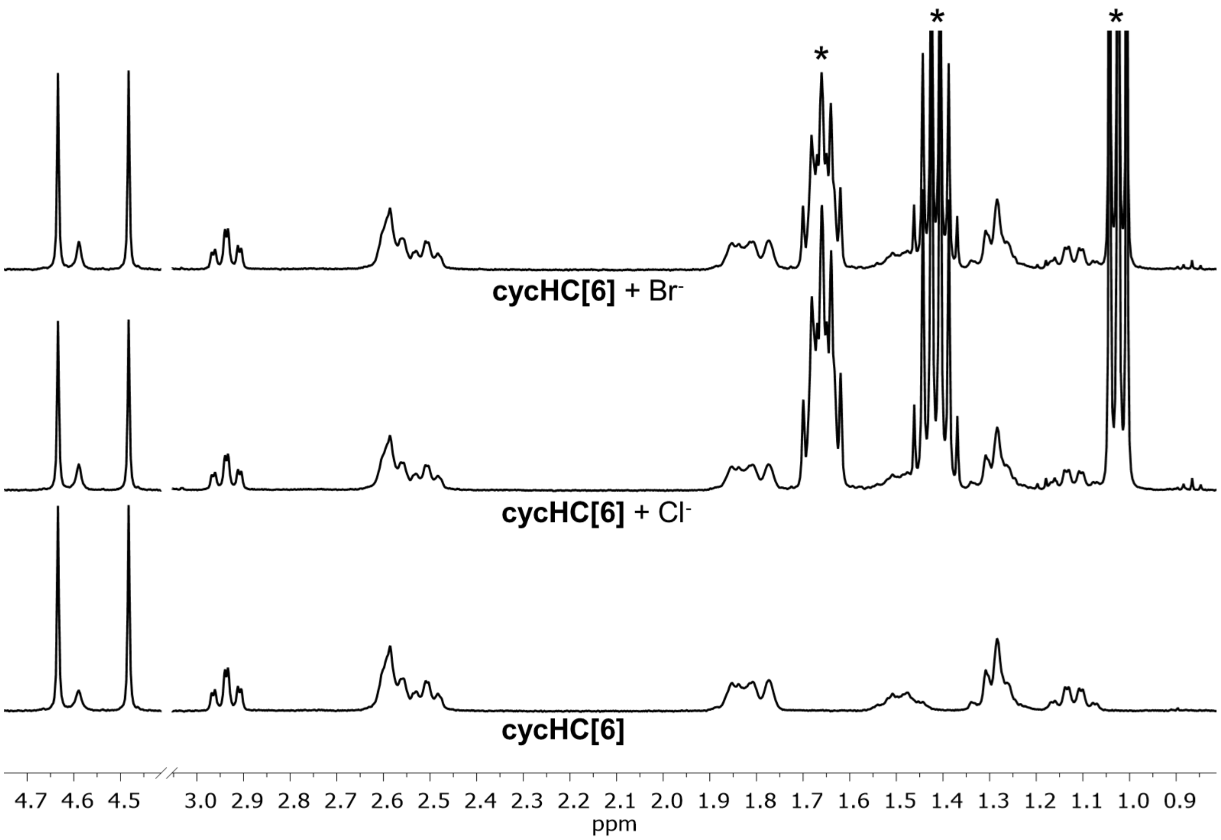

Figure S1. ^{1}H NMR spectra of free cycHC[6] in MeOD-d_4 (0.8 mM) and in the presence of chloride (8 equiv) and bromide (5 equiv) anions from tetrabutylammonium salts (cation signals assigned with asterisk). Measured 18 h after the salt addition.

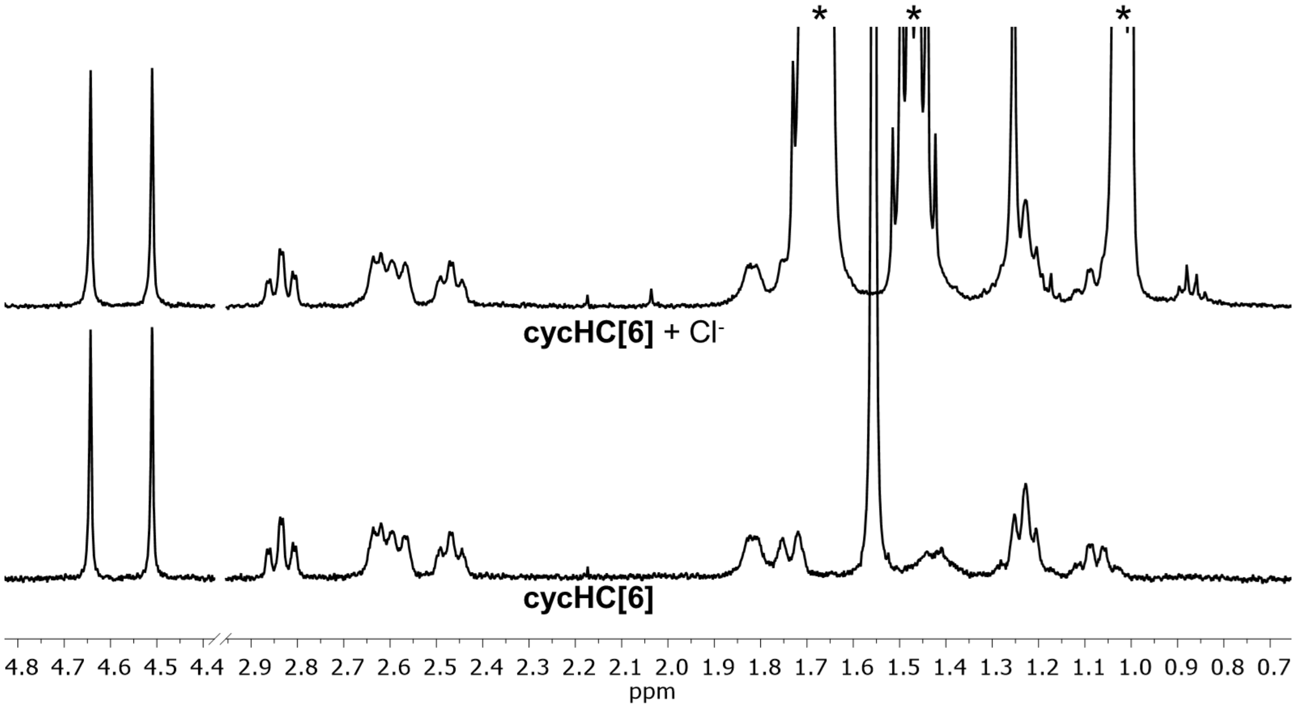

Figure S2. ^{1}H NMR spectra of free cycHC[6] in $CDCl_3$ (1.2 mM) and in the presence of tetrabutylammonium chloride (12 equiv) (cation signals assigned with asterisk).

4 Screening of potential guests (1-9) in chloroform

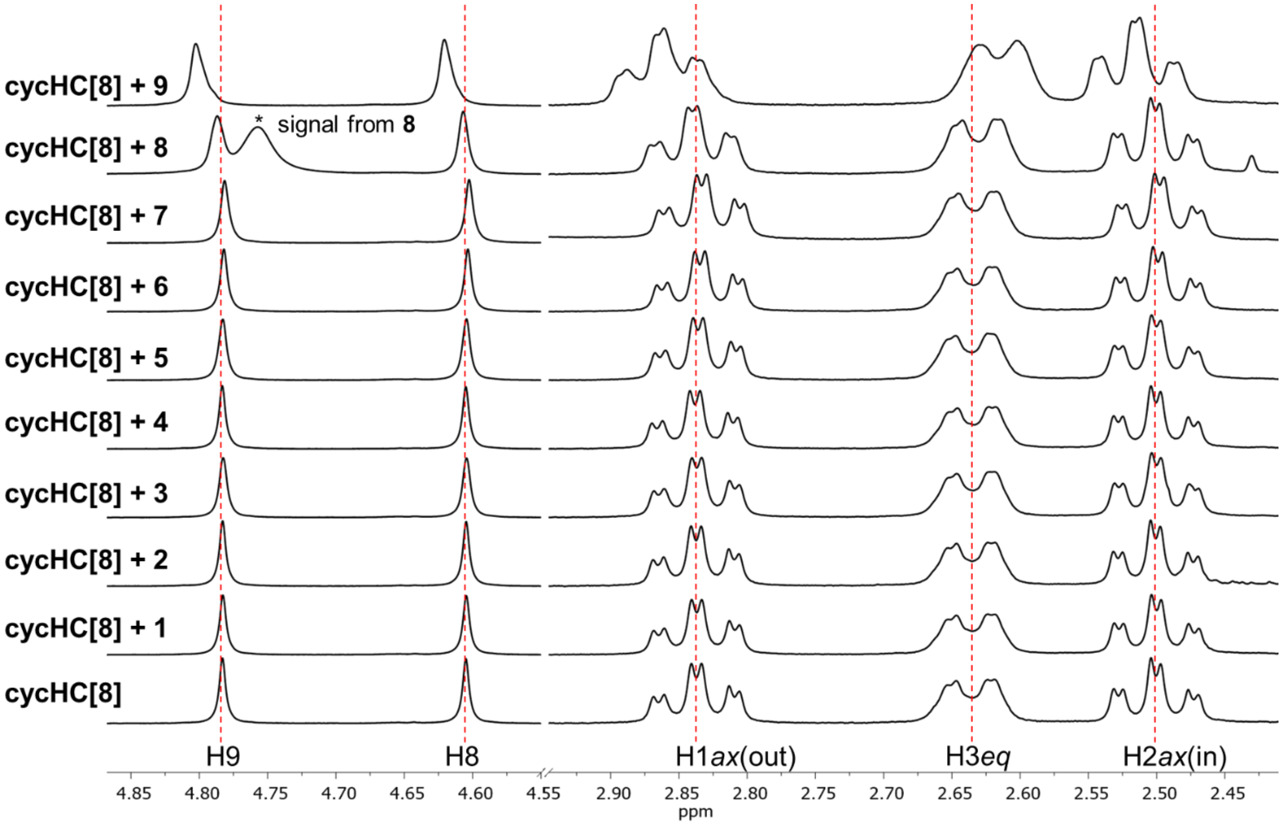

Figure S3. ^{1}H NMR spectra of free **cycHC[8]** (2.5 mM) in $CDCl_3$ and in the presence of gueststst **1-9** (40 equiv). Rest of the macrocycle's signals did not shifted or was covered with signals of guest. Additional signal in spectra with guest **8** corresponds to –OH group proton.

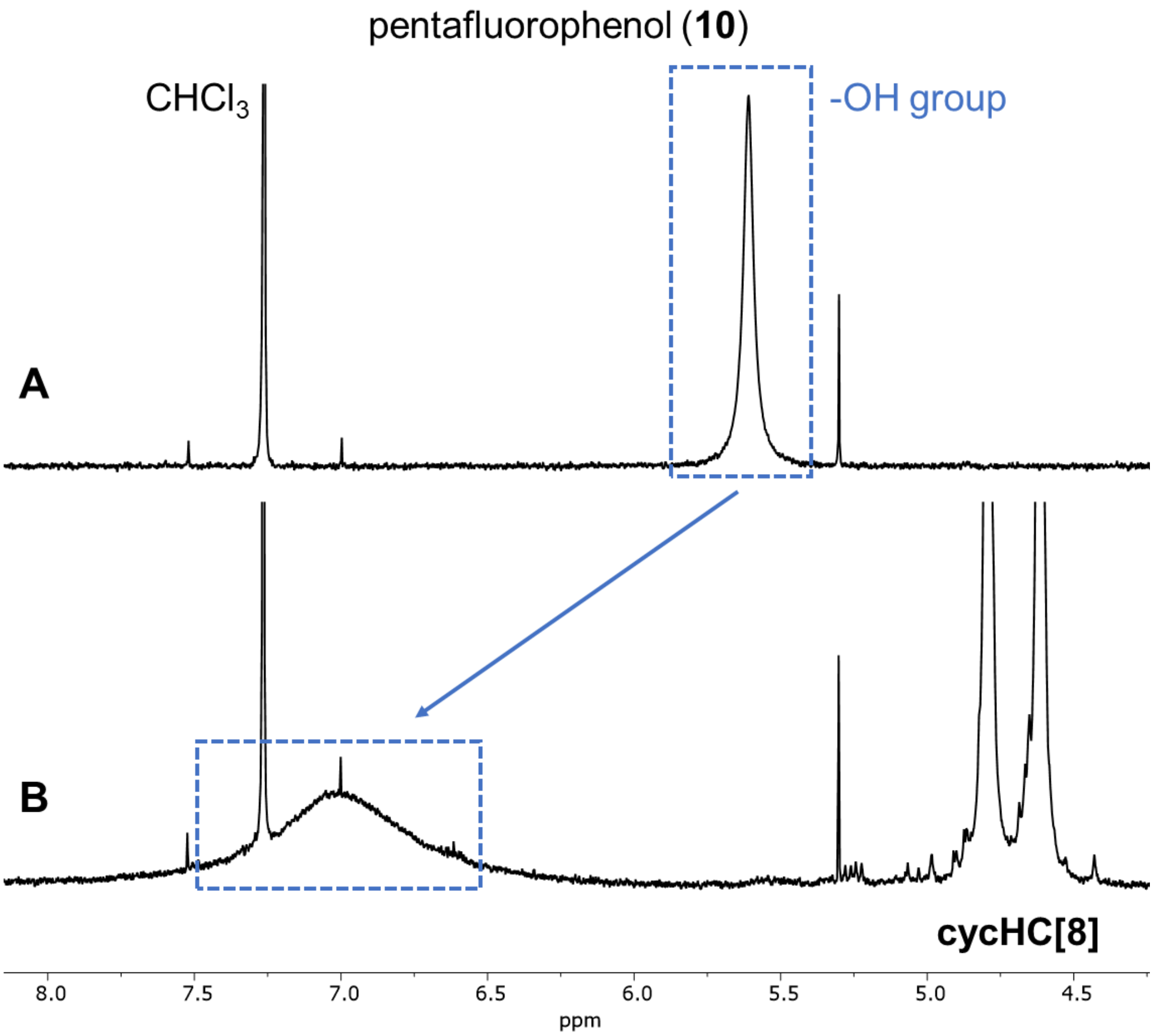

Figure S4. **(A)** ^{1}H NMR spectra of free pentafluorophenol (**10**) in $CDCl_3$ (60 mM) and **(B)** in the presence of 0.5 eq of **cycHC[8]** (30 mM). Downfield shift of acidic proton correspond with formation of hydrogen bond to macrocycle.

5 NMR titrations, Job plots and the data evaluation

5.1 Cooperativity evaluation criterions for cycHC[*n*]

Let's define a stepwise association constant K_i for an interaction between a substrate (host) M having m identical, independent binding sites and a ligand (guest) L having 1 binding site (Ercolani, 2003).

$$\mathrm{M} + \mathrm{L} \xrightleftharpoons{K_1} \mathrm{ML}$$

$$\mathrm{ML} + \mathrm{L} \xrightleftharpoons{K_2} \mathrm{ML_2}$$

$$\vdots$$

$$\mathrm{ML_{i-1}} + \mathrm{L} \xrightleftharpoons{K_i} \mathrm{ML_i}$$

$$\vdots$$

$$\mathrm{ML_{m-1}} + \mathrm{L} \xrightleftharpoons{K_m} \mathrm{ML_m}$$

Evaluation of stepwise association constants has to take in count the number of equal binding sites on same molecule of host (Ercolani, 2003; Thordarson, 2011). It can be derived what should be the ratio of stepwise association constants in systems with no cooperativity and this ratio can be used as a criterion to asses cooperativity. For a two-step binding it is generally know that non-cooperative system exhibits ratio of stepwise constants according equation $K_1 = 4\,K_2$.

Here is a general equation for ratio between any two consecutive K_i in system $\mathrm{ML_m}$:

$$\frac{\boldsymbol{K_{i+1}}}{\boldsymbol{K_i}} = \frac{\boldsymbol{i(m-i)}}{\boldsymbol{(i+1)(m-i+1)}}$$

If the ratio K_{i+1}/K_i is satisfied with determined association constants then the system is noncooperative or statistical. If the ratio value is higher than it would be for statistical system then we speak about positive cooperativity. If the ratio value is lower than it would be for statistical system then we speak about negative cooperativity.

The K_{i+1}/K_i ratios for noncooperative binding of guest to **cycHC[6]** (m = 6) based on equation above:
$K_2/K_1 = 5/12$
$K_3/K_2 = 8/15$
$K_4/K_3 = 9/16$
$K_5/K_4 = 8/15$
$K_6/K_5 = 5/12$

The K_{i+1}/K_i ratios for noncooperative binding of guest to **cycHC[8]** (m = 8) based on equation above:
$K_2/K_1 = 7/16$
$K_3/K_2 = 12/21$
$K_4/K_3 = 15/24$
$K_5/K_4 = 16/25$
$K_6/K_5 = 15/24$
$K_7/K_6 = 12/21$
$K_8/K_7 = 7/16$

5.2 Normalization of titration curves

For the normalization of titration curves was used following formula:

$$N_x = 1 - \frac{\delta_x - \delta_y}{\delta_0 - \delta_y}$$

Where
$\mathbf{N_x}$ is normalized value of chemical shift at **x** equivalents of cycHC[n]
$\delta_{x\ equiv}$ is chemical shift of guest at **x** equivalents of cycHC[n]
$\delta_{0\ equiv}$ is chemical shift of guest at **0** equivalents of cycHC[n]
$\delta_{y\ equiv}$ is chemical shift of guest at **X** equivalents of cycHC[n], which is point for which we normalize a titration curve

5.3 NMR titration data for association constants evaluation

Following NMR data for trifluoroacetic acid **16** (Table S13-S17) and methanesulfonic acid **18** (Table S19-S22) were used for qualitative comparison between binding of trifluoroacetic acid **16** with **cycHC[6]** and **cycHC[8]** and for qualitative comparison between binding of **cycHC[6]** with guest **16** and **18**. All of these data for **16** and **18** were also used for evaluation of first three apparent stepwise association constants with our 3:1 binding model (Ustrnul et al., 2019). Following constrains were used to improve a chance for a binding model to converge: (i) $K_{1obs} > K_{2obs} > K_{3obs}$ and (ii) and δ_{HG1}, δ_{HG2}, $\delta_{HG3} \geq \delta_{EXPmax} - 5(\delta_{EXPmax} - \delta_{EXPmin})$; to prevent the chemical shift δ of complexed guest (HG_x) from diverging extremely from experimentally observed values.

Note:
Even if we would be adding a guest to the macrocycle, we could not easily distinguish between complexes of different stoichiometry, which would eventually lead to large errors in evaluating stepwise K_a. Moreover, the number of variables in the evaluation process, which has to be fitted simultaneously, grows exponentially with the increasing stoichiometry, hence obtaining reliable values of all six or eight stepwise association constants for **cycHC[6]** or **cycHC[8]** is very difficult. Determination of all K_a would require excessive amount of experimental data from wide range of concentrations, which can be often impossible to do, for example due to a limited solubility of host or guest.

5.4 NMR titration data

Table S1. ^{1}H NMR titration data for 2 mM thiophenol (guest **6**) with **cycHC[6]**, in $CDCl_3$ and corresponding spectra below.

Nr.	Guest 6 concentration, M	cycHC[6] concentration, M	molar ratio, equiv	H1, ppm	H1 normalized at 10 equiv
1	1.98E-03	0.00E+00	0.00	7.2321	0.0000
2	1.98E-03	9.90E-04	0.50	7.2317	0.0506
3	1.98E-03	2.03E-03	1.02	7.2318	0.0380
4	1.98E-03	4.02E-03	2.03	7.2305	0.2025
5	1.98E-03	5.98E-03	3.01	7.2298	0.2911
6	1.98E-03	7.96E-03	4.01	7.2292	0.3671
7	1.98E-03	9.95E-03	5.01	7.2283	0.4810
8	1.98E-03	1.19E-02	6.02	7.2273	0.6076
9	1.98E-03	1.39E-02	7.01	7.2267	0.6835
10	1.98E-03	1.59E-02	8.01	7.2258	0.7975
11	1.98E-03	1.79E-02	9.01	7.2251	0.8861
12	1.98E-03	1.98E-02	10.01	7.2242	1.0000
13	1.98E-03	2.18E-02	11.00	7.2236	1.0759

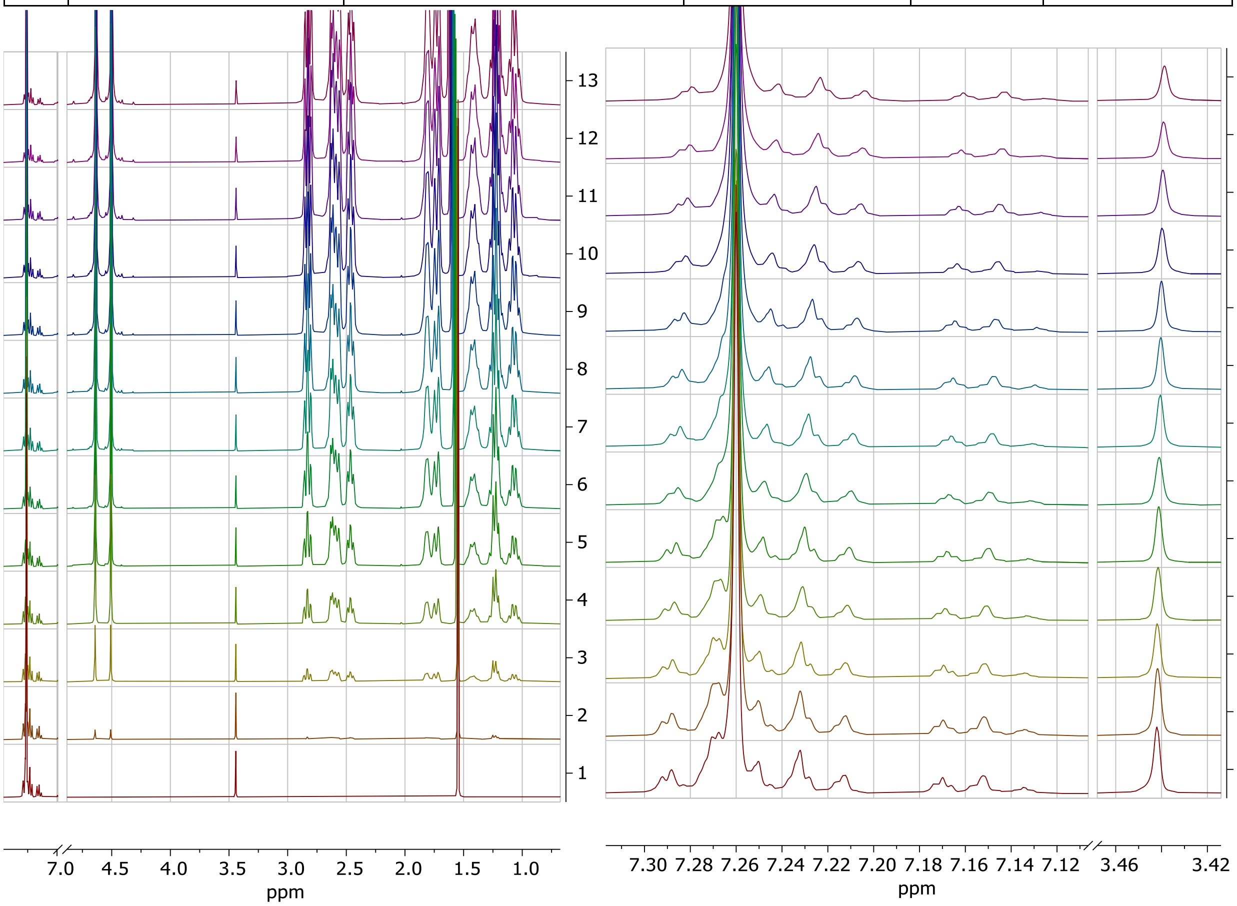

Table S2. ^{19}F NMR titration data for 2 mM 3-(trifluoromethyl)phenol (guest **9**) with **cycHC[6]**, in $CDCl_3$ in presence of hexafluorobenzene (**19**) and corresponding spectra below.

Nr.	Guest 9 concentration, M	cycHC[6] concentration, M	molar ratio, equiv	F1, ppm	F1 normalized at 10 equiv
1	1.97E-03	0.00E+00	0.00	-62.8125	0.0000
2	1.97E-03	9.81E-04	0.50	-62.8044	0.0904
3	1.97E-03	1.94E-03	0.98	-62.7962	0.1819
4	1.97E-03	3.90E-03	1.98	-62.7827	0.3326
5	1.97E-03	5.88E-03	2.98	-62.7718	0.4542
6	1.97E-03	7.86E-03	3.99	-62.7609	0.5759
7	1.97E-03	9.84E-03	4.99	-62.7528	0.6663
8	1.97E-03	1.18E-02	5.99	-62.7447	0.7567
9	1.97E-03	1.38E-02	6.99	-62.7392	0.8181
10	1.97E-03	1.57E-02	7.99	-62.7338	0.8783
11	1.97E-03	1.77E-02	8.99	-62.7284	0.9386
12	1.97E-03	1.97E-02	9.99	-62.7229	1.0000
13	1.97E-03	2.16E-02	10.98	-62.7175	1.0603
14	1.97E-03	2.38E-02	12.05	-62.7148	1.0904

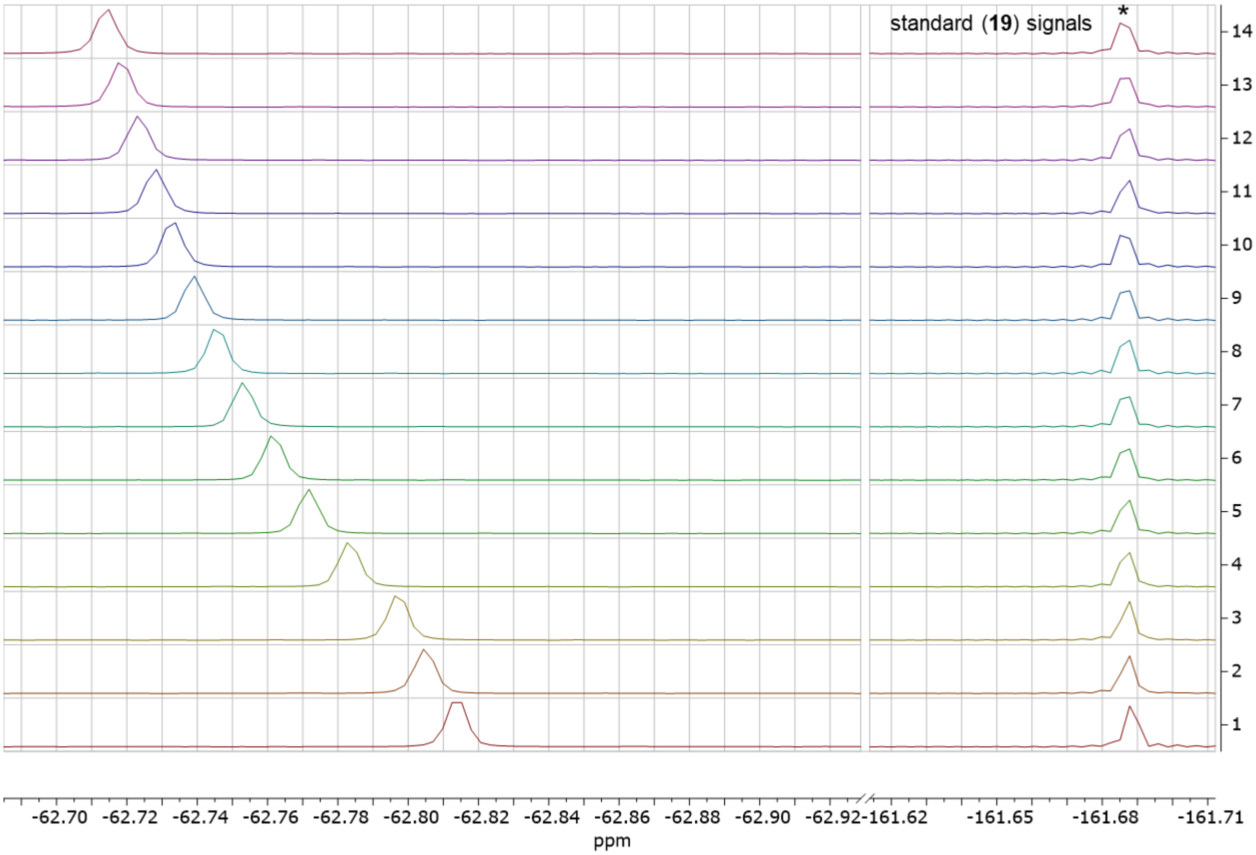

Table S3. ^{19}F NMR titration data for 2 mM pentafluorophenol (guest **10**) with **cycHC[6]**, in $CDCl_3$ and corresponding spectra below.

Nr.	Guest 10 concentration, M	cycHC[6] concentration, M	molar ratio, equiv	F1, ppm	F1 normalized at 10 equiv	F2, ppm	F3, ppm
1	2.04E-03	0.00E+00	0.00	-163.402	0.0000	-163.455	-168.031
2	2.04E-03	1.03E-03	0.50	-163.357	0.1125	-163.543	-168.182
3	2.04E-03	2.04E-03	1.00	-163.319	0.2062	-163.622	-168.313
4	2.04E-03	3.07E-03	1.51	-163.283	0.2949	-163.694	-168.433
5	2.04E-03	4.09E-03	2.01	-163.252	0.3730	-163.757	-168.539
6	2.04E-03	6.13E-03	3.01	-163.197	0.5083	-163.865	-168.719
7	2.04E-03	8.17E-03	4.01	-163.152	0.6201	-163.954	-168.868
8	2.04E-03	1.22E-02	6.01	-163.084	0.7881	-164.086	-169.090
9	2.04E-03	1.63E-02	8.01	-163.035	0.9103	-164.184	-169.252
10	2.04E-03	2.04E-02	10.02	-162.999	1.0000	-164.255	-169.371
11	2.04E-03	2.45E-02	12.02	-162.968	1.0761	-164.315	-169.471
12	2.04E-03	2.86E-02	14.02	-162.944	1.1368	-164.363	-169.552
13	2.04E-03	3.26E-02	16.02	-162.924	1.1854	-164.398	-169.610
14	2.04E-03	3.67E-02	18.02	-162.907	1.2283	-164.435	-169.673
15	2.04E-03	3.93E-02	19.29	-162.897	1.2525	-164.453	-169.703

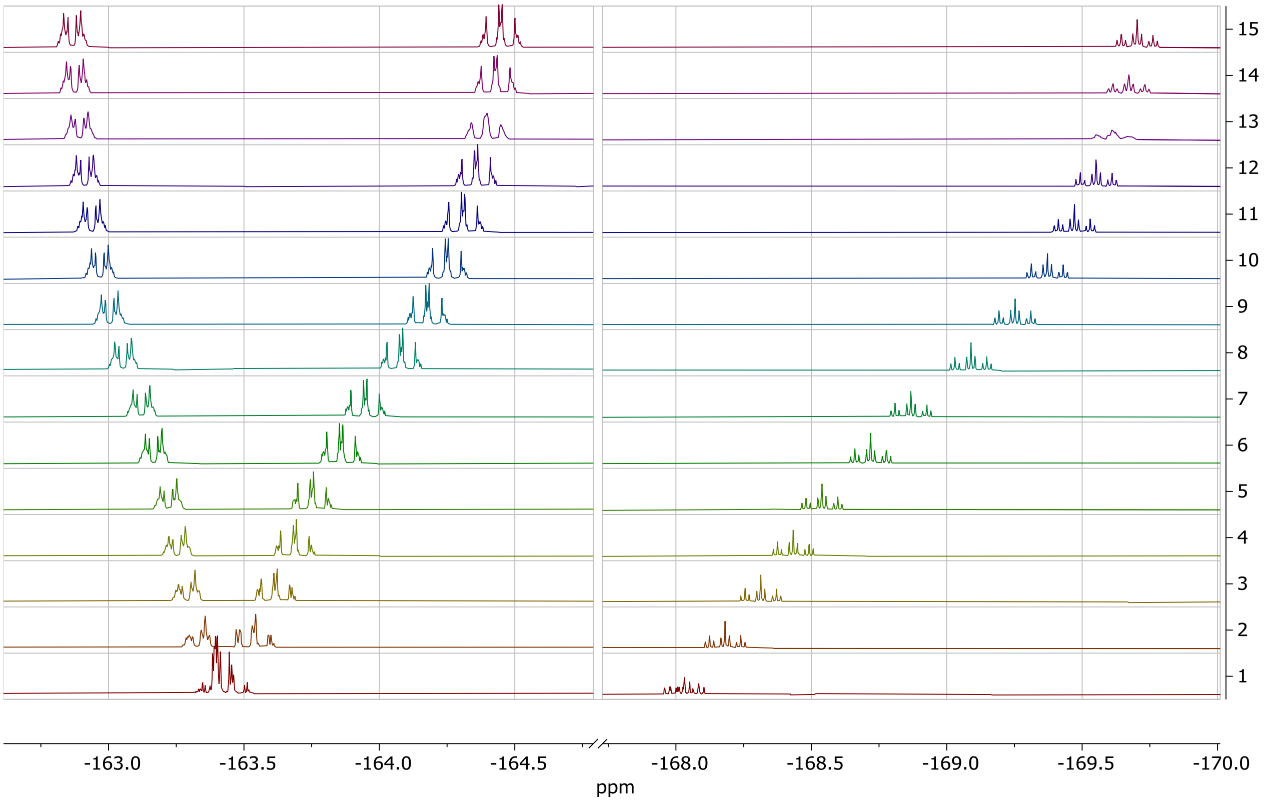

Table S4. ^{1}H NMR titration data for 2 mM 4-chlorothiophenol (guest **11**) with **cycHC[6]**, in $CDCl_3$ and corresponding spectra below.

Nr.	Guest 11 concentration, M	cycHC[6] concentration, M	molar ratio, equiv	H1, ppm	H1 normalized at 10 equiv
1	1.97E-03	0.00E+00	0.00	7.2067	0.0000
2	1.97E-03	9.94E-04	0.49	7.2061	0.0759
3	1.97E-03	1.96E-03	0.99	7.2061	0.0759
4	1.97E-03	3.91E-03	1.98	7.2051	0.2025
5	1.97E-03	5.92E-03	2.99	7.2044	0.2911
6	1.97E-03	7.89E-03	3.98	7.2036	0.3924
7	1.97E-03	9.86E-03	4.98	7.2029	0.4810
8	1.97E-03	1.18E-02	5.98	7.2019	0.6076
9	1.97E-03	1.38E-02	6.98	7.2013	0.6835
10	1.97E-03	1.58E-02	7.99	7.2004	0.7975
11	1.97E-03	1.78E-02	8.99	7.1997	0.8861
12	1.97E-03	1.97E-02	10.00	7.1988	1.0000
13	1.97E-03	2.17E-02	11.01	7.1982	1.0759
14	1.97E-03	2.39E-02	11.56	7.1975	1.1646

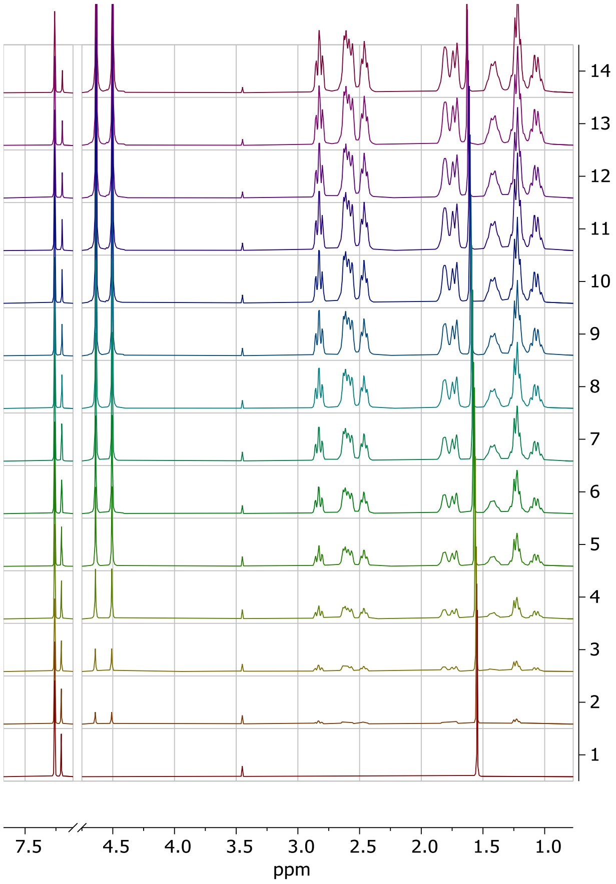

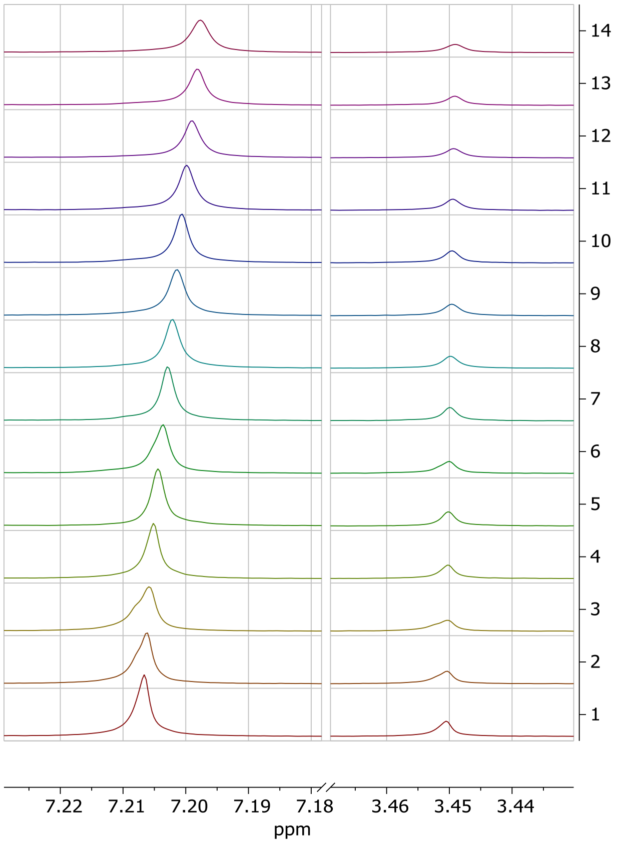

Table S5. ^{1}H NMR titration data for 2 mM benzoic acid (guest **12**) with **cycHC[6]**, in $CDCl_3$ and corresponding spectra below.

Nr.	Guest 12 concentration, M	cycHC[6] concentration, M	molar ratio, equiv	H1, ppm	H1 normalized at 10 equiv	H2, ppm	H3, ppm
1	1.97E-03	0.00E+00	0.00	8.0947	0.0000	7.6167	7.4799
2	1.97E-03	9.94E-04	0.50	8.0928	0.0640	7.6148	7.4784
3	1.97E-03	1.96E-03	1.00	8.0909	0.1279	7.6129	7.4771
4	1.97E-03	3.91E-03	1.98	8.0873	0.2492	7.6090	7.4738
5	1.97E-03	5.92E-03	3.00	8.0837	0.3704	7.6051	7.4708
6	1.97E-03	7.89E-03	4.00	8.0807	0.4714	7.6017	7.4684
7	1.97E-03	9.86E-03	5.00	8.0779	0.5657	7.5983	7.4662
8	1.97E-03	1.18E-02	6.00	8.0778	0.5690	7.5982	7.4661
9	1.97E-03	1.38E-02	7.00	8.0724	0.7508	7.5922	7.4614
10	1.97E-03	1.58E-02	8.00	8.0697	0.8418	7.5895	7.4590
11	1.97E-03	1.78E-02	9.01	8.0675	0.9158	7.5867	7.4571
12	1.97E-03	1.97E-02	10.00	8.0650	1.0000	7.5839	7.4549
13	1.97E-03	2.17E-02	11.00	8.0629	1.0707	7.5818	7.4532
14	1.97E-03	2.39E-02	12.12	8.0603	1.1582	7.5788	7.4509

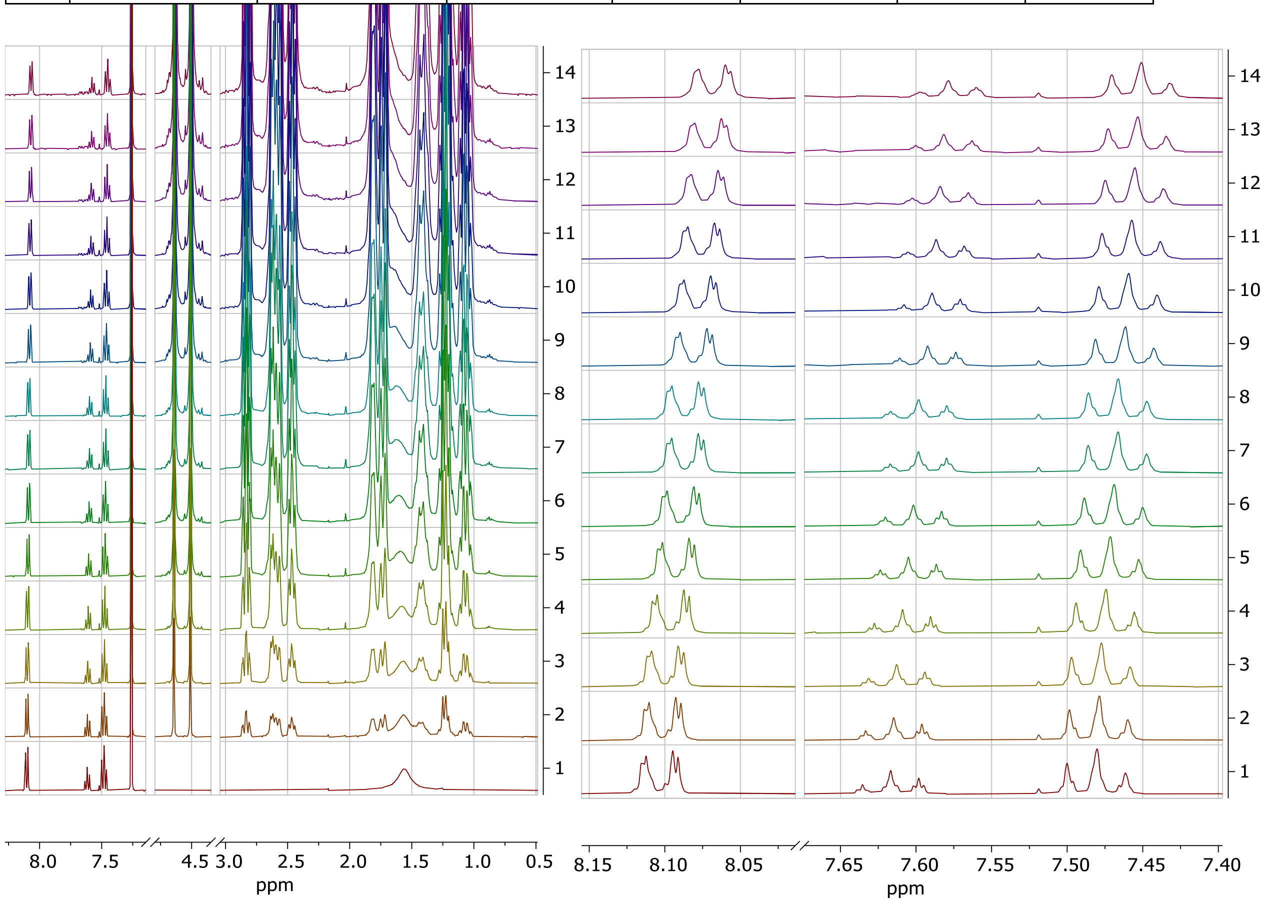

Table S6. ^{1}H NMR titration data for 2 mM bromoacetic acid (guest **13**) with **cycHC[6]**, in $CDCl_3$ and corresponding spectra below.

Nr.	Guest 13 concentration, M	cycHC[6] concentration, M	molar ratio, equiv	H1, ppm	H1 normalized at 10 equiv
1	1.99E-03	0.00E+00	0.00	3.8999	0.0000
2	1.99E-03	9.98E-04	0.50	3.8948	0.1063
3	1.99E-03	1.99E-03	1.00	3.8903	0.2000
4	1.99E-03	3.00E-03	1.51	3.8862	0.2854
5	1.99E-03	3.99E-03	2.01	3.8826	0.3604
6	1.99E-03	5.98E-03	3.01	3.8763	0.4917
7	1.99E-03	7.96E-03	4.00	3.8711	0.6000
8	1.99E-03	1.20E-02	6.00	3.8629	0.7708
9	1.99E-03	1.59E-02	8.00	3.8567	0.9000
10	1.99E-03	1.99E-02	10.00	3.8519	1.0000
11	1.99E-03	2.39E-02	12.00	3.8479	1.0833
12	1.99E-03	2.79E-02	14.01	3.8443	1.1583
13	1.99E-03	3.19E-02	16.01	3.8413	1.2208
14	1.99E-03	3.59E-02	18.01	3.8386	1.2771
15	1.99E-03	4.56E-02	22.90	3.8329	1.3958

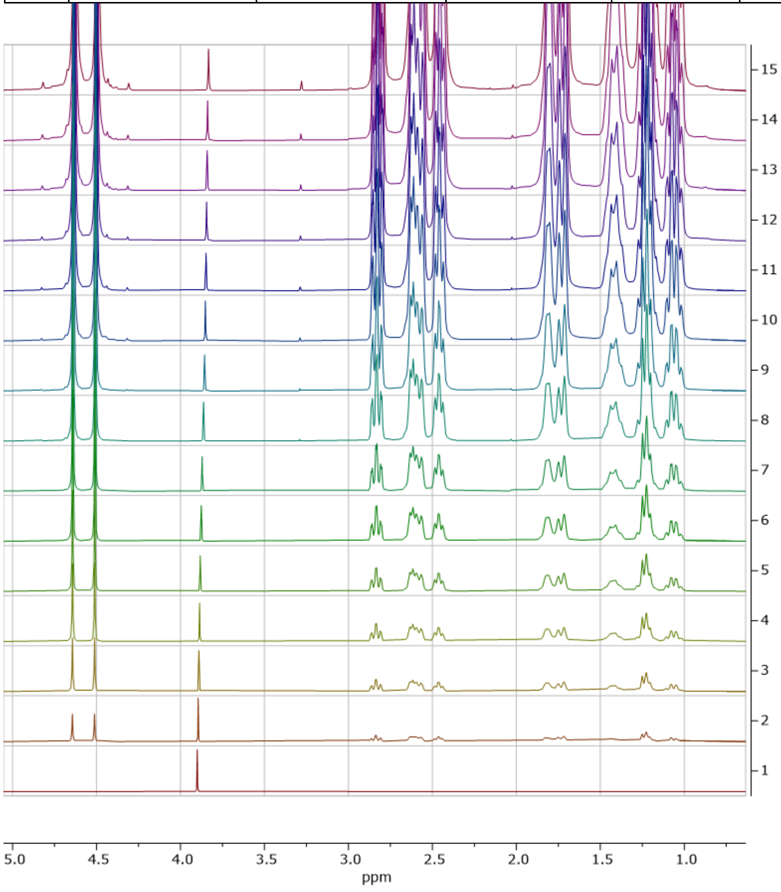

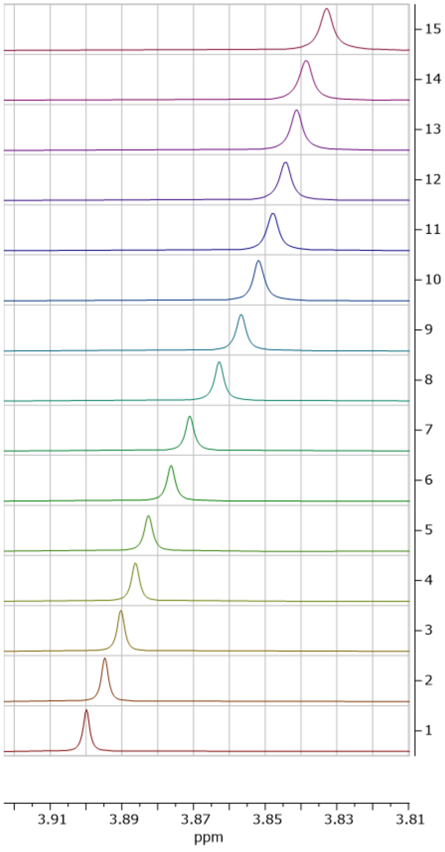

Table S7. ^{19}F NMR titration data for 2 mM 2,3,4,5-tetrafluorobenzoic acid (guest **14**) with **cycHC[6]**, in $CDCl_3$ and corresponding spectra below.

Nr.	Guest 14 concentration, M	cycHC[6] concentration, M	molar ratio, equiv	F1, ppm	F1 normalized at 10 equiv	F2, ppm	F3, ppm	F4, ppm
1	2.03E-03	0.00E+00	0.00	-132.812	0.0000	-137.262	-145.321	-152.754
2	2.03E-03	1.02E-03	0.50	-132.933	0.1046	-137.343	-145.524	-152.835
3	2.03E-03	2.03E-03	1.00	-133.048	0.2044	-137.405	-145.687	-152.894
4	2.03E-03	3.05E-03	1.50	-133.153	0.2955	-137.463	-145.836	-152.947
5	2.03E-03	4.06E-03	2.00	-133.248	0.3778	-137.515	-145.971	-152.997
6	2.03E-03	6.09E-03	3.00	-133.410	0.5184	-137.604	-146.200	-153.080
7	2.03E-03	8.13E-03	4.00	-133.541	0.6321	-137.676	-146.385	-153.147
8	2.03E-03	1.22E-02	6.01	-133.735	0.8008	-137.783	-146.657	-153.246
9	2.03E-03	1.63E-02	8.01	-133.869	0.9176	-137.857	-146.845	-153.316
10	2.03E-03	2.03E-02	10.00	-133.964	1.0000	-137.910	-146.977	-153.365
11	2.03E-03	2.44E-02	12.00	-134.038	1.0641	-137.951	-147.080	-153.403
12	2.03E-03	2.84E-02	14.00	-134.095	1.1134	-137.984	-147.159	-153.433
13	2.03E-03	3.25E-02	15.99	-134.139	1.1513	-138.009	-147.221	-153.457
14	2.03E-03	3.66E-02	17.99	-134.175	1.1829	-138.030	-147.272	-153.476
15	2.03E-03	3.91E-02	19.25	-134.195	1.2003	-138.042	-147.300	-153.487

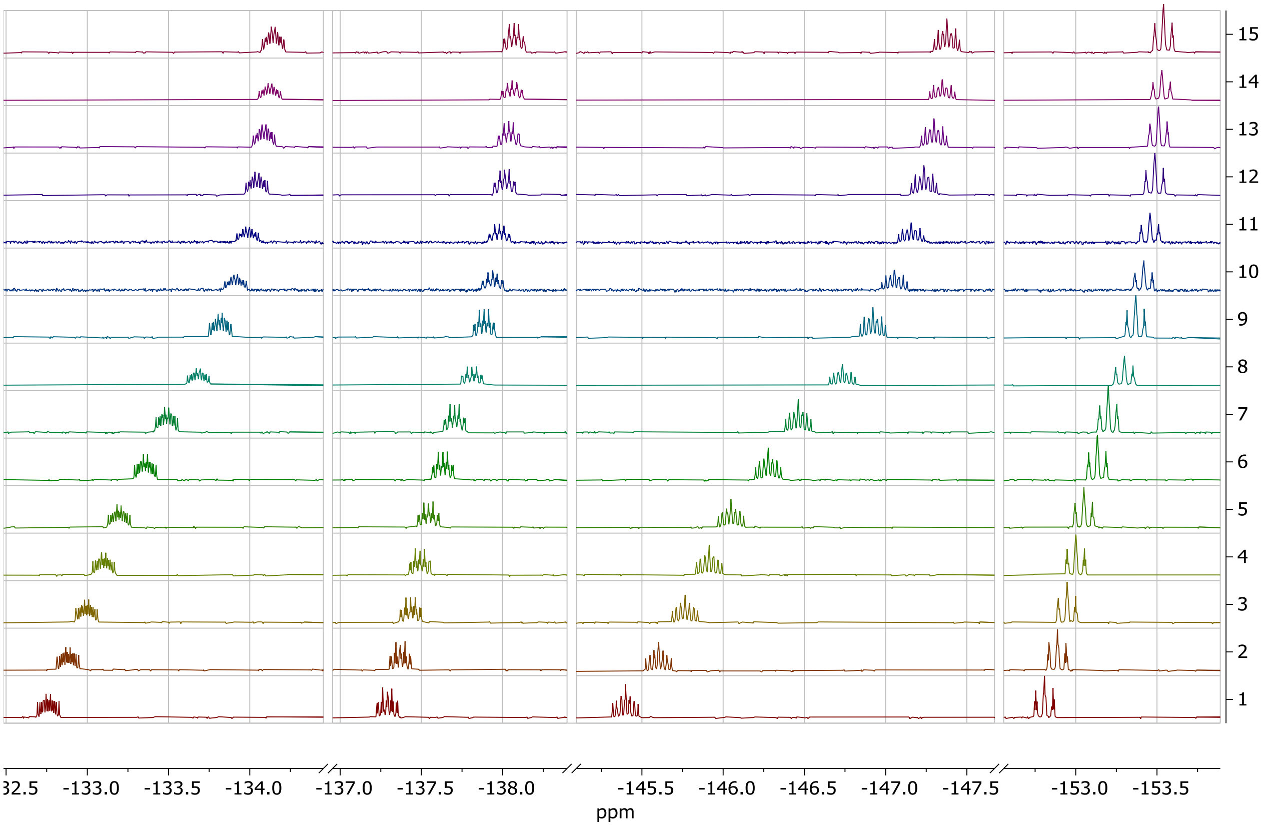

Table S8. ^{19}F NMR titration data for 2 mM *R*-Mosher's acid (guest *R*-**15**) with **cycHC[6]**, in $CDCl_3$ and corresponding spectra below.

Nr.	Guest R-15 concentration, M	cycHC[6] concentration, M	molar ratio, equiv	F1, ppm	F1 normalized at 10 equiv	F1 normalized at 11 equiv
1	2.03E-03	0.00E+00	0.00	-70.650	0.0000	0.0000
2	2.03E-03	1.02E-03	0.50	-70.737	0.1412	0.1369
3	2.03E-03	2.02E-03	1.00	-70.810	0.2589	0.2511
4	2.03E-03	4.07E-03	2.01	-70.923	0.4411	0.4277
5	2.03E-03	6.08E-03	3.00	-71.009	0.5785	0.5609
6	2.03E-03	8.11E-03	4.01	-71.073	0.6824	0.6616
7	2.03E-03	1.02E-02	5.01	-71.124	0.7648	0.7414
8	2.03E-03	1.22E-02	6.01	-71.165	0.8313	0.8060
9	2.03E-03	1.42E-02	7.01	-71.198	0.8844	0.8574
10	2.03E-03	1.62E-02	8.00	-71.226	0.9294	0.9010
11	2.03E-03	1.82E-02	9.01	-71.251	0.9687	0.9392
12	2.03E-03	2.03E-02	10.01	-71.270	1.0000	0.9695
13	2.03E-03	2.23E-02	11.01	-71.289	1.0314	1.0000

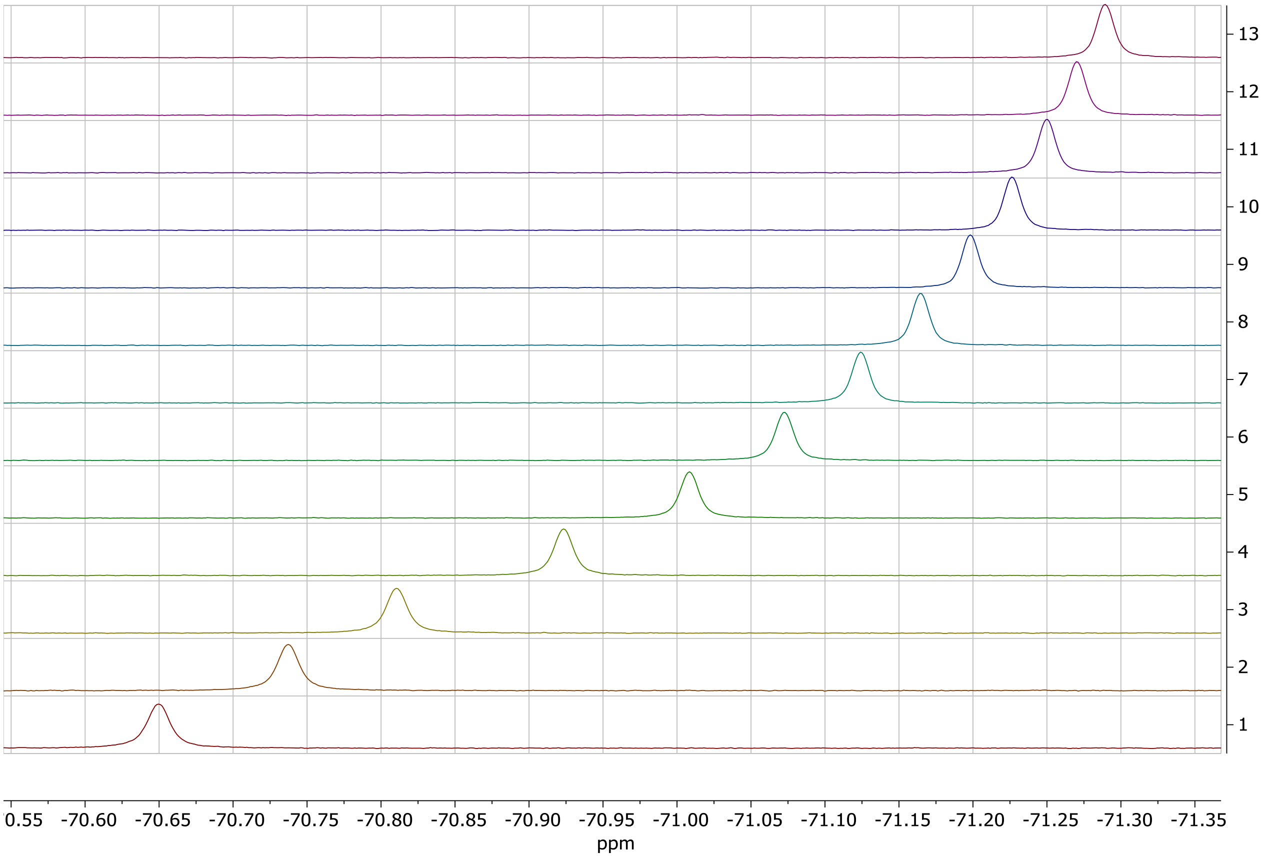

Table S9. Second independent ^{19}F NMR titration data for 2 mM *R*-Mosher's acid (guest *R*-**15**) with **cycHC[6]**, in $CDCl_3$.

Nr.	Guest R-15 concentration, M	cycHC[6] concentration, M	molar ratio, equiv	F1, ppm	F1 normalized at 11 equiv
1	2.03E-03	0.00E+00	0.00	-70.666	0.0000
2	2.03E-03	1.01E-04	0.05	-70.676	0.0156
3	2.03E-03	2.01E-04	0.10	-70.684	0.0291
4	2.03E-03	3.02E-04	0.15	-70.692	0.0428
5	2.03E-03	4.02E-04	0.20	-70.700	0.0561
6	2.03E-03	1.02E-03	0.50	-70.748	0.1351
7	2.03E-03	2.03E-03	1.00	-70.815	0.2449
8	2.03E-03	4.05E-03	2.00	-70.921	0.4196
9	2.03E-03	6.07E-03	2.99	-71.001	0.5501
10	2.03E-03	1.01E-02	4.99	-71.112	0.7335
11	2.03E-03	1.42E-02	6.99	-71.185	0.8531
12	2.03E-03	1.82E-02	8.99	-71.236	0.9380
13	2.03E-03	2.23E-02	10.99	-71.274	1.0000
14	2.03E-03	2.64E-02	12.99	-71.304	1.0487
15	2.03E-03	3.07E-02	15.12	-71.328	1.0887

Table S10. ^{19}F NMR titration data for 2 mM *S*-Mosher's acid (guest *S*-**15**) with **cycHC[6]**, in $CDCl_3$.

Nr.	Guest S-15 concentration, M	cycHC[6] concentration, M	molar ratio, equiv	F1, ppm	F1 normalized at 11 equiv
1	2.01E-03	0.00E+00	0.00	-70.655	0.0000
2	2.01E-03	1.06E-04	0.05	-70.665	0.0167
3	2.01E-03	2.12E-04	0.11	-70.674	0.0313
4	2.01E-03	3.18E-04	0.16	-70.683	0.0459
5	2.01E-03	4.24E-04	0.21	-70.691	0.0590
6	2.01E-03	1.02E-03	0.51	-70.737	0.1344
7	2.01E-03	2.03E-03	1.01	-70.805	0.2461
8	2.01E-03	4.05E-03	2.01	-70.912	0.4220
9	2.01E-03	6.05E-03	3.01	-70.992	0.5526
10	2.01E-03	1.01E-02	5.01	-71.103	0.7350
11	2.01E-03	1.41E-02	7.01	-71.177	0.8555
12	2.01E-03	1.81E-02	9.00	-71.228	0.9385
13	2.01E-03	2.22E-02	11.00	-71.265	1.0000
14	2.01E-03	2.62E-02	13.00	-71.294	1.0475
15	2.01E-03	3.22E-02	15.99	-71.325	1.0990

Table S11. ^{1}H NMR titration data for 2 mM *R*-Mosher's acid (guest *R*-**15**) with **cycHC[6]**, in $CDCl_3$

Nr.	Guest R-15 concentration, M	cycHC[6] concentration, M	molar ratio, equiv	H1, ppm	H1 normalized at 4 equiv
1	1.88E-03	0.00E+00	0.00	7.456	0.0000
2	1.88E-03	9.24E-05	0.05	7.455	0.0072
3	1.88E-03	1.85E-04	0.10	7.455	0.0179
4	1.88E-03	2.76E-04	0.15	7.455	0.0323
5	1.88E-03	3.74E-04	0.20	7.454	0.0466
6	1.88E-03	5.61E-04	0.30	7.453	0.0860
7	1.88E-03	7.48E-04	0.40	7.452	0.1254
8	1.88E-03	9.39E-04	0.50	7.451	0.1649
9	1.88E-03	1.32E-03	0.70	7.449	0.2401
10	1.88E-03	1.88E-03	1.00	7.446	0.3369
11	1.88E-03	2.63E-03	1.40	7.443	0.4588
12	1.88E-03	3.75E-03	2.00	7.438	0.6165
13	1.88E-03	5.63E-03	3.00	7.432	0.8315
14	1.88E-03	7.50E-03	4.00	7.428	1.0000
15	1.88E-03	9.29E-03	4.95	7.424	1.1290

Table S12. ^{1}H NMR titration data for 2 mM *S*-Mosher's acid (guest *S*-**15**) with **cycHC[6]**, in $CDCl_3$ and corresponding spectra below.

Nr.	Guest S-15 concentration, M	cycHC[6] concentration, M	molar ratio, equiv	H1, ppm	H1 normalized at 4 equiv
1	2.18E-03	0.00E+00	0.00	7.455	0.0000
2	2.18E-03	1.05E-04	0.05	7.455	0.0132
3	2.18E-03	2.16E-04	0.10	7.454	0.0329
4	2.18E-03	3.27E-04	0.15	7.454	0.0526
5	2.18E-03	4.37E-04	0.20	7.453	0.0724
6	2.18E-03	6.56E-04	0.30	7.452	0.1118
7	2.18E-03	8.73E-04	0.40	7.451	0.1513
8	2.18E-03	1.09E-03	0.50	7.449	0.1908
9	2.18E-03	1.53E-03	0.70	7.447	0.2664
10	2.18E-03	2.19E-03	1.00	7.444	0.3684
11	2.18E-03	3.06E-03	1.40	7.440	0.4868
12	2.18E-03	4.37E-03	2.00	7.436	0.6382
13	2.18E-03	6.55E-03	3.00	7.429	0.8454
14	2.18E-03	8.74E-03	4.00	7.425	1.0000
15	2.18E-03	9.61E-03	4.40	7.423	1.0559

Table S13. ^{19}F NMR titration data for 2 mM trifluoroacetic acid (guest **16**) with **cycHC[6]**, in $CDCl_3$ and corresponding spectra below.

Nr.	Guest 16 concentration, M	cycHC[6] concentration, M	molar ratio, equiv	F1, ppm	F1 normalized at 10 equiv	F1 normalized at 9 equiv	F1 normalized at 5 equiv	F1 normalized at 1 equiv
1	2.00E-03	0.00E+00	0.00	-75.577	0.0000	0.0000	0.0000	0.0000
2	2.00E-03	4.87E-04	0.24	-75.641	0.2247	0.2267	0.2434	0.3810
3	2.00E-03	9.90E-04	0.49	-75.687	0.3873	0.3907	0.4196	0.6565
4	2.00E-03	1.49E-03	0.74	-75.720	0.5004	0.5048	0.5420	0.8482
5	2.00E-03	1.98E-03	0.99	-75.745	0.5899	0.5951	0.6390	1.0000
6	2.00E-03	2.56E-03	1.28	-75.766	0.6626	0.6684	0.7178	1.1232
7	2.00E-03	3.18E-03	1.59	-75.783	0.7223	0.7287	0.7824	1.2244
8	2.00E-03	3.99E-03	1.99	-75.797	0.7735	0.7804	0.8380	1.3113
9	2.00E-03	6.00E-03	2.99	-75.818	0.8462	0.8537	0.9167	1.4345
10	2.00E-03	8.00E-03	3.99	-75.833	0.8975	0.9054	0.9722	1.5214
11	2.00E-03	1.00E-02	4.99	-75.840	0.9231	0.9313	1.0000	1.5649
12	2.00E-03	1.20E-02	5.97	-75.848	0.9529	0.9614	1.0323	1.6155
13	2.00E-03	1.40E-02	6.97	-75.852	0.9656	0.9741	1.0460	1.6369
14	2.00E-03	1.60E-02	7.97	-75.856	0.9786	0.9872	1.0601	1.6589
15	2.00E-03	1.80E-02	8.97	-75.859	0.9912	1.0000	1.0738	1.6804
16	2.00E-03	2.00E-02	9.97	-75.862	1.0000	1.0089	1.0833	1.6952
17	2.00E-03	2.20E-02	10.97	-75.862	1.0000	1.0089	1.0833	1.6952
18	2.00E-03	2.48E-02	12.39	-75.865	1.0126	1.0216	1.0970	1.7167

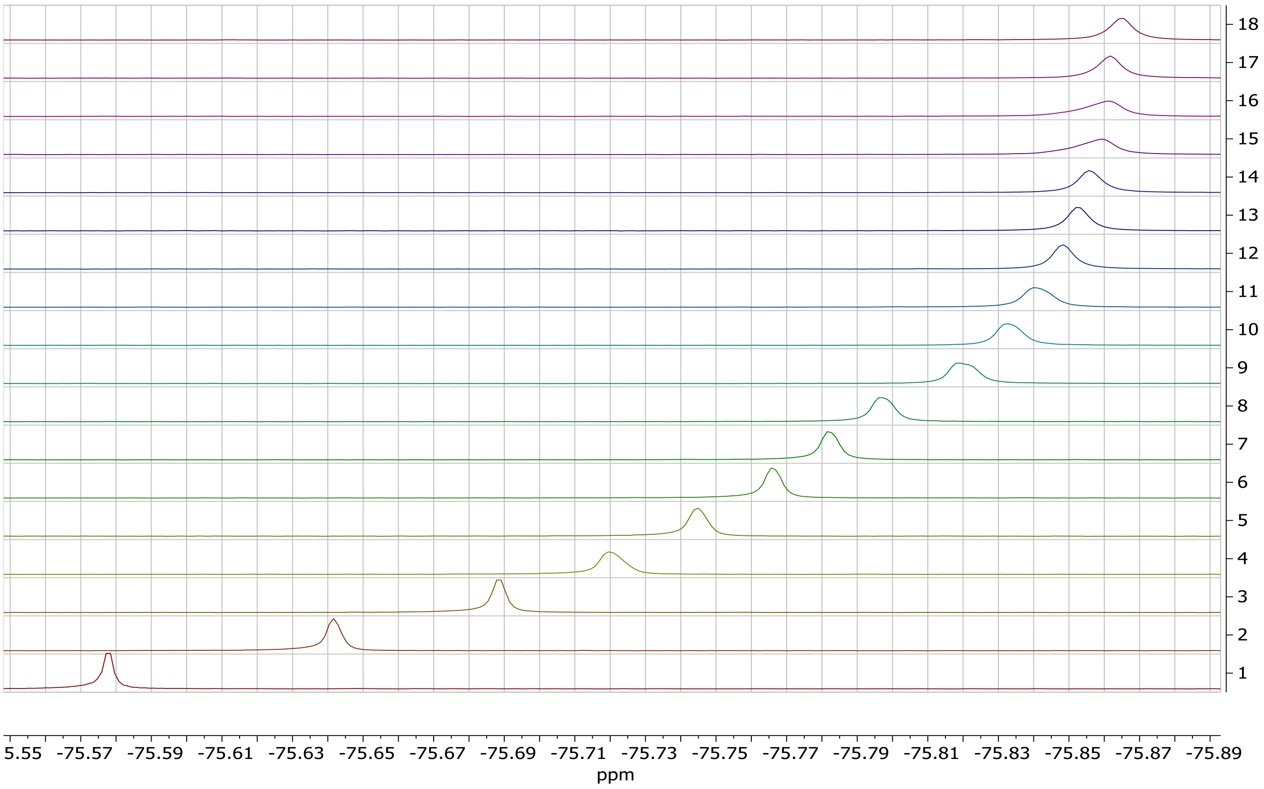

Table S14. ^{19}F NMR titration data for 2 mM trifluoroacetic acid (guest **16**) with **cycHC[8]**, in $CDCl_3$.

Nr.	Guest 16 concentration, M	cycHC[8] concentration, M	molar ratio, equiv	F1, ppm	F1 normalized at 5 equiv
1	1.99E-03	0.00E+00	0.00	-75.608	0.0000
2	1.99E-03	5.01E-04	0.25	-75.665	0.2692
3	1.99E-03	9.95E-04	0.50	-75.706	0.4615
4	1.99E-03	1.50E-03	0.75	-75.733	0.5895
5	1.99E-03	2.10E-03	1.06	-75.755	0.6920
6	1.99E-03	2.71E-03	1.36	-75.769	0.7563
7	1.99E-03	3.51E-03	1.77	-75.785	0.8333
8	1.99E-03	5.54E-03	2.79	-75.804	0.9230
9	1.99E-03	7.56E-03	3.81	-75.815	0.9745
10	1.99E-03	9.58E-03	4.82	-75.820	1.0000
11	1.99E-03	1.16E-02	5.82	-75.826	1.0255
12	1.99E-03	1.36E-02	6.84	-75.828	1.0383
13	1.99E-03	1.56E-02	7.86	-75.828	1.0383
14	1.99E-03	1.76E-02	8.85	-75.831	1.0510
15	1.99E-03	1.96E-02	9.85	-75.831	1.0510
16	1.99E-03	2.04E-02	10.25	-75.831	1.0510

Table S15. Second independent ^{19}F NMR titration data for 2 mM trifluoroacetic acid (guest **16**) with **cycHC[8]**, in $CDCl_3$

Nr.	Guest 16 concentration, M	cycHC[8] concentration, M	molar ratio, equiv	F1, ppm	F1 normalized at 5 equiv	F1 normalized at 1 equiv
1	1.90E-03	0.00E+00	0.00	-75.442	0.0000	0.0000
2	1.90E-03	9.44E-05	0.05	-75.468	0.0719	0.1046
3	1.90E-03	1.90E-04	0.10	-75.487	0.1250	0.1819
4	1.90E-03	2.85E-04	0.15	-75.507	0.1820	0.2648
5	1.90E-03	3.81E-04	0.20	-75.526	0.2357	0.3430
6	1.90E-03	5.71E-04	0.30	-75.560	0.3308	0.4813
7	1.90E-03	7.62E-04	0.40	-75.589	0.4100	0.5964
8	1.90E-03	9.54E-04	0.50	-75.612	0.4762	0.6928
9	1.90E-03	1.33E-03	0.70	-75.649	0.5797	0.8434
10	1.90E-03	1.91E-03	1.00	-75.688	0.6874	1.0000
11	1.90E-03	2.67E-03	1.40	-75.720	0.7777	1.1314
12	1.90E-03	3.81E-03	2.00	-75.750	0.8599	1.2510
13	1.90E-03	5.71E-03	3.00	-75.776	0.9334	1.3580
14	1.90E-03	7.62E-03	4.00	-75.790	0.9732	1.4158
15	1.90E-03	9.53E-03	5.00	-75.800	1.0000	1.4548
16	1.90E-03	1.62E-02	8.52	-75.810	1.0291	1.4972

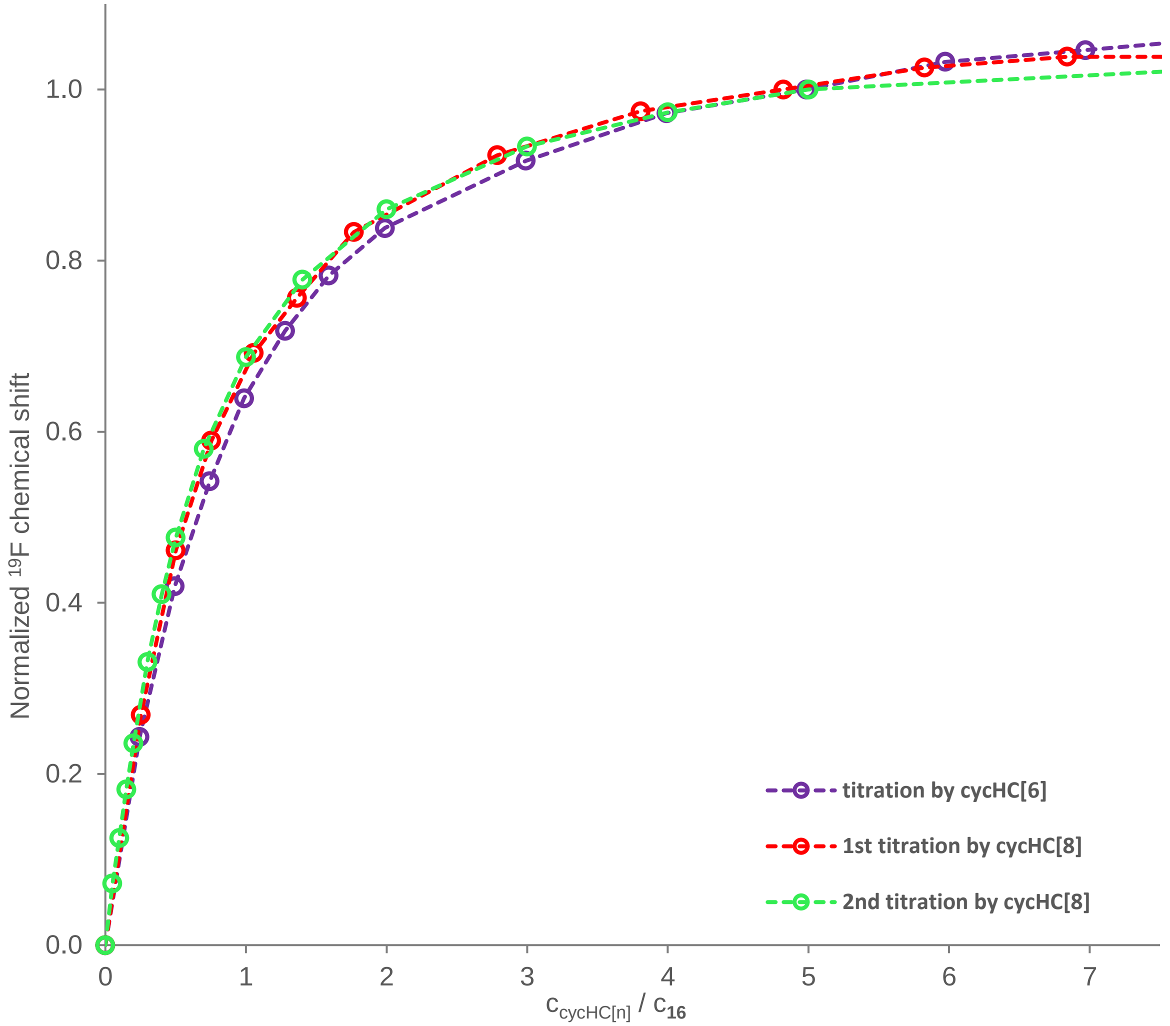

Figure S5. ^{19}F NMR titration data for trifluoroacetic acid **16** (2 mM) in the presence of growing concentration of **cycHC[6]** (purple) and **cycHC[8]** (red and green) normalized at ca 5 equiv. Experimental datapoints from **Table S13**, **Table S14**, **Table S15** are assigned with circles, the dotted lines are shown to guide the eye. More pronounced curves for titrations with **cycHC[8]** are suggesting stronger binding caused by larger amount of available binding sites (carbonyl groups) on **cycHC[8]** in comparison with **cycHC[6]**.

Table S16. ^{19}F NMR titration data for 18 mM trifluoroacetic acid (guest **16**) with **cycHC[6]**, in $CDCl_3$

Nr.	Guest 16 concentration, M	cycHC[6] concentration, M	molar ratio, equiv	F1, ppm	F1 normalized at 1.2 equiv	F1 normalized at 1 equiv
1	1.82E-02	0.00E+00	0.00	-75.569	0.0000	0.0000
2	1.82E-02	3.75E-04	0.02	-75.604	0.1232	0.1255
3	1.82E-02	7.47E-04	0.04	-75.635	0.2283	0.2324
4	1.82E-02	1.12E-03	0.06	-75.660	0.3151	0.3209
5	1.82E-02	1.49E-03	0.08	-75.681	0.3876	0.3946
6	1.82E-02	1.85E-03	0.10	-75.706	0.4710	0.4796
7	1.82E-02	2.22E-03	0.12	-75.717	0.5108	0.5201
8	1.82E-02	2.77E-03	0.15	-75.739	0.5836	0.5942
9	1.82E-02	3.67E-03	0.20	-75.764	0.6704	0.6826
10	1.82E-02	4.59E-03	0.25	-75.779	0.7212	0.7344
11	1.82E-02	5.49E-03	0.30	-75.790	0.7611	0.7749
12	1.82E-02	7.31E-03	0.40	-75.812	0.8369	0.8521
13	1.82E-02	9.12E-03	0.50	-75.826	0.8843	0.9004
14	1.82E-02	1.09E-02	0.60	-75.836	0.9169	0.9336
15	1.82E-02	1.46E-02	0.80	-75.846	0.9530	0.9703
16	1.82E-02	1.82E-02	1.00	-75.855	0.9821	1.0000
17	1.82E-02	2.18E-02	1.20	-75.860	1.0000	1.0182
18	1.82E-02	2.55E-02	1.40	-75.864	1.0148	1.0332
19	1.82E-02	2.91E-02	1.60	-75.867	1.0254	1.0440
20	1.82E-02	3.27E-02	1.79	-75.869	1.0326	1.0514
21	1.82E-02	3.59E-02	1.97	-75.872	1.0402	1.0591

Table S17. ^{19}F NMR titration data for 18 mM trifluoroacetic acid (guest **16**) with **cycHC[8]**, in $CDCl_3$

Nr.	Guest 16 concentration, M	cycHC[8] concentration, M	molar ratio, equiv	F1, ppm	F1 normalized at 1.2 equiv	F1 normalized at 1 equiv
1	1.82E-02	0.00E+00	0.00	-75.562	0.0000	0.0000
2	1.82E-02	3.62E-04	0.02	-75.597	0.1298	0.1308
3	1.82E-02	7.31E-04	0.04	-75.629	0.2480	0.2500
4	1.82E-02	1.09E-03	0.06	-75.654	0.3424	0.3451
5	1.82E-02	1.46E-03	0.08	-75.677	0.4289	0.4323
6	1.82E-02	1.83E-03	0.10	-75.696	0.4998	0.5038
7	1.82E-02	2.19E-03	0.12	-75.711	0.5550	0.5594
8	1.82E-02	2.73E-03	0.15	-75.729	0.6222	0.6271
9	1.82E-02	3.65E-03	0.20	-75.754	0.7165	0.7222
10	1.82E-02	4.56E-03	0.25	-75.770	0.7755	0.7816
11	1.82E-02	5.47E-03	0.30	-75.783	0.8228	0.8293
12	1.82E-02	7.29E-03	0.40	-75.799	0.8818	0.8887
13	1.82E-02	9.13E-03	0.50	-75.809	0.9213	0.9286
14	1.82E-02	1.10E-02	0.60	-75.816	0.9448	0.9523
15	1.82E-02	1.46E-02	0.80	-75.824	0.9765	0.9842
16	1.82E-02	1.83E-02	1.00	-75.828	0.9922	1.0000
17	1.82E-02	2.19E-02	1.20	-75.830	1.0000	1.0079
18	1.82E-02	2.50E-02	1.37	-75.832	1.0078	1.0158

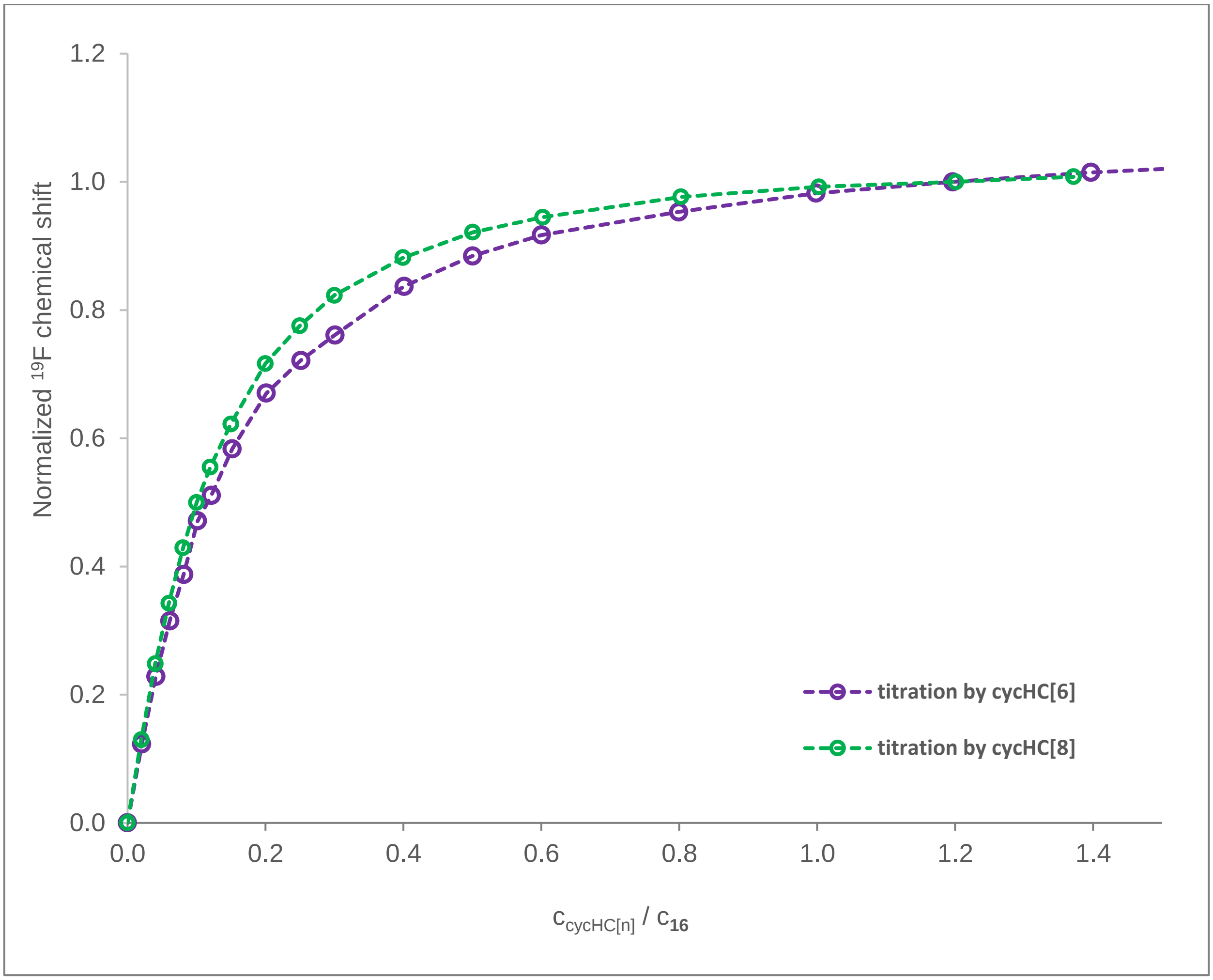

Figure S6. ^{19}F NMR titration data for trifluoroacetic acid **16** (18.2 mM) in the presence of growing concentration of **cycHC[6]** (purple) and **cycHC[8]** (green) normalized at 1.2 equiv. Experimental datapoints from, **Table S16**, **Table S17** are assigned with circles, the dotted lines are shown to guide the eye. More pronounced curve for titration with **cycHC[8]** is suggesting stronger binding caused by larger amount of available binding sites (carbonyl groups) on **cycHC[8]** in comparison with **cycHC[6]**. Results corresponds to data obtained at 2 mM concentration of **16** (**Figure S5**).

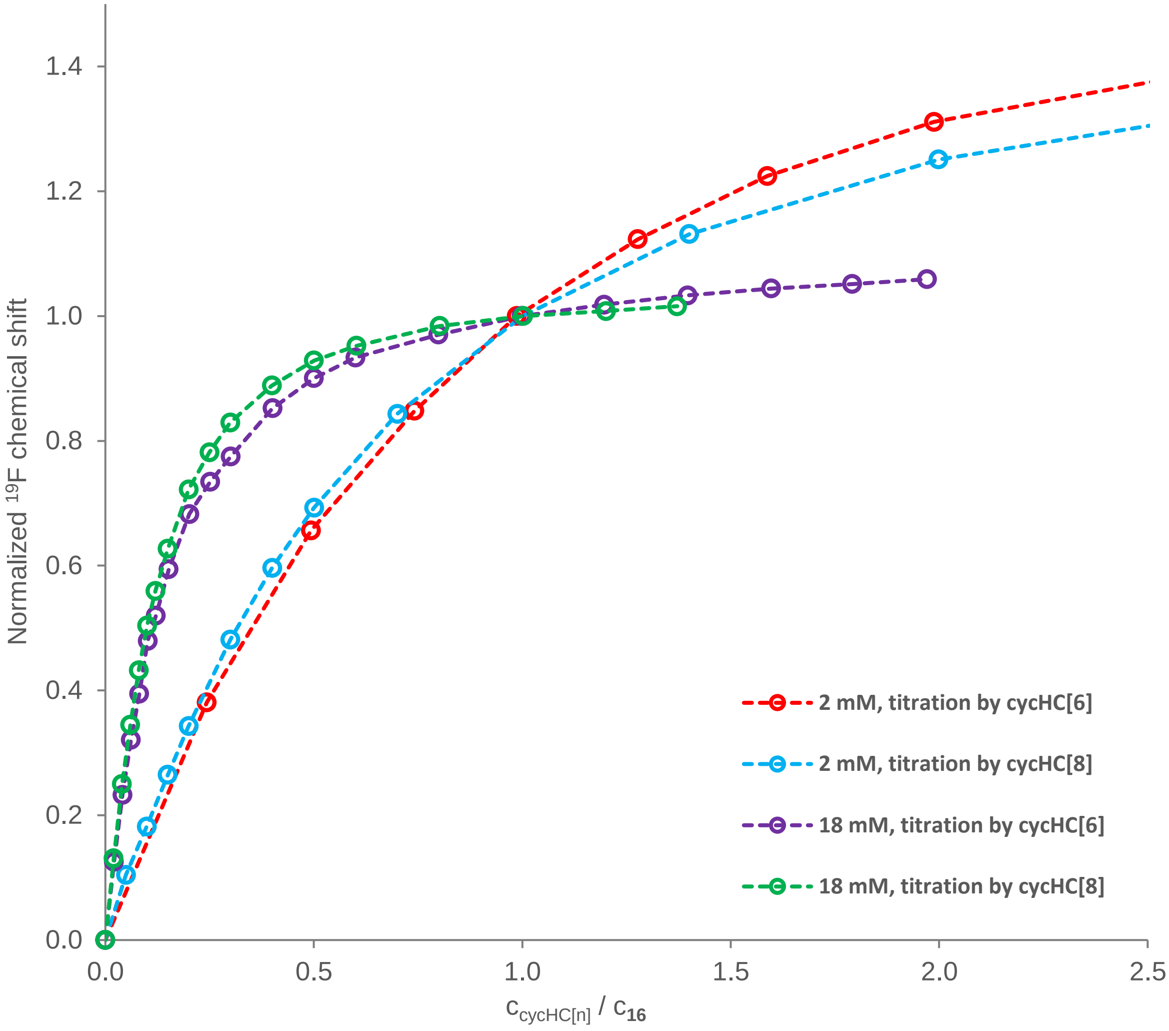

Figure S7. ^{19}F NMR titration data for different concentrations of trifluoroacetic acid **16** in the presence of growing concentration of **cycHC[6]** (red, purple) and **cycHC[8]** (blue, green) normalized at 1.0 equiv. Experimental datapoints from **Table S13**, **Table S15**, **Table S16**, **Table S17** are assigned with circles, the dotted lines are shown to guide the eye. Graph illustrates the change in the shape of titration curves related to roughly one order of magnitude difference in concentration of titrated compound (here **16**). Significant difference is caused by different proportion of formed complex as a natural consequence of constant value of binding constants at all concentrations.

Table S18. ^{1}H NMR titration data for 2 mM *S*-camphorsulfonic acid (guest **17**) with **cycHC[6]**, in $CDCl_3$ and corresponding spectra below.

Nr.	Guest 17 concentration, M	cycHC[6] concentration, M	molar ratio, equiv	H1, ppm	H1 normalized at 10 equiv	H2, ppm
1	2.00E-03	0.00E+00	0.00	3.1317	0.0000	0.9878
2	2.00E-03	1.01E-03	0.50	3.1297	0.0743	0.9860
3	2.00E-03	2.01E-03	1.00	3.1282	0.1301	0.9845
4	2.00E-03	3.00E-03	1.50	3.1266	0.1896	0.9832
5	2.00E-03	4.01E-03	2.00	3.1253	0.2379	0.9818
6	2.00E-03	6.01E-03	3.00	3.1227	0.3346	0.9790
7	2.00E-03	8.03E-03	4.01	3.1196	0.4498	0.9763
8	2.00E-03	1.20E-02	6.01	3.1148	0.6283	0.9711
9	2.00E-03	1.60E-02	8.02	3.1102	0.7993	0.9661
10	2.00E-03	2.01E-02	10.02	3.1048	1.0000	0.9612
11	2.00E-03	2.41E-02	12.03	3.0990	1.2156	0.9563
12	2.00E-03	2.81E-02	14.03	3.0927	1.4498	0.9517
13	2.00E-03	3.21E-02	16.03	3.0897	1.5613	0.9470
14	2.00E-03	3.61E-02	18.03	3.0828	1.8178	0.9428
15	2.00E-03	4.32E-02	21.57	3.0740	2.1450	0.9352

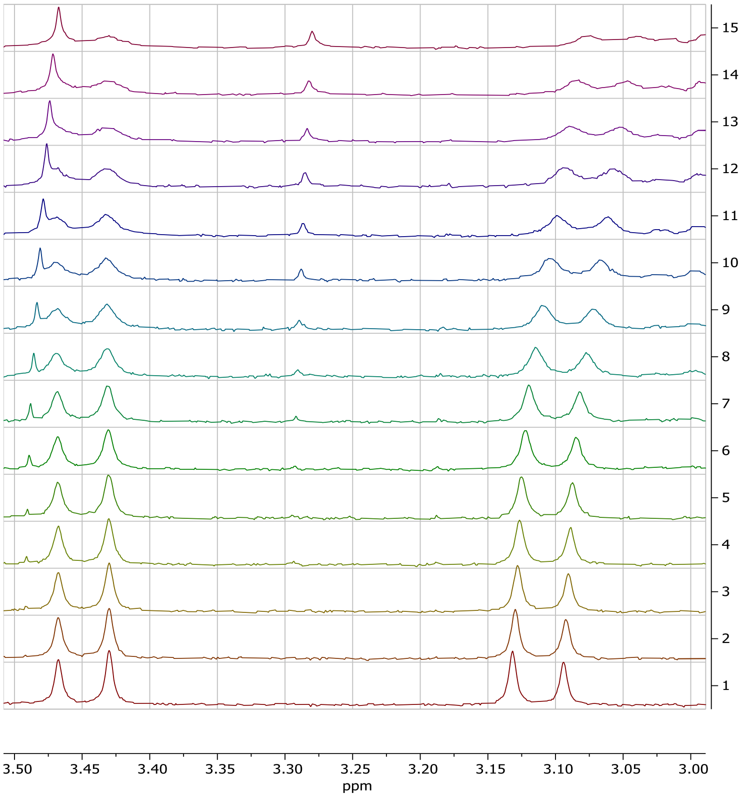

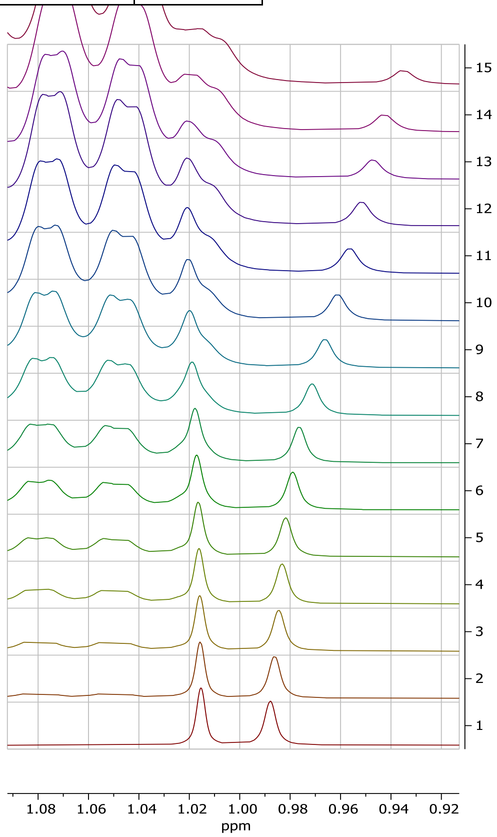

Table S19. ^{1}H NMR titration data for ca 0.4 mM methanesulfonic acid (guest **18**) with **cycHC[6]**, in $CDCl_3$. Concentration of **18** obtained from comparison of signals integration with **cycHC[6]**.

Guest 18 concentration, M	cycHC[6] concentration, M	molar ratio, equiv	H1, ppm	H1 recalc. to start from 0	normalization around 5 eq	normalization around 9 eq
5.50E-04	0.00E+00	0.00	3.0952	0.0000	0.0000	0.0000
5.50E-04	2.01E-04	0.37	3.0733	0.0219	0.2486	0.2186
4.18E-04	3.02E-04	0.72	3.0587	0.0365	0.4143	0.3643
3.55E-04	4.02E-04	1.13	3.0473	0.0479	0.5437	0.4780
3.42E-04	6.03E-04	1.77	3.0332	0.0620	0.7037	0.6188
3.15E-04	8.05E-04	2.55	3.0232	0.0720	0.8173	0.7186
3.15E-04	1.01E-03	3.19	3.0161	0.0791	0.8978	0.7894
2.98E-04	1.41E-03	4.73	3.0071	0.0881	1.0000	0.8792
3.09E-04	2.01E-03	6.52	3.0003	0.0949	1.0772	0.9471
3.10E-04	2.82E-03	9.10	2.9950	0.1002	1.1373	1.0000
3.10E-04	4.01E-03	12.93	2.9900	0.1052	1.1941	1.0499
3.04E-04	6.03E-03	19.83	2.9854	0.1098	1.2463	1.0958
3.20E-04	8.05E-03	25.18	2.9832	0.1120	1.2713	1.1178
3.22E-04	1.05E-02	32.59	2.9824	0.1128	1.2804	1.1257

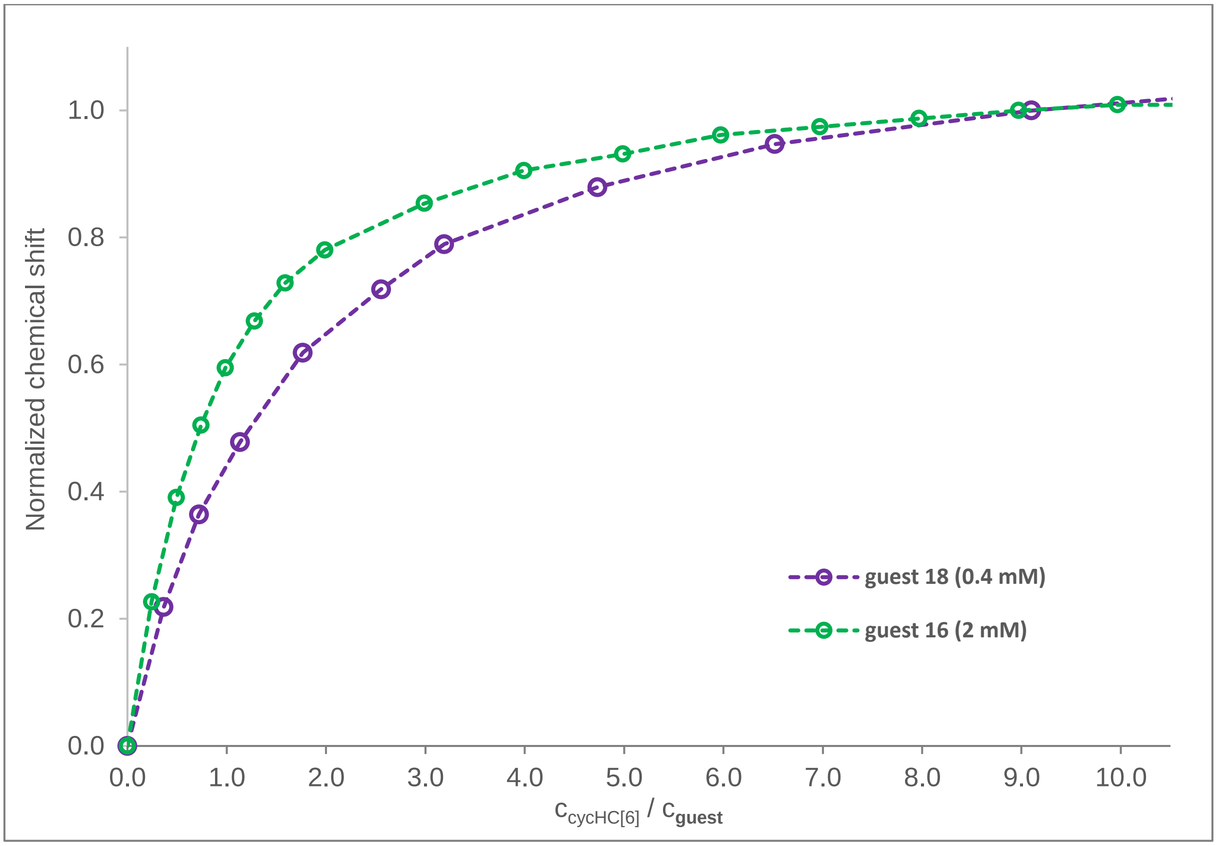

Figure S8. NMR titration data for trifluoroacetic acid **16** (2 mM, green) and methanesulfonic acid **18** (ca 0.4 mM, purple) in the presence of growing concentration of **cycHC[6]** normalized at 9 equiv. Experimental datapoints from **Table S13**, **Table S19** are assigned with circles, the dotted lines are shown to guide the eye. Although, titration curve of **16** is more pronounced than curve of **18** the concentration of **18** is roughly five-times lower, which has strong impact on the titration curve shape (proportion of formed complex as seen in **Figure S7**). Hence, we can speculate that **cycHC[6]** binds **18** similarly or rather stronger than **16**.

Table S20. ^{1}H NMR titration data for ca 2.2 mM methanesulfonic acid (guest **18**) with **cycHC[6]**, in $CDCl_3$. Concentration of **18** obtained from comparison of signals integration with **cycHC[6]**.

Guest 18 concentration, M	cycHC[6] concentration, M	molar ratio, equiv	H1, ppm	H1 recalc. to start from 0	normalization around 5 eq
1.36E-03	0.00E+00	0.00	3.1085	0.0000	0.0000
1.36E-03	5.19E-04	0.38	3.0563	0.0522	0.4254
1.61E-03	1.04E-03	0.65	3.0339	0.0746	0.6080
1.87E-03	1.56E-03	0.84	3.0217	0.0868	0.7074
1.98E-03	2.09E-03	1.06	3.0143	0.0942	0.7677
2.24E-03	2.61E-03	1.17	3.0090	0.0995	0.8109
2.36E-03	3.13E-03	1.33	3.0053	0.1032	0.8411
2.50E-03	3.65E-03	1.46	3.0024	0.1061	0.8647
2.50E-03	4.17E-03	1.67	2.9999	0.1086	0.8851
2.50E-03	5.21E-03	2.09	2.9965	0.1120	0.9128
2.50E-03	6.26E-03	2.50	2.9939	0.1146	0.9340
2.49E-03	8.33E-03	3.35	2.9904	0.1181	0.9625
2.56E-03	1.04E-02	4.06	2.9879	0.1206	0.9829
2.60E-03	1.25E-02	4.82	2.9858	0.1227	1.0000
2.49E-03	1.84E-02	7.40	2.9814	0.1271	1.0359

Table S21. ^{1}H NMR titration data for ca 0.3 mM methanesulfonic acid (guest **18**) with **cycHC[6]**, in $CDCl_3$. Concentration of **18** obtained from comparison of signals integration with standard (1,2,4,5-tetrachloro-3-nitrobenzene) having constant concentration throughout titration.

Guest 18 concentration, M	cycHC[6] concentration, M	molar ratio, equiv	H1, ppm	H1 recalc. to start from 0	normalization around 5 eq
2.48E-04	0.00E+00	0.00	3.0739	0.0000	0.0000
2.20E-04	3.53E-05	0.16	3.0692	0.0047	0.0646
2.25E-04	7.03E-05	0.31	3.0626	0.0113	0.1554
2.44E-04	1.17E-04	0.48	3.0548	0.0191	0.2627
2.70E-04	1.74E-04	0.64	3.0488	0.0251	0.3453
3.12E-04	2.31E-04	0.74	3.0438	0.0301	0.4140
3.44E-04	3.45E-04	1.01	3.0372	0.0367	0.5048
3.48E-04	4.61E-04	1.33	3.0316	0.0423	0.5818
3.85E-04	5.76E-04	1.50	3.0272	0.0467	0.6424
3.98E-04	6.91E-04	1.74	3.0228	0.0511	0.7029
4.18E-04	9.19E-04	2.20	3.0172	0.0567	0.7799
4.61E-04	1.38E-03	2.99	3.0096	0.0643	0.8845
4.56E-04	1.84E-03	4.03	3.0046	0.0693	0.9532
4.80E-04	2.30E-03	4.79	3.0012	0.0727	1.0000
4.70E-04	2.53E-03	5.38	2.9993	0.0746	1.0261
4.77E-04	2.70E-03	5.67	2.9980	0.0759	1.0440

Table S22. ^{1}H NMR titration data for ca 0.8 mM methanesulfonic acid (guest **18**) with **cycHC[8]**, in $CDCl_3$. Concentration of **18** obtained from comparison of signals integration with **cycHC[8]**.

Guest 18 concentration, M	cycHC[8] concentration, M	molar ratio, equiv	H1, ppm	H1 recalc to start from 0	normalization around 5 eq
6.60E-04	0.00E+00	0.00	3.0963	0.0000	0.0000
6.60E-04	1.02E-04	0.15	3.0793	0.0170	0.1797
6.94E-04	2.04E-04	0.29	3.0677	0.0286	0.3023
7.27E-04	3.05E-04	0.42	3.0592	0.0371	0.3922
7.44E-04	4.06E-04	0.55	3.0523	0.0440	0.4651
7.87E-04	5.13E-04	0.65	3.0461	0.0502	0.5307
7.74E-04	6.19E-04	0.80	3.0410	0.0553	0.5846
8.62E-04	8.24E-04	0.96	3.0334	0.0629	0.6649
8.81E-04	1.24E-03	1.40	3.0234	0.0729	0.7706
8.74E-04	2.06E-03	2.36	3.0129	0.0834	0.8816
8.55E-04	3.09E-03	3.62	3.0058	0.0905	0.9567
8.29E-04	4.12E-03	4.98	3.0017	0.0946	1.0000
8.45E-04	6.18E-03	7.32	2.9966	0.0997	1.0539
7.82E-04	8.26E-03	10.55	2.9931	0.1032	1.0909
8.03E-04	1.03E-02	12.88	2.9899	0.1064	1.1247

5.5 Evaluation of association constants with 1:1 and 2:1 binding model in Bindfit

Titration data for guests **6**, **9**, **10**, **11**, **12**, **13**, **14**, **15** and **17** was not possible to evaluate with 3:1 binding model due to their weak binding strength, therefore, we had to restrict our binding model to fit only K_{1obs} and K_{2obs} for other guests. We employed free online tool Bindfit (supramolecular.org) for comparing the quality of fit with 2:1 and 1:1 binding models (Thordarson, 2011; Hibbert and Thordarson, 2016). Generally, the weakest binding guests **6**, **11**, **12** and **17** provided unrealistic values of K_{2obs} and could be fitted only with 1:1 binding models. Guests **9**, **10**, **13**, **14** and **15** provided miscellaneous result without any clear trend corresponding to their order in our qualitative comparison. Mostly, it was possible to fit their NMR data with 2:1 and also 1:1 model obtaining similar quality of fit. In some cases (**9**, **10**), both models (2:1 and 1:1) provided reasonable fit; in other cases (**13**, **14**, **15**) it meant unrealistic values from 2:1 model and at the same time, regular sinusoidal distribution of residuals for 1:1 model, indicating it as an incorrect model. Overall, the binding between the guests and **cycHC[6]** cannot be compared in quantitative manner, as the data cannot be evaluated with the same binding model.

5.6 Evaluation of association constants with 2:1 and 3:1 binding model

Table S23. Summary of observed association constants (K_{obs}) from fitting with our script for 3:1 binding model and from fitting with online tool Bindfit (supramolecular.org) for 2:1 binding model.

	guest	macrocycle	fitted data source	guest concentration, mM	3:1 binding model fitting results			Bindfit 2:1 binding model fitting results[a]	
					K_{1obs}, M^{-1}	K_{2obs}, M^{-1}	K_{3obs}, M^{-1}	K_{1obs}, M^{-1}	K_{2obs}, M^{-1}
1.[b]	**16**	**cycHC[6]**	Table S13	2.0	600±100	350±50	30±30	930±17	500±38
2.[c]	**16**	**cycHC[6]**	Table S16	18.2	insufficient quality of fit			0.04±0.01	230 000±22 000
3.	**16**	**cycHC[8]**	Table S14	2.0	insufficient quality of fit			350±34	550±47
4.	**16**	**cycHC[8]**	Table S15	1.9	1 180±20	326±17	195±20	420±57	650±83
5.[c]	**16**	**cycHC[8]**	Table S17	18.2	insufficient quality of fit			0.05±0.01	140 000±10 000
6.[d]	**18**	**cycHC[6]**	Table S19	0.4	1 700±250	500±250	400±200	3 700±470	1 400±550
7.[d]	**18**	**cycHC[6]**	Table S20	2.2				130±2	14 000±270
8.	**18**	**cycHC[6]**	Table S21	0.4	insufficient quality of fit			990±37	7 300±920
9.	**18**	**cycHC[8]**	Table S22	0.8	1 211±150	1 175±100	470±120	240±16	11 000±960

Notes: [a] we used 2:1 NMR binding model with "full flavor", which do not assume any specific relation: (i) between chemical shifts of HG and HG_2; (ii) between stepwise association constants (K_1, K_2). We present all obtained values from 2:1 binding model, however, the quality of fit varied from good to insufficient. [b] Although the value and error of K_{3obs} are same (3:1 binding model), we obtained random distribution of residuals (**Figure S10**) indicating reliability of overall fit. [c] Fitting of data from line 2 and 5 for 18.2 mM trifluoroacetic acid (**16**) by 2:1 model provided insufficient quality of fit with sinusoidal shape of residuals. [d] Data from line 6 and 7 were fitted simultaneously to improve quality of fit with 3:1 binding model.

Online available Bindfit evaluations for all lines from **Table S23** can be found on following web addresses:

Line 1: http://app.supramolecular.org/bindfit/view/b8ae08bd-39dc-426e-a2df-c8ec7c1265a4

Line 2: http://app.supramolecular.org/bindfit/view/1cbea5d1-1702-4636-a0a5-6e47003e09b3

Line 3: http://app.supramolecular.org/bindfit/view/f32f7069-eb39-4bca-8dff-e5f06095064f

Line 4: http://app.supramolecular.org/bindfit/view/294bc989-4d61-42c3-bd2c-54af6e578baa

Line 5: http://app.supramolecular.org/bindfit/view/df10194d-4cab-42c1-98c2-753b97bfefe8

Line 6: http://app.supramolecular.org/bindfit/view/2b583a9b-c797-41a2-ac19-b2a04f82f1cb

Line 7: http://app.supramolecular.org/bindfit/view/5940a110-7a31-433d-9cb1-f998e42e1799

Line 8: http://app.supramolecular.org/bindfit/view/2b90c177-c074-4b06-8f81-959c9180e51e

Line 9: http://app.supramolecular.org/bindfit/view/64b9a9e2-a941-4ce4-8d16-a9d44d007717

Table S24. Experimental and calculated values of chemical shift from 3:1 binding model for titration data, which provided reasonable residuals and apparent association constants (K_{obs}). Data from **Table S19** and **Table S20** were fitted simultaneously and provided one set of K_{obs}. Calculated chemical shifts for complexes at specific stoichiometry are at the bottom of the table.

Table S13 data				Table S15 data				Table S19 data				Table S20 data				Table S22 data			
molar ratio, equiv	F1, ppm	F1 FIT (3:1), ppm	residuals, ppm	molar ratio, equiv	F1, ppm	F1 FIT (3:1), ppm	residuals, ppm	molar ratio, equiv	H1, ppm	H1 FIT (3:1), ppm	residuals, ppm	molar ratio, equiv	H1, ppm	H1 FIT (3:1), ppm	residuals, ppm	molar ratio, equiv	H1, ppm	H1 FIT (3:1), ppm	residuals, ppm
0.00	-75.577	-75.577	0.00E+00	0.00	-75.442	-75.442	0.00E+00	0.00	3.0739	3.0739	0.00E+00	0.00	3.0739	3.0739	0.00E+00	0.00	3.0963	3.0963	0.00E+00
0.24	-75.641	-75.641	4.11E-04	0.05	-75.468	-75.466	-1.96E-03	0.37	3.0520	3.0460	6.01E-03	0.38	3.0217	3.0211	6.24E-04	0.15	3.0793	3.0806	-1.34E-03
0.49	-75.687	-75.688	6.28E-04	0.10	-75.487	-75.488	1.12E-03	0.72	3.0374	3.0352	2.17E-03	0.65	2.9993	2.9970	2.33E-03	0.29	3.0677	3.0688	-1.15E-03
0.74	-75.720	-75.721	1.02E-03	0.15	-75.507	-75.508	1.06E-03	1.13	3.0260	3.0265	-5.11E-04	0.84	2.9871	2.9839	3.21E-03	0.42	3.0592	3.0597	-4.82E-04
0.99	-75.745	-75.744	-8.99E-04	0.20	-75.526	-75.527	7.06E-04	1.77	3.0119	3.0135	-1.64E-03	1.06	2.9797	2.9760	3.66E-03	0.55	3.0523	3.0524	-1.04E-04
1.28	-75.766	-75.765	-9.51E-04	0.30	-75.560	-75.560	-3.48E-04	2.55	3.0019	3.0041	-2.16E-03	1.17	2.9744	2.9706	3.81E-03	0.65	3.0461	3.0461	2.22E-05
1.59	-75.783	-75.781	-1.64E-03	0.40	-75.589	-75.588	-6.73E-04	3.19	2.9948	2.9970	-2.23E-03	1.33	2.9707	2.9669	3.76E-03	0.80	3.0410	3.0411	-5.59E-05
1.99	-75.797	-75.796	-8.50E-04	0.50	-75.612	-75.612	-5.78E-04	4.73	2.9858	2.9871	-1.33E-03	1.46	2.9678	2.9642	3.58E-03	0.96	3.0334	3.0331	3.26E-04
2.99	-75.818	-75.820	2.13E-03	0.70	-75.649	-75.649	-3.51E-04	6.52	2.9790	2.9781	8.80E-04	1.67	2.9653	2.9624	2.92E-03	1.40	3.0234	3.0229	5.44E-04
3.99	-75.833	-75.833	8.85E-04	1.00	-75.688	-75.688	1.02E-04	9.10	2.9737	2.9711	2.61E-03	2.09	2.9619	2.9598	2.11E-03	2.36	3.0129	3.0119	9.51E-04
4.99	-75.840	-75.842	2.13E-03	1.40	-75.720	-75.721	9.40E-04	12.93	2.9687	2.9653	3.43E-03	2.50	2.9593	2.9580	1.25E-03	3.62	3.0058	3.0050	8.21E-04
5.97	-75.848	-75.848	-4.51E-04	2.00	-75.750	-75.750	7.62E-04	19.83	2.9641	2.9602	3.85E-03	3.35	2.9558	2.9559	-7.11E-05	4.98	3.0017	3.0009	7.90E-04
6.97	-75.852	-75.852	3.55E-04	3.00	-75.776	-75.776	-4.81E-05	25.18	2.9619	2.9599	2.00E-03	4.06	2.9533	2.9545	-1.18E-03	7.32	2.9966	2.9962	4.21E-04
7.97	-75.856	-75.856	4.73E-05	4.00	-75.790	-75.789	-6.96E-04	32.59	2.9611	2.9597	1.37E-03	4.82	2.9512	2.9536	-2.38E-03	10.55	2.9931	2.9936	-5.05E-04
8.97	-75.859	-75.858	-8.70E-04	5.00	-75.800	-75.798	-1.97E-03									12.88	2.9899	2.9919	-1.97E-03
9.97	-75.862	-75.861	-1.20E-03	8.52	-75.810	-75.812	1.64E-03												
10.97	-75.862	-75.862	6.01E-04																
12.39	-75.865	-75.864	-9.22E-04																
Values of chemical shift (ppm) of a guest at complexes having stoichiometry 1:1 (δHG_1), 2:1 (δHG_2) and 3:1 (δHG_3)																			
δHG_1=-75.8820±0.0006<br>δHG_2=-75.90±0.03<br>δHG_3=-75.9±0.2				δHG_x=-75.8319±0.0004<br>we used constraint:<br>δHG_1=δHG_2=δHG_3				δHG_1=2.9491±0.004<br>δHG_2=2.9373±0.008<br>δHG_3=2.8887±0.05								δHG_1=2.984±0.001<br>δHG_2=2.984±0.002<br>δHG_3=2.97±0.05			

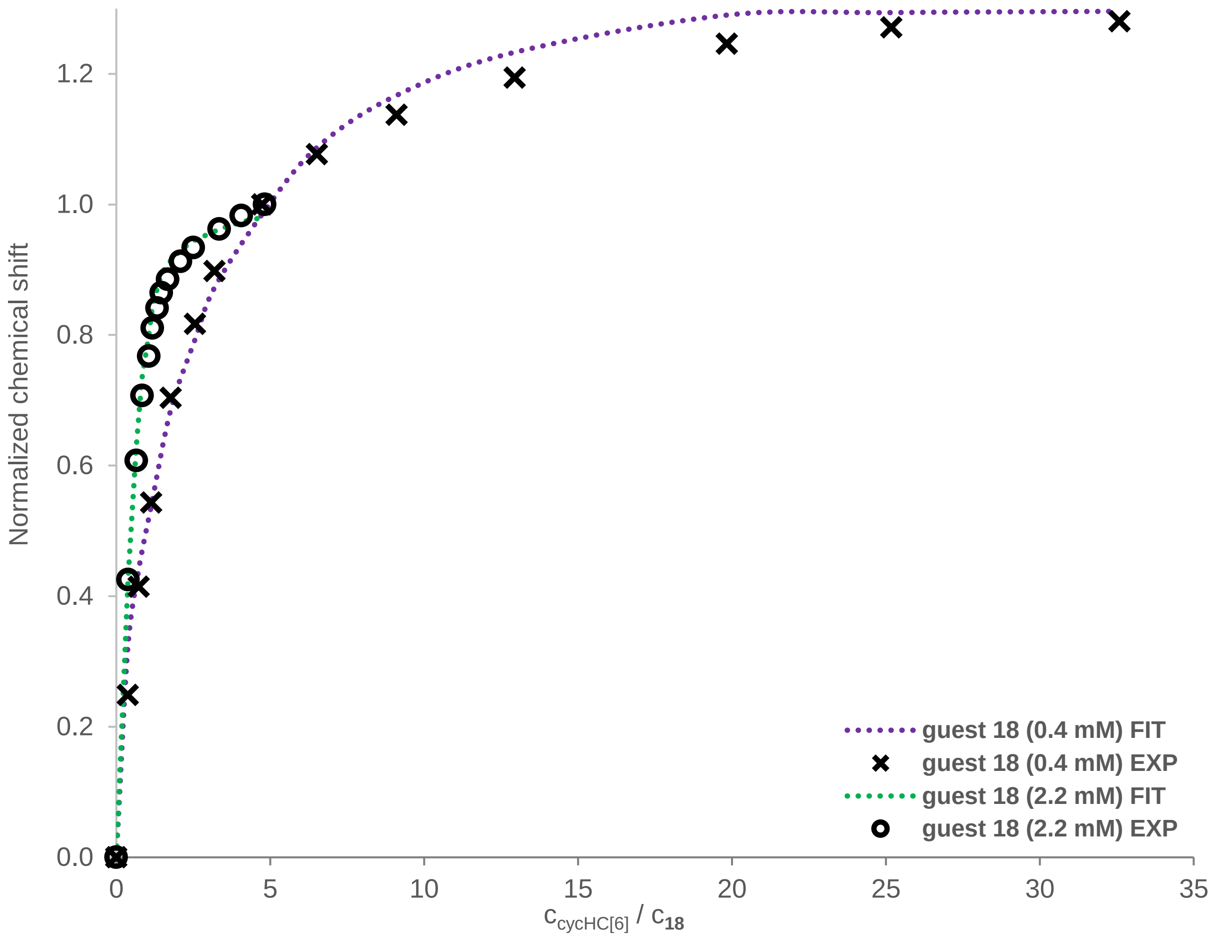

Figure S9. Normalization at 4.8 eq for data from **Table S19** (0.4 mM guest **18**) and **Table S20** (2.2 mM guest **18**), which were fitted simultaneously by the 3:1 binding model. Normalization of fitted binding isotherm was done using the value of experimentally obtained chemical shift at 4.8 eq.

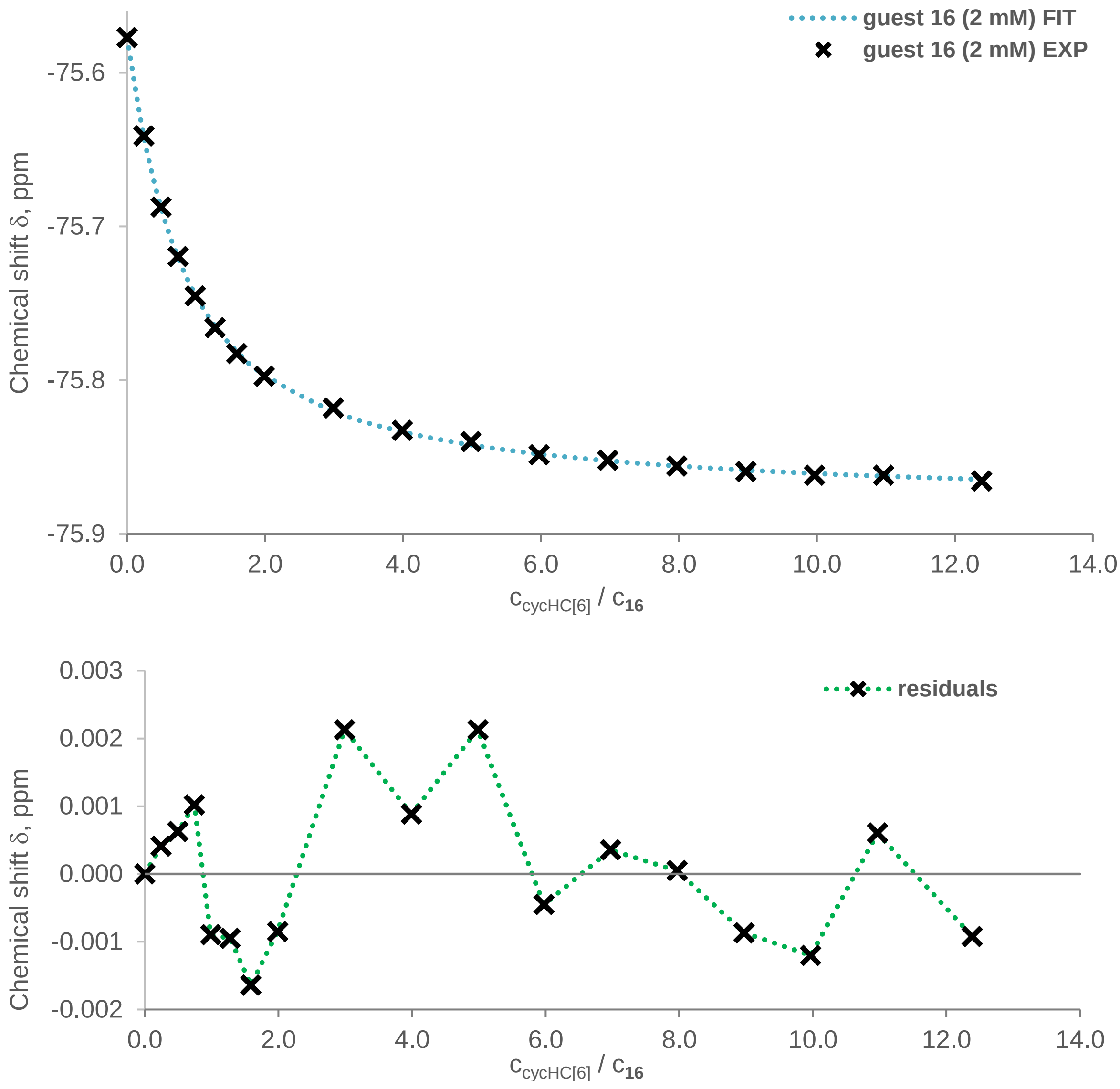

Figure S10. Fitting of data from **Table S13** (2 mM guest **16** titrated by **cycHC[6]**) with 3:1 binding model (**Table S23**, line 1). Obtained titration isotherm fits well with experimental points (upper graph), which is supported by random distribution of residuals (lower graph). Fitting provided apparent association constants K_{1obs} = 600 ± 100 M^{-1}, K_{2obs} = 350 ± 50 M^{-1} and K_{3obs} = 30 ± 30 M^{-1}, which can characterize the system only in this particular experimental conditions. As the quality of fit is similar to results of 2:1 binding model presented in main text of article, the 2:1 model should be assumed as a more reliable.

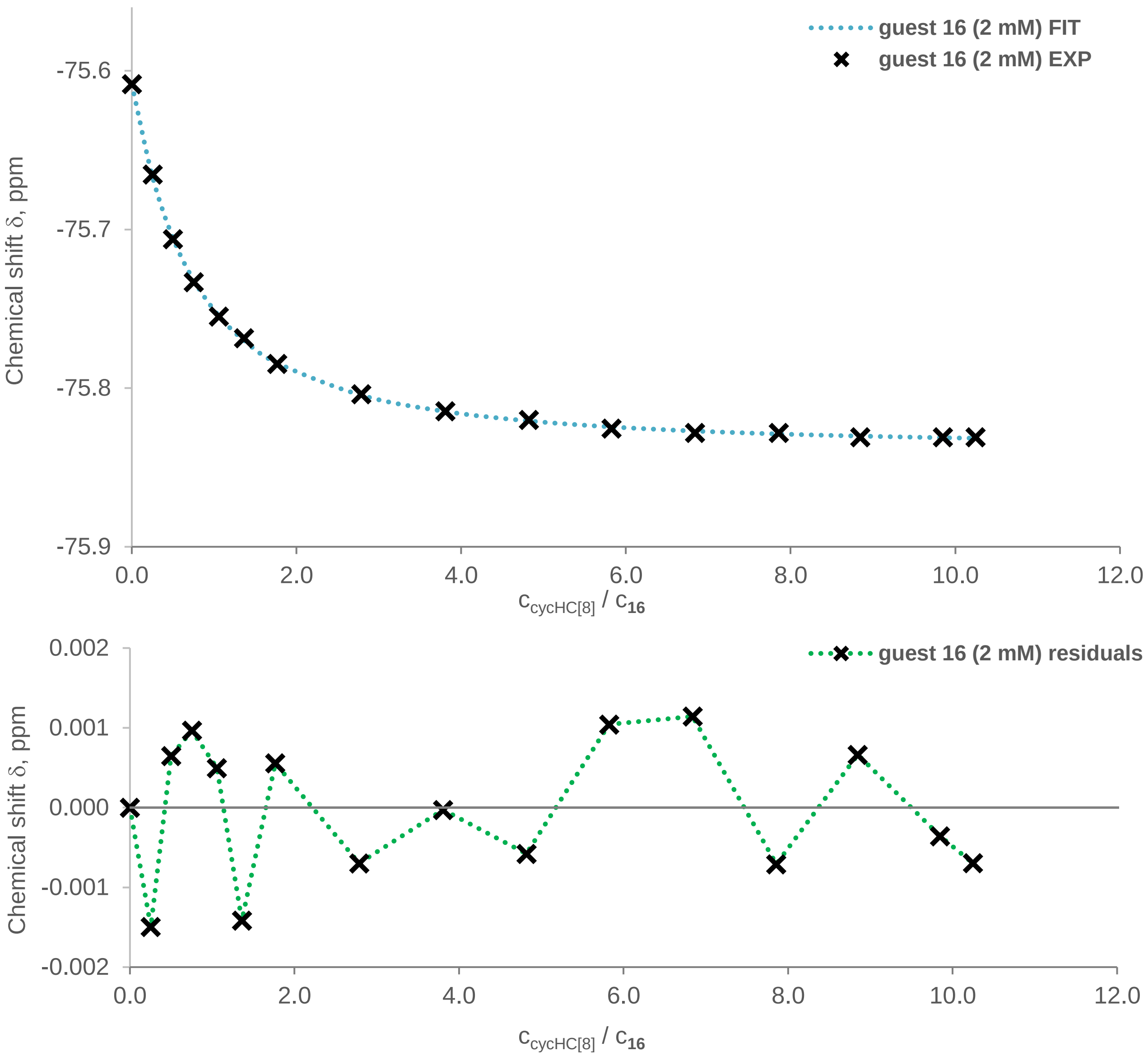

Figure S11. Fitting of data from **Table S14** (2 mM guest **16** titrated by **cycHC[8]**) with online tool Bindfit (supramolecular.org) for 2:1 binding model (**Table 23**, line 3). Obtained titration isotherm fits well with experimental points (upper graph), which is supported by random distribution of residuals (lower graph). Fitting provided apparent association constants K_{1obs} = 353 ± 34 M^{-1} and K_{2obs} = 550 ± 47 M^{-1}, which can characterize the system only in this particular experimental conditions. It can be seen from comparison with following **Figure S13**, that the same host-guest system could not be fitted at higher concentration of the guest.

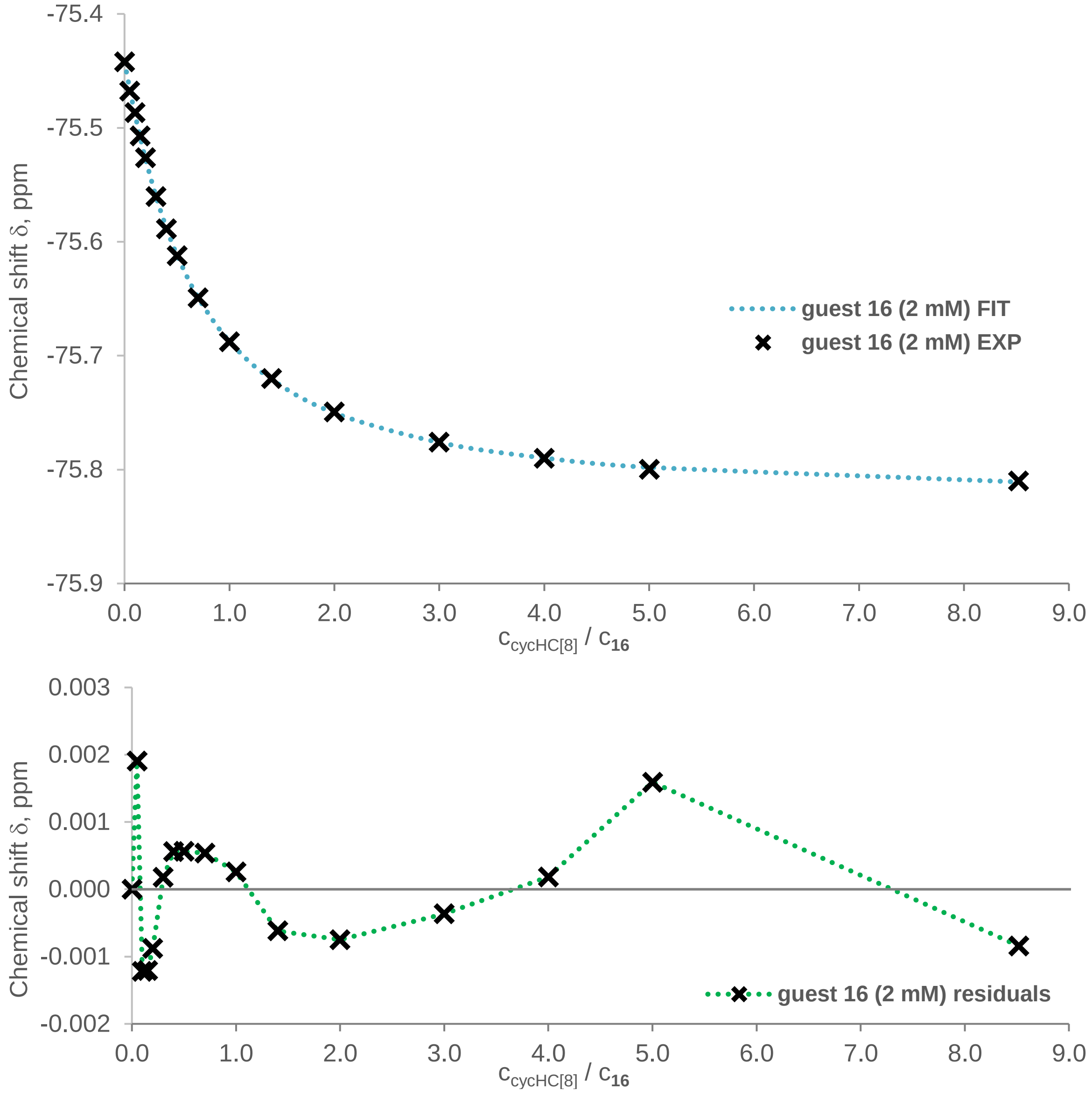

Figure S12. Fitting of data from **Table S15** (2 mM guest **16** titrated by **cycHC[8]**) with online tool Bindfit (supramolecular.org) for 2:1 binding model. (**Table S23**, line 4) Obtained titration isotherm fits reasonably with experimental points (upper graph), which is supported by irregular distribution of residuals (lower graph). Fitting provided apparent association constants K_{1obs} = 424 ± 57 M^{-1} and K_{2obs} = 653 ± 83 M^{-1}, which can characterize the system only in this particular experimental conditions. It can be seen from comparison with following **Figure S13**, that the same host-guest system could not be fitted at higher concentration of the guest.

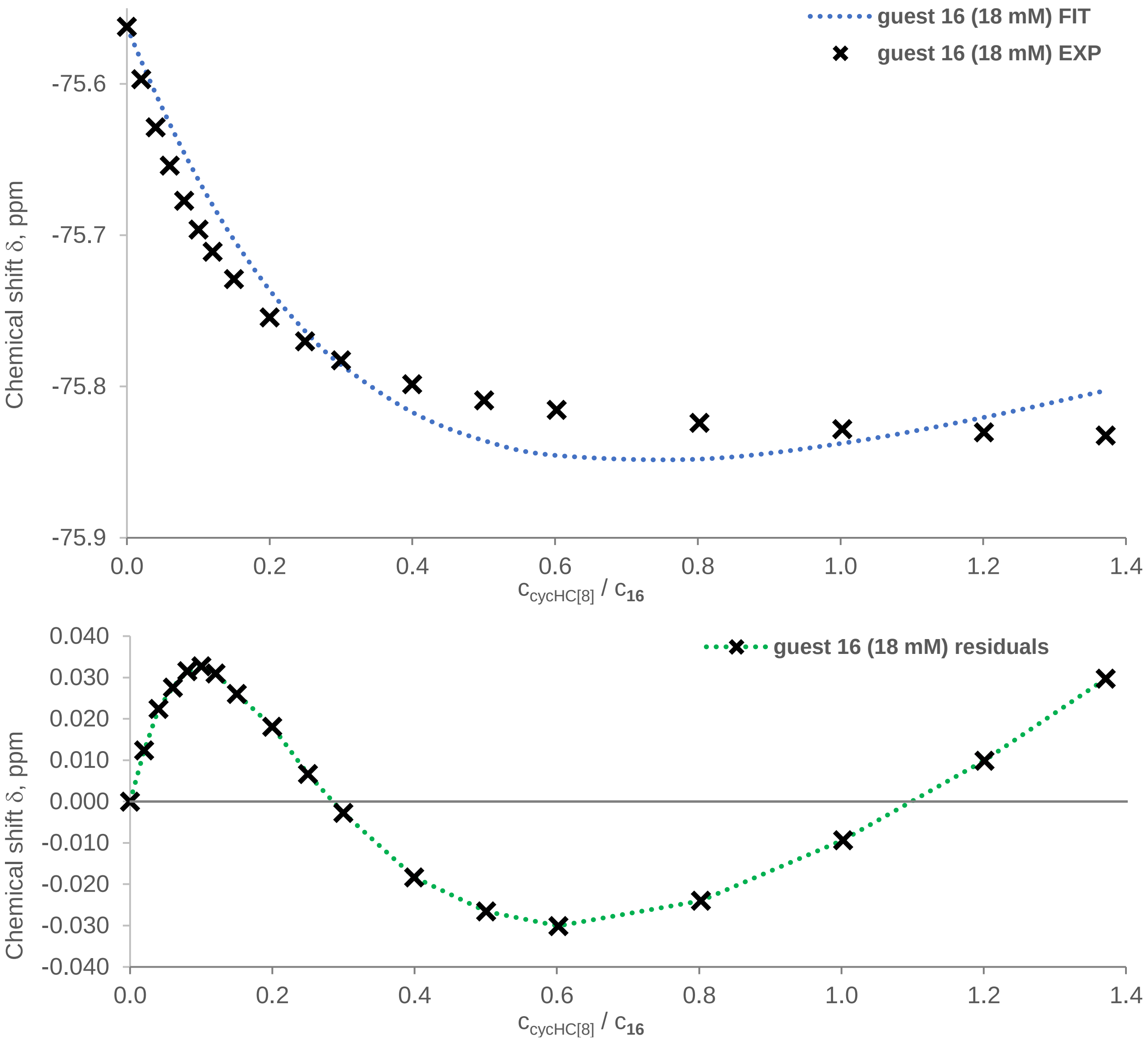

Figure S13. Fitting of data from **Table S17** (18.2 mM guest **16** titrated by **cycHC[8]**) with online tool Bindfit (supramolecular.org) for 2:1 binding model. Obtained titration isotherm, which does not fit with experimental points (upper graph), and sinusoidal distribution of residuals (lower graph) are evidence that 2:1 binding model is inappropriate for the system. The obtained K_{1obs} = 0.05 ± 0.01 M^{-1} and K_{2obs} = 138436 ± 9985 M^{-1} has no physical meaning. Large value of K_{2obs} in comparison with K_{1obs} indicates that real stoichiometry of the system could be higher than 2:1.

5.7 NMR data for continuous variation method (Job plot)

Table S25. ^{19}F NMR data for Job plot experiment at 10 mM total concentration of trifluoroacetic acid (guest **16**) and **cycHC[8]**, in $CDCl_3$.

Nr.	V, **cycHC[8]** (µl)	V, **16** (µl)	mole fraction x_i		δ, guest **16** (ppm)	Δδ (ppm)	Δδ·x_i(**16**)
			**cycHC[8]**	guest **16**			
1	0	600	0	1	-75.4389	0	0.000
2	60	540	0.1	0.9	-75.6393	0.2004	0.180
3	120	480	0.2	0.8	-75.7248	0.2859	0.229
4	180	420	0.3	0.7	-75.7654	0.3265	0.229
5	240	360	0.4	0.6	-75.7854	0.3465	0.208
6	300	300	0.5	0.5	-75.7997	0.3608	0.180
7	360	240	0.6	0.4	-75.8105	0.3716	0.149
8	420	180	0.7	0.3	-75.8192	0.3803	0.114
9	480	120	0.8	0.2	-75.8246	0.3857	0.077
10	540	60	0.9	0.1	-75.8294	0.3905	0.039
11	600	0	1	0	0		

Table S26. ^{19}F NMR data for Job plot experiment at 20 mM total concentration of trifluoroacetic acid (guest **16**) and **cycHC[6]**, in $CDCl_3$.

Nr.	V, **cycHC[6]** (µl)	V, **16** (µl)	mole fraction x_i		δ, guest **16** (ppm)	Δδ (ppm)	Δδ·x_i(**16**)
			**cycHC[6]**	guest **16**			
1	0	520	0.00	1.00	-75.5879	0	0.000
2	60	480	0.11	0.89	-75.7111	0.1232	0.110
3	65	455	0.13	0.88	-75.7205	0.1326	0.116
4	75	450	0.14	0.86	-75.7344	0.1465	0.126
5	85	425	0.17	0.83	-75.7479	0.16	0.133
6	100	400	0.20	0.80	-75.7682	0.1803	0.144
7	130	390	0.25	0.75	-75.7846	0.1967	0.148
8	170	340	0.33	0.67	-75.8055	0.2176	0.145
9	250	250	0.50	0.50	-75.8338	0.2459	0.123

6 Results of DFT calculations of partial atomic charges

Table S27. Calculated values of partial atomic charges on hydrogen bond donating functional groups of guests **6** and **9-18**.

Guest	functional group	Partial charge on O or S	Partial charge H
**6**	SH	-0.18534	0.15082114
**9**	OH	-0.38257	0.35637096
**10**	OH	-0.34637	0.34536613
**11**	SH	-0.16992	0.15300263
**12**	OH	-0.35604	0.34204295
**13**	OH	-0.60057	0.34158873
**14**	OH	-0.35474	0.35811029
**15**	OH	-0.38330	0.38294907
**16**	OH	-0.35129	0.37697190
**17**	OH	-0.46568	0.38232675
**18**	OH	-0.44975	0.38701379

7 References

Aav, R., Shmatova, E., Reile, I., Borissova, M., Topić, F., and Rissanen, K. (2013). New Chiral Cyclohexylhemicucurbit[6]uril. *Org. Lett.* 15, 3786–3789. doi:10.1021/ol401766a.

Aprà, E., Bylaska, E. J., de Jong, W. A., Govind, N., Kowalski, K., Straatsma, T. P., et al. (2020). NWChem: Past, present, and future. *J. Chem. Phys.* 152, 184102. doi:10.1063/5.0004997.

Becke, A. D. (1992). Density-functional thermochemistry. I. The effect of the exchange-only gradient correction. *J. Chem. Phys.* 96, 2155–2160. doi:10.1063/1.462066.

Ehrlich, S., Moellmann, J., Reckien, W., Bredow, T., and Grimme, S. (2011). System-Dependent Dispersion Coefficients for the DFT-D3 Treatment of Adsorption Processes on Ionic Surfaces. *ChemPhysChem* 12, 3414–3420. doi:https://doi.org/10.1002/cphc.201100521.

Ercolani, G. (2003). Assessment of Cooperativity in Self-Assembly. *J. Am. Chem. Soc.* 125, 16097–16103. doi:10.1021/ja038396c.

Frisch, M. J., Pople, J. A., and Binkley, J. S. (1984). Self-consistent molecular orbital methods 25. Supplementary functions for Gaussian basis sets. *J. Chem. Phys.* 80, 3265–3269. doi:10.1063/1.447079.

Grimme, S., Ehrlich, S., and Goerigk, L. (2011). Effect of the damping function in dispersion corrected density functional theory. *Journal of Computational Chemistry* 32, 1456–1465. doi:https://doi.org/10.1002/jcc.21759.

Halgren, T. A. (1996). Merck molecular force field. I. Basis, form, scope, parameterization, and performance of MMFF94. *Journal of Computational Chemistry* 17, 490–519. doi:https://doi.org/10.1002/(SICI)1096-987X(199604)17:5/6<490::AID-JCC1>3.0.CO;2-P.

Hanwell, M. D., Curtis, D. E., Lonie, D. C., Vandermeersch, T., Zurek, E., and Hutchison, G. R. (2012). Avogadro: an advanced semantic chemical editor, visualization, and analysis platform. *Journal of Cheminformatics* 4, 17. doi:10.1186/1758-2946-4-17.

Hibbert, D. B., and Thordarson, P. (2016). The death of the Job plot, transparency, open science and online tools, uncertainty estimation methods and other developments in supramolecular chemistry data analysis. *Chem. Commun.* 52, 12792–12805. doi:10.1039/C6CC03888C.

Hirshfeld, F. L. (1977). Bonded-atom fragments for describing molecular charge densities. *Theoret. Chim. Acta* 44, 129–138. doi:10.1007/BF00549096.

Kaabel, S., Stein, R. S., Fomitšenko, M., Järving, I., Friščić, T., and Aav, R. (2019). Size-Control by Anion Templating in Mechanochemical Synthesis of Hemicucurbiturils in the Solid State. *Angewandte Chemie International Edition* 58, 6230–6234. doi:10.1002/anie.201813431.

Lee, C., Yang, W., and Parr, R. G. (1988). Development of the Colle-Salvetti correlation-energy formula into a functional of the electron density. *Phys. Rev. B* 37, 785–789. doi:10.1103/PhysRevB.37.785.

Lu, T., and Chen, F. (2012a). Atomic dipole moment corrected hirshfeld population method. *J. Theor. Comput. Chem.* 11, 163–183. doi:10.1142/S0219633612500113.

Lu, T., and Chen, F. (2012b). Multiwfn: A multifunctional wavefunction analyzer. *Journal of Computational Chemistry* 33, 580–592. doi:https://doi.org/10.1002/jcc.22885.

Macrae, C. F., Bruno, I. J., Chisholm, J. A., Edgington, P. R., McCabe, P., Pidcock, E., et al. (2008). Mercury CSD 2.0 – new features for the visualization and investigation of crystal structures. *J Appl Cryst*, *J Appl Crystallogr* 41, 466–470. doi:10.1107/S0021889807067908.

Macrae, C. F., Edgington, P. R., McCabe, P., Pidcock, E., Shields, G. P., Taylor, R., et al. (2006). Mercury: visualization and analysis of crystal structures. *J Appl Cryst* 39, 453–457. doi:10.1107/S002188980600731X.

Macrae, C. F., Sovago, I., Cottrell, S. J., Galek, P. T. A., McCabe, P., Pidcock, E., et al. (2020). Mercury 4.0: from visualization to analysis, design and prediction. *J Appl Cryst* 53, 226–235. doi:10.1107/S1600576719014092.

Prigorchenko, E., Öeren, M., Kaabel, S., Fomitšenko, M., Reile, I., Järving, I., et al. (2015). Template-controlled synthesis of chiral cyclohexylhemicucurbit[8]uril. *Chem. Commun.* 51, 10921–10924. doi:10.1039/C5CC04101E.

Stephens, P. J., Devlin, F. J., Chabalowski, C. F., and Frisch, M. J. (1994). Ab Initio Calculation of Vibrational Absorption and Circular Dichroism Spectra Using Density Functional Force Fields. *J. Phys. Chem.* 98, 11623–11627. doi:10.1021/j100096a001.

Thordarson, P. (2011). Determining association constants from titration experiments in supramolecular chemistry. *Chem. Soc. Rev.* 40, 1305–1323. doi:10.1039/C0CS00062K.

Ustrnul, L., Kaabel, S., Burankova, T., Martõnova, J., Adamson, J., Konrad, N., et al. (2019). Supramolecular chirogenesis in zinc porphyrins by enantiopure hemicucurbit[n]urils (n = 6, 8). *Chem. Commun.* 55, 14434–14437. doi:10.1039/C9CC07150D.

Vosko, S. H., Wilk, L., and Nusair, M. (2011). Accurate spin-dependent electron liquid correlation energies for local spin density calculations: a critical analysis. *Canadian Journal of Physics*. doi:10.1139/p80-159.
